# Signature Genes Selection and Functional Analysis of Astrocytoma Phenotypes: A Comparative Study

**DOI:** 10.3390/cancers16193263

**Published:** 2024-09-25

**Authors:** Anna Drozdz, Caitriona E. McInerney, Kevin M. Prise, Veronica J. Spence, Jose Sousa

**Affiliations:** 1 Sano—Centre for Computational Personalised Medicine-International Research Foundation, Czarnowiejska 36, 30-054 Kraków, Poland; j.sousa@sanoscience.org; 2 Patrick G. Johnson Centre for Cancer Research, Queen’s University Belfast, BT9 7AE Belfast, Ireland; c.mcinerney@qub.ac.uk (C.E.M.); k.prise@qub.ac.uk (K.M.P.);

**Keywords:** signature genes, feature selection, classification, machine learning, astrocytoma, brain cancer, microarrays, artificial intelligence, precision medicine, patient stratification

## Abstract

**Simple Summary:**

Novel cancer biomarker discoveries are enabled by the application and analysis of omics technologies. This vast quantity of high-dimensional data necessitates the implementation of feature selection for analysis. The mathematical basis of selection methods varies considerably, which may influence subsequent inference. The aim of the study was to identify signature gene sets of grade 2 and 3 astrocytoma (brain cancer) and determine their impact on the classification and discovery of biological patterns. The application of feature selection methods reduced the number of genes and led to an increase in classification accuracy. Notably, no single gene was selected by all methods. Significant differences in Gene Ontology terms as well as KEGG pathways were discovered. Results demonstrated a significant difference in outcomes when classification-type algorithms were utilised compared to mixed types (selection and classification). This may result in the inadvertent omission of biological phenomena, while simultaneously achieving enhanced classification outcomes.

**Abstract:**

Novel cancer biomarkers discoveries are driven by the application of omics technologies. The vast quantity of highly dimensional data necessitates the implementation of feature selection. The mathematical basis of different selection methods varies considerably, which may influence subsequent inferences. In the study, feature selection and classification methods were employed to identify six signature gene sets of grade 2 and 3 astrocytoma samples from the Rembrandt repository. Subsequently, the impact of these variables on classification and further discovery of biological patterns was analysed. Principal component analysis (PCA), uniform manifold approximation and projection (UMAP), and hierarchical clustering revealed that the data set (10,096 genes) exhibited a high degree of noise, feature redundancy, and lack of distinct patterns. The application of feature selection methods resulted in a reduction in the number of genes to between 28 and 128. Notably, no single gene was selected by all of the methods tested. Selection led to an increase in classification accuracy and noise reduction. Significant differences in the Gene Ontology terms were discovered, with only 13 terms overlapping. One selection method did not result in any enriched terms. KEGG pathway analysis revealed only one pathway in common (cell cycle), while the two methods did not yield any enriched pathways. The results demonstrated a significant difference in outcomes when classification-type algorithms were utilised in comparison to mixed types (selection and classification). This may result in the inadvertent omission of biological phenomena, while simultaneously achieving enhanced classification outcomes.

## 1. Introduction

The development of high-throughput technologies, especially omics (genomics, transcriptomics, proteomics, metabolomics), has provided a strong foundation for progressing our knowledge in modern systems biology. With the vast amount of data available, it is crucial to select those features that preserve the biological relevance of the data as well as the ones that can be used for therapeutic target discovery and classification purposes. In the case of cancer research, this task is of the utmost importance but is extremely challenging.

Tumours are characterised by great diversity in terms of genetic alterations, gene expression, mutations, and drug sensitivity, which poses a major therapeutic challenge. Moreover, tumour cells are constantly evolving at different rates for different tumour types. Identifying the features that drive and constrain these changes becomes crucial in understanding cancer progression.

This work focuses on astrocytoma, a subtype of adult diffuse gliomas (brain cancer) characterised by the IDH mutation. The grading of adult astrocytoma ranges from grade 2 to grade 4. These tumours are amongst the most common and malignant primary brain tumours affecting mostly adults in their late thirties, more often in men [1]. They form a diffuse infiltrating tumour with no definite border between healthy brain tissue, even though they may appear relatively well-marginated in imaging. Astrocytoma has a poor prognosis. For patients with grade 2, survival is on average over 5 years, and for grade 3 patients, it drops to 3 years [2]. The current standard of care for astrocytoma consists of tumour resection and chemoradiotherapy and to our knowledge only one drug, temozolomide (TMZ), is available for treatment. TMZ has been used in the treatment of astrocytoma since 2005 [3] and despite numerous studies on new therapies [4,5,6], none have proven successful, and therefore, TMZ remains the gold-standard.

According to current diagnostic criteria, necrosis and/or microvascular proliferation and CDKN2A/B homozygous deletion are considered indicative of grade 4 astrocytoma [7]. Currently, grade 2 and 3 astrocytoma are differentiated solely by histological assessment, which could be biased depending on the assessor [8]. Even though it has been shown that genetic evaluation could better determine tumour subtypes than histological assessment [9], genetic markers to distinguish grade 2 from grade 3 astrocytoma are currently not available. Thus, new molecular markers are urgently required for astrocytoma to allow for the development of diagnostic tests for early intervention, targeted treatments, and better patient management by applying a precision medicine approach.

Advances in high-throughput technologies provides access to very detailed highly dimensional data but with small sample sizes, making further analysis very challenging. Redundant and noisy data can mask fine biological patterns. Detecting and selecting informative features from high-dimensional data is a fundamental step in an analysis, but it is also a significant challenge for biologists and machine learning models. Many feature selection algorithms have been developed in recent years to address this issue [10,11]. It has been shown that feature selection, and consequently reducing the number of genes from microarray experiments, increases the performance of the sample classification process [12,13,14].

The feature selection process is traditionally used to reduce the dimensionality of the problem, in this case the number of genes of interest. The identification of the differentially expressed genes, or the genes that describe unique patterns in the given phenotype (signature genes), may be used not only to predict the class membership (malignant/non-malignant, identification of tumour subtypes [15], treatment applied in the cell culture), but also for either discovery of impacted pathways in new drug target discovery or for drug repurposing. The mathematical basis of different selection methods varies considerably [16], which can result in not only different numbers of genes being selected but also disparate genes. This may have a significant impact on the subsequent analytical steps for biological inference, treatment decision-making, and drug design or repurposing.

To address this issue, six different feature selection methods were investigated: Differential Gene Expression (DGE), STIR, Boruta, Random Forest (RF), LASSO regression and CACTUS. The study aimed to discover signature gene sets for low-grade astrocytoma. Gene expression data from brain tumour samples from the REMBRANDT (REpository for Molecular BRAin Neoplasia DaTa) cohort were analysed. Next, the accuracy of sample classification based on the selected signature datasets was assessed. Finally, the differences in biologically relevant information (gene ontology, pathways, etc.) obtained from the different sets of signature genes was explored.

## 2. Materials and Methods

### 2.1. Data

In this study, transcriptional profiling data of grade 2 and grade 3 astrocytoma brain tumour samples were analysed. The data were obtained from REMBRANDT (REpository for Molecular BRAin Neoplasia DaTa), a large clinically annotated cohort that is publicly available [17]. The full experimental details can be found in the original paper but in summary, RNA was extracted and profiled on an Affymetrix U133 Plus2 array, now Applied Biosystems, a brand of DNA microarray products sold by Thermo Fisher Scientific (Waltham, MA, USA). The clinical characteristics of the patients in the cohort are described in the Supplementary Materials (Table S1).

As this is a legacy dataset collected before the updated WHO grading guidelines for glioma from 2016, sample information for molecular markers such as IDH1/2 that determine tumour subtype is not always available. To address this issue, samples predicted to be IDH-wildtype (n = 5) by the machine learning algorithm developed by Nuechterlein et al. [18] were excluded. This method analyses genome-wide somatic copy number alteration data of diffuse glioma samples to predict their 1p/19q-codeletion and IDH mutational status. Molecular subtype labels can then be retrospectively assigned to tumour samples lacking this annotation. The algorithm was used to exclude samples that were IDH wildtype, and therefore, of a different subtype to astrocytoma, the focus of our study. Finally, 55 grade 2 and 52 grade 3 astrocytoma samples were analysed in this study.

### 2.2. Pre-Processing, Quality Control, Normalisation and Initial Inspection of the Data

The *simpleaffy* [19] R 4.4.1 binary for macOS 11 (Big Sur) and higher package was used to compute quality control (QC) measures, such as average background, scale factor, number of genes called present and 3′ to 5′ ratios. All samples passed QC based on these metrics. Raw data in the form of CEL files were processed using the *affy* R package from Bioconductor [20]. The Robust Multi-array Average (RMA) algorithm was implemented using the *justrma* function [21]. This analytical step included background correction of the raw data, $log_{2}$ transformation and finally quantile normalisation.

Transcriptional data of all samples were initially analysed to examine sample clustering by grade. To investigate dimensionality reduction and global patterns, a PCA (Principal Component Analysis) was implemented with the *stats* package from the base R [22] and a Uniform Manifold Approximation and Projection (UMAP) with the *umap* R package [23]. To select the best parameters for UMAP, a combined search was performed with the following parameters: minimum distance in the range of 0.01–0.61 (with 0.1 steps), number of neighbours in the range of 2–20 (with step 2) and five different distance metrics (Euclidean, manhattan, cosine, Pearson, Pearson2). The final model was generated for six neighbours with a distance of 0.41 using Euclidean distance. In addition, transcriptional data of all samples were analysed using hierarchical clustering implemented with *gplots* R package [24]. Samples were labelled by grade in the heatmap to assist with examining whether grades stratified based on differing expression patterns.

### 2.3. Overview of the Comparison of Feature Selection and Classification Methods

**Figure 1:**
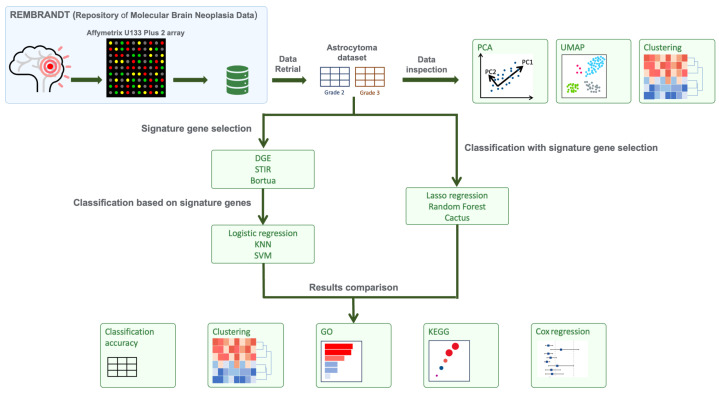
A schematic illustrating the workflow of the experiments, including: retrieval of data from the REMBRANDT repository, data pre-processing, data inspection, and the two branches of experiments comparing six different methods for feature selection and classification. In the first branch of experiments, DGE, STIR and Boruta methods were used to select signature gene sets, which were further analysed using three classification models: logistic regression, KNN and SVM. In the second set of experiments, RF, LASSO regression and CACTUS methods were used for signature gene selection and subsequent classification.

This study compared six different methods for feature selection of signature gene sets and classification of grade 2 and grade 3 astrocytoma brain tumour samples (Figure 1). The study was divided into two branches. In the first branch, three methods that only perform feature selection were implemented: Differential Gene Expression (DGE), STatistical Inference RelieF (STIR) [25], and Boruta algoritm [26]. Selected signature gene sets were then used as input into three different classification algorithms: k-Nearest Neighbours (KNN), Support Vector Machines (SVM) and logistic regression. In the second branch, mixed methods that can perform both feature selection and classification were applied. Methods included Random Forest (RF), LASSO regression, and CACTUS algorithms [27]. Finally, results from each of the methods were compared for classification accuracy, concordance of signature gene sets and their biological pathways as identified by gene set enrichment analyses for Gene Ontology (GO) and Kyoto Encyclopedia of Genes and Genomes (KEGG) pathways (Figure 1; see Section 2. Materials and Methods).

### 2.4. Signature Gene Selection

#### 2.4.1. Differential Gene Expression

Firstly, a differential gene expression analysis (DGE) was implemented utilising the Empirical Bayesian method from the *limma* R package [28]. Results were corrected for multiple hypothesis testing using the Benjamini–Hochberg method taking into consideration a 1% false discovery rate [29]. Differentially expressed genes were defined as being significant using either an adjusted *p*-value <0.01 or an expression fold change in $|\log_{2}FC|>1$.

#### 2.4.2. STIR-STatistical Inference RelieF

STatistical Inference RelieF (STIR) [25] is an extension of the RelieF [30,31] types of supervised machine learning for feature selection. RelieF algorithms consider the local and global feature space by first computing the nearest neighbours of a sample, then selecting the features that best discriminate the classes, and later updating the relevance of the features for the classification process. It has been suggested that RelieF is a good algorithm for feature spaces with a high degree of interaction, such as genomic and proteomic data [32]. With STIR [25] the calculation of the sample variance of the nearest neighbor distances is incorporated into the feature importance estimation. This allows for the determination of the statistical significance of features and an adjustment for multiple testing of RelieF-based scores, as well as establishing the significance cut-off for selected features. For the analysis herein, the *stir* package in R was used [25]. The number of *k* for the nearest neighbours search was estimated as the total number of samples divided by 6 (*k = num.samp/6*) and an adjusted *p*-value of 0.01 was used to determine statistical significance.

#### 2.4.3. Boruta Algorithm

The Boruta algorithm [26] is a supervised feature selection method that extends RF selection by iteratively comparing the actual predictor variables with generated shadow variables. Existing variables are randomly shuffled within each feature, making them uncorrelated with the target class. The true importance of the features is compared to the highest importance obtained from the shadow variables and evaluated if the difference is statistically significant. This approach provides a more accurate assessment of the importance of the features. Only features whose importance is statistically higher than the highest importance of the shadow variables are kept as important. For RF, the importance is given to all analysed features and the cut-off for feature importance is chosen arbitrarily. The Boruta algorithm was applied using the R package *Boruta* [26]. The dataset was split into training (n = 72) and testing sets (n = 35). The algorithm was ran with the default settings, with one exception for the *maxRuns* parameter, which was increased to 1000. This aimed to obtain more decisive results by reducing the number of *Tentative* classifications identified. The *TentativeRoughFix* function was applied to make the final decision about any tentative values. This function classifies all genes with median importance higher than the median importance of the maximal shadow attribute as being *Confirmed*.

### 2.5. Classification Based on Selected Signature Genes

The subset of genes selected by DGE, STIR and Boruta were further analysed for classification using KNN, SVM and logistic regression without penalisation, respectively, with the R packages *caret*, *e1071* and *glm* [33,34,35]. In addition, classification with the same methods was performed using the whole data set (all genes) without any pre-selection of genes. This aimed to determine whether pre-selection provided improvement to the model’s performance.

For each model’s evaluation, the data were split into training (n = 72) and testing sets (n = 35) and trained using a 10-fold cross-validation repeated three times. To additionally check if the feature selection improves the classification, the whole dataset (containing all genes) was analysed by applying the same methods.

For KNN models, different *k* values were tested using each odd value between 1 and 10 ($\sqrt{\mathrm{number}\;\mathrm{of}\;\mathrm{samples}}$). Cross-validation was used to determine the value of *k* that obtained the highest accuracy for the analysis of each signature gene sets. These optimal values for *k* were then used to derive the final models. Cross-validation revealed that the highest accuracy was obtained for *k* = 9 for all analyses with the exception of the DGE sample, where the optimal value was *k* = 7.

To train the SVM, different kernel types were tested: *linear, polynomial*, *radial*, and *sigmoid*. For all tested kernels, *svm* function parameters were kept as default. Results from the *radial* and *sigmoid* kernels were kept for the final classification as they provided the most accurate results.

### 2.6. Classification with Selection of Signature Genes

In the second branch of the study, methods with inherent feature selection and classification functionality were evaluated; these included RF, LASSO regression and CACTUS algorithms.

#### 2.6.1. Random Forest (RF)

RF is a supervised machine learning algorithm that examines multiple decision trees during training and combines them for making the final classification. To train the RF, the R package *caret* was used. The dataset was split into training (n = 72) and testing sets (n = 35) and models were trained using a 10-fold cross-validation performed three times. For training, the *ntree* parameter was kept at the default value and different numbers of *mtry* were iteratively tested, starting from 100 ($mtry=\sqrt{\mathrm{number}\;\mathrm{of}\;\mathrm{genes}}$) up to mtry=2. In each run, the number of genes was reduced by sub-setting the dataset with only those genes that had an importance>2 in the previous run. The final model was obtained by training the RF with the parameters which gave the highest accuracy in the preliminary training run. Following testing, it was determined that the RF model that gave the highest accuracy had 37 genes with an mtry=6.

#### 2.6.2. LASSO (Least Absolute Shrinkage and Selection Operator)

LASSO is a linear regression that uses shrinkage, whereby data values are shrunk to a central point (e.g., the mean) to produce simpler models. LASSO performs L1 regularisation, which applies a penalty reducing the values of the coefficients to prevent overfitting, increase accuracy and perform feature selection. The penalty is controlled by the value of *lambda* and may drive some coefficients to exactly zero thereby eliminating them from the model. To train the LASSO regression, the R package *glmnet* was used. The dataset was split into training (n = 72) and testing sets (n = 35) and models were trained using a 10-fold cross-validation performed three times. All parameters were kept as default except for the tuning parameter *lambda*. A search to find the optimal *lambda* was performed by testing values between 0 and 0.35 and using 0.01 as an increment. The final values used for the LASSO regression model were alpha=1 and lambda=0.01.

#### 2.6.3. CACTUS (Comprehensive Abstraction and Classification Tool for Uncovering Structures)

CACTUS is an explainable statistical learning framework developed for both feature selection and classification [27]. The CACTUS analysis consists of a series of steps. First, raw data are transformed into two-stage data abstractions (flips) based on the receiver operator curve (ROC) theory. For this dataset, genes/transcripts (flips) were coded as either *U* (“up”) or *D* (“down”) depending on their expression values relative to the calculated ROCs’ cut-off thresholds. This indicates that the raw value of the gene/transcript (flip) was above or below the calculated cut-off. Based on this, the single feature-based classification accuracy is calculated. Next, feature selection is performed using the margin error filtration (Equation (1)) of the z-score value, which can be chosen arbitrarily.

(1) $$Margin\;error=z\cdot \sqrt{\frac{p\cdot (1-p)}{n}}$$

*z*—selected z-score, *p*—single feature based classification accuracy, *n*—number of features in the dataset.

In the final step, gene significance is calculated from the conditional probability P($f|c_{i}$) of the flip *f*, given the class $c_{i}$ (grade 2 or grade 3). For each tumour grade, based on the corresponding gene significance $\sigma_{s,i}$ of each gene $x_{i}$ the value of a cost function $C_{s}$ was calculated according to Equation (2).

(2) $$C_{s}=\prod_{i=1}^{n}\;\sigma_{s,i}x_{i}$$

The cost function with the greatest value was the determinant for the final prediction of tumour grade. The feature selection was performed using a z-score=5 for the margin error filtration.

### 2.7. Comparison of the Overall Results

#### 2.7.1. Performance Metrics

To assess the classification’s performance and compare it between models the metrics, Sensitivity (Equation (3)), Specificity (Equation (4)), and Balanced Accuracy (BA; Equation (5)) were used. BA was preferable to simple accuracy because it is a more robust metric when sample sizes differ between the groups analysed.

(3) $$Sensitivity=\frac{TP}{TP+FN}$$

*TP*—True Positives, *FN*—False Negatives

(4) $$Specificity=\frac{TN}{TN+FP}$$

*TN*—True Negatives, *FP*—False Positives

(5) $$BA=\frac{Sensitivity+Specificity}{2}$$

#### 2.7.2. Concordance in the Signature Gene Sets

Concordance of the genes selected by each model was examined using a Venn diagram generated using *venn* and *ggvenn* libraries for R. Additionally, selected signature genes and their spread in expression fold changes (*logFC*) were visualised as volcano plots using the *ggplot2* package for R. The potential of selected gene sets to stratify astrocytoma samples based on their expression differences between grade 2 and 3 was also examined using hierarchical clustering implemented with *gplots* R package [24] and visualised as heatmaps.

#### 2.7.3. Concordance in the Biological Pathways Identified by Gene Set Enrichment Analyses

The signature gene sets were analysed for their biological pathways using gene set enrichment analyses performed with *clusterProfiler* R package [36]. Enrichment in Gene Ontology (GO) terms for biological process (BP) and Kyoto Encyclopedia of Genes and Genomes (KEGG) pathways was assessed. Results were corrected for multiple hypothesis testing using the Benjamini–Hochberg method and an adjusted *p*-value <0.01 for GO analysis and *p*-value <0.05 for KEGG. The top 15 most significantly enriched GO terms and all enriched KEGG pathways were visualised as plots using base R. Concordance in GO terms and KEGG pathways were examined using a Venn diagram as previously described.

Additionally, results revealed that the cell cycle was the most significantly enriched pathway for each of the signature gene sets. Thus, the cell cycle pathway and its genes identified to be differentially expressed in astrocytoma grades 2 and 3 were visualised as a pathway enrichment graph using *pathview* package for R [36]. In the network, genes identified by the models are indicated using a colour gradient for their fold change in expression for up- (green) and down-regulation (purple). In addition, the concordance in the genes identified from the cell cycle pathway by the models was compared using a Venn diagram.

#### 2.7.4. Signature Gene Sets as Prognostic Biomarkers

The selected signature gene sets were further evaluated for their potential as prognostic biomarkers. First, a survival analysis was performed for grade 2 versus grade 3 astrocytoma samples using a log-rank test and *p*-value < 0.05 for significance. Results were plotted as a Kaplan–Meier curve with the risk table included. Next, a multivariate Cox regression analysis was performed to examine the differences between the hazard ratios (HR) for grade 2 and 3 samples adjusted for the expression level of the given signature gene. For the selected signature genes, only genes that met the proportional hazards assumption evaluated using Schoenfeld residuals were included in the analysis. All analyses were performed using the *survival* package for R [37].

## 3. Results

### 3.1. Data Overview

The cohort analysed consisted of 27 females and 56 males and 24 without information on sex that ranged in age classes from 15–19 to 75–79 (Table S1). Almost an equivalent number of samples were compared in the analysis of grade 2 (n = 52) and grade 3 (n = 55) astrocytoma.

**Figure 2:**
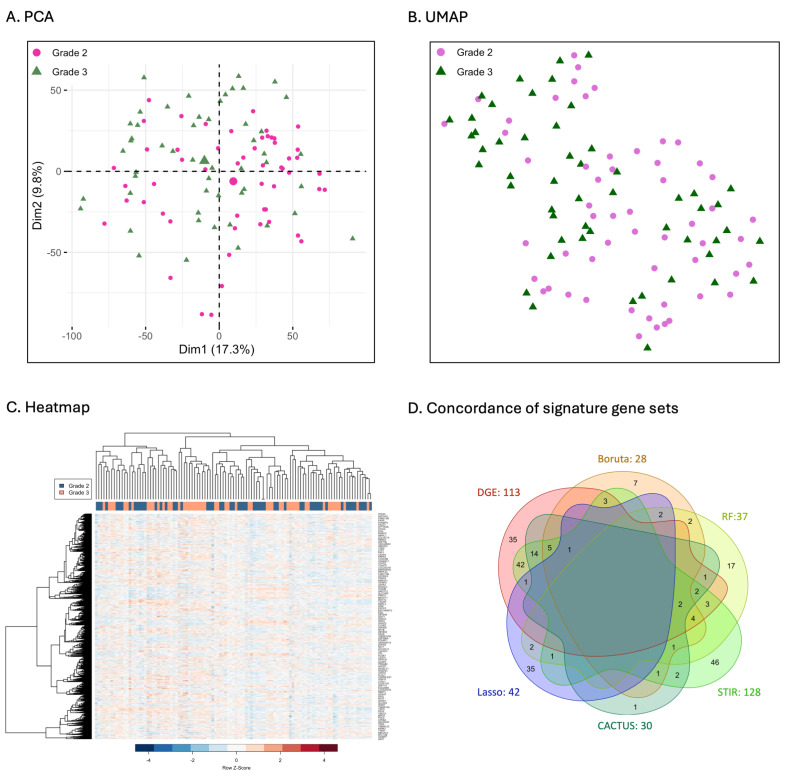
Initial evaluation of the transcriptional profiles of grade 2 and 3 astrocytoma samples examined using PCA (**A**), UMAP (**B**) and hierarchical clustering (**C**) with expression levels of the genes displayed as a z-score on a scale from high (red) to low (blue) values. Concordance between the signature gene sets identified by each of the models: DGE, Boruta, RF, STIR, CACTUS, and LASSO (**D**).

After an initial preprocessing of the raw data, the final dataset contained 10,096 genes, of which 4238 were up-regulated and 5858 were down-regulated. Results revealed that grade 2 and 3 astrocytoma had very similar expression profiles (Figure 2). Samples mostly overlapped and did not form discrete clusters in either the PCA or the UMAP plots. Also, a very low proportion of variance was explained by the first two dimensions of the PCA (17.3%, 9.8%) providing further evidence. Hierarchical clustering also indicated that transcriptional profiles of grade 2 and 3 astrocytoma were almost indistinguishable utilising the entire dataset. Samples did not stratify by grade onto separate branches in the heatmap, indicating that the majority of genes exhibited similar expression patterns for grade 2 and 3 astrocytoma.

### 3.2. Comparison of Signature Genes Selection

**Figure 3:**
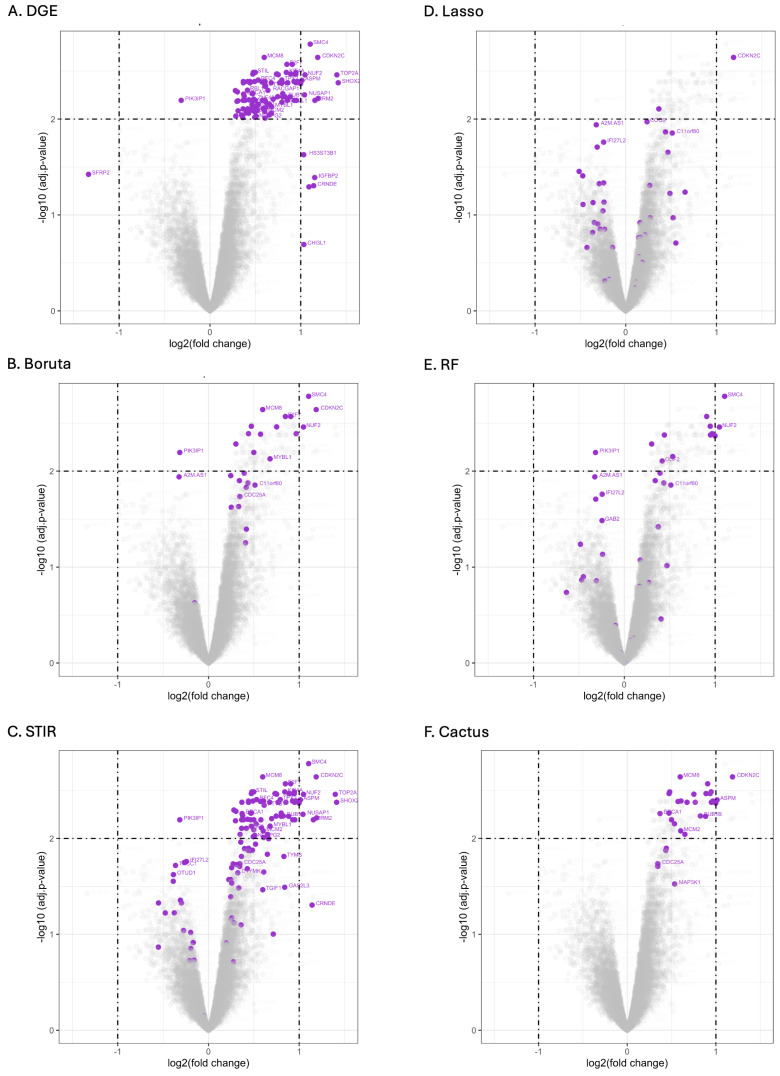
Volcano plots comparing the distribution of up- and down-regulated genes within signature gene sets selected by the following methods: DGE (**A**), Boruta (**B**), STIR (**C**), LASSO (**D**), RF (**E**) and CACTUS (**F**). Purple points indicate genes selected by a given algorithm. The x-axis displays $Log_{2}FC$ in gene expression and the y-axis displays the log odds of a gene being differentially expressed with the adjusted *p*-value. The dashed lines indicate the significance thresholds for $Log_{2}FC$ thresholds for the adjusted *p*-values used for DGE.

Comparisons revealed that feature selection methods differed widely in the total number of signature genes identified. Genes differed in their identity with little concordance between methods. The greatest number of signature genes were identified by STIR (n = 128), followed by DGE (n = 113), LASSO (n = 42), RF (n = 37), CACTUS (n = 30), and Boruta (n = 28; Figure 2D). No single gene was selected by all the methods analysed. Only three genes were selected by five of the six methods (CCNB1, CDKN2C, GJC1) and twelve by four methods (ACYP1, CENPK, DEPDC1, FANCD2, GINS1, ITGB3BP, MCM8, NDC80, NUF2, PIK3IP1, PLK4, SMC4; Table 1). A greater number of genes was commonly selected by three (n = 26; Table S2) and two methods (n = 48; Table S2). The greatest number of unique genes was identified by STIR (n = 46), followed by DGE (n = 35), LASSO (n = 35), RF (n = 17), Boruta (n = 7) and CACTUS (n = 1). Overall DGE had the greatest number of genes in common with each of the other methods and with STIR in particular (n = 42; Figure 3C). There was little overlap in the features selected between the other five methods.

A comparison of the signature gene sets and their spread in up- and down-regulation revealed clear differences between models (Figure 3). For DGE, 113 significant genes were identified and only two of these were down-regulated (SFRP2, PIK3IP1; Figure 3A). The models with the results most similar to DGE were CACTUS and Boruta, given that they also selected relatively fewer genes below the DGE significance thresholds. For STIR, RF and especially LASSO, genes selected by these models were very often characterised by low $Log_{2}FC$ and adjusted *p*-values and were not identified by applying traditional statistics with DGE. Additionally, there is a predominance of up-regulated genes in the selection for Boruta and STIR. Similarly, CACTUS selected only up-regulated genes. For RF and LASSO there is a more equal balance between the number of up- and down-regulated genes selected. The signature gene sets of RF and Boruta differed with only 16 genes in common, and a greater number of genes having smaller *p*-values and being up-regulated for Boruta. It is interesting to note that both RF and LASSO selected at least one gene with a very small fold change in expression level, which is close to 0 on the x-axis (Figure 3; Table S3).

### 3.3. Comparison of Clustering of Astrocytoma Based on Gene Expression Patterns

**Figure 4:**
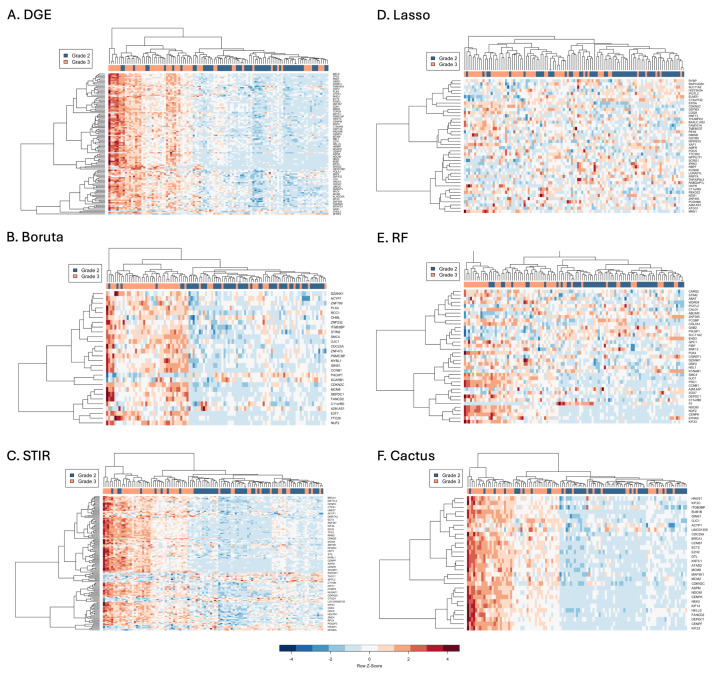
Heatmaps comparing the hierarchical clustering patterns and gene expression in grade 2 and 3 astrocytoma samples from the signature gene sets selected by the following methods: DGE (**A**), Boruta (**B**), STIR (**C**), LASSO (**D**), RF (**E**) and CACTUS (**F**). Expression levels of the selected genes are displayed as a z-score on a scale from high (red) to low (blue) values.

Signature gene sets were analysed using hierarchical clustering and their expression visualised in heatmaps (Figure 4). The signature genes selected by CACTUS were all upregulated in grade 3 compared to grade 2. These genes stratified samples into two main branches that each further divided to form four main groups with consistent expression (Figure 4F). On the main left branch, the two groups consisted of predominantly grade three samples with very high (dark red) and high expression (red). The main right branch was also split into two groups with the left group consisting of predominantly grade three samples with medium expression (pink), while the right group had predominantly grade two samples with the lowest expression for all genes (blue; Figure 4F).

DGE and Boruta exhibited similar hierarchical clustering patterns (Figure 4A,B). Each model identified an outgroup on a separate branch to the left, with the rest of the samples separating into two main branches that further divided. For each model, the outgroup consisted of five samples with the highest expression. All samples of the outgroup were comprised of grade 3 for DGE but one sample was grade 2 for Boruta. The other main branch was split into predominantly grade 3 samples with medium expression to the left and predominantly grade two samples with low expression to the right. Compared to CACTUS, expression patterns of the DGE and Boruta signature gene sets were less consistent between samples and also included both up- and down-regulated genes.

STIR and RF both had two main branches that further split to divide samples (Figure 4C,E). The left main branches consisted of mainly grade 3 samples. The right hand main branch split into mainly grade 3 on the left branch and mainly grade 2 on the right. STIR displayed mostly good stratification of samples into four main groups similar to CACTUS for the up-regulated genes but perhaps with more variability between samples. In addition, STIR also selected approximately a quarter of genes that were down-regulated in grade 3 compared to grade 2 and displayed greater variation between samples compared to up-regulated genes. By contrast, RF selected almost an equal number of up and down-regulated genes, which displayed a mixture of consistent and inconsistent patterns of expression between grades, suggesting that some selected features were perhaps better than others (Figure 4E). Boruta features certainly were more consistent and better able to stratify samples compared to RF (Figure 4B,E).

Hierarchical clustering revealed a poor performance by LASSO regression to stratify samples into distinct expression groups. Although an outgroup and two main branches were identified and membership of these was either predominantly grade 3 or 2, samples on branches displayed highly inconsistent expression (Figure 4B). This was further evident by the longer branch lengths compared to the other models, indicating more divergent expression patterns between samples on the same branch and a lack of sample stratification by expression.

### 3.4. Signature Genes Based Classification

Performance metrics and their range varied greatly between methods for sensitivity (0.462–0.923), specificity (0.462–0.769) and balanced accuracy (0.538–0.846; Table 2). Overall, the method with the highest balanced accuracy was KNN performed on the Boruta signature gene set (BA = 0.846, Table 2), followed by SVM (radial) performed on the Boruta signature gene set as well (0.808) and SVM (sigmoid) run with DGE signature gene set (0.808). Each of these analyses were characterised by high sensitivity (0.846, 0.923, 0.923).

For feature selection and classification algorithms, the best balanced accuracy was obtained when applying CACTUS (0.746) and the analysis was performed on 30 genes. This analysis was also characterised by high sensitivity (0.818) and the number of signature genes did not influence the classification accuracy.

It was observed that, for all purely classification methods, the pre-selection of signature genes improves the performance of algorithms resulting in higher accuracy when comparing with the classification based on the whole dataset with all genes. In the case of the KNN and logistic regression, keeping all genes has a negative impact on results as evidenced by lowered BA for each approach, respectively, to 0.692 and 0.538. By comparison RF, LASSO regression and CACTUS had higher BAs which were 0.691, 0.649 and 0.746, respectively.

### 3.5. Signature Genes and Their Pathways and Functions

#### 3.5.1. Gene Ontology (GO) Enrichment Analyses

**Figure 5:**
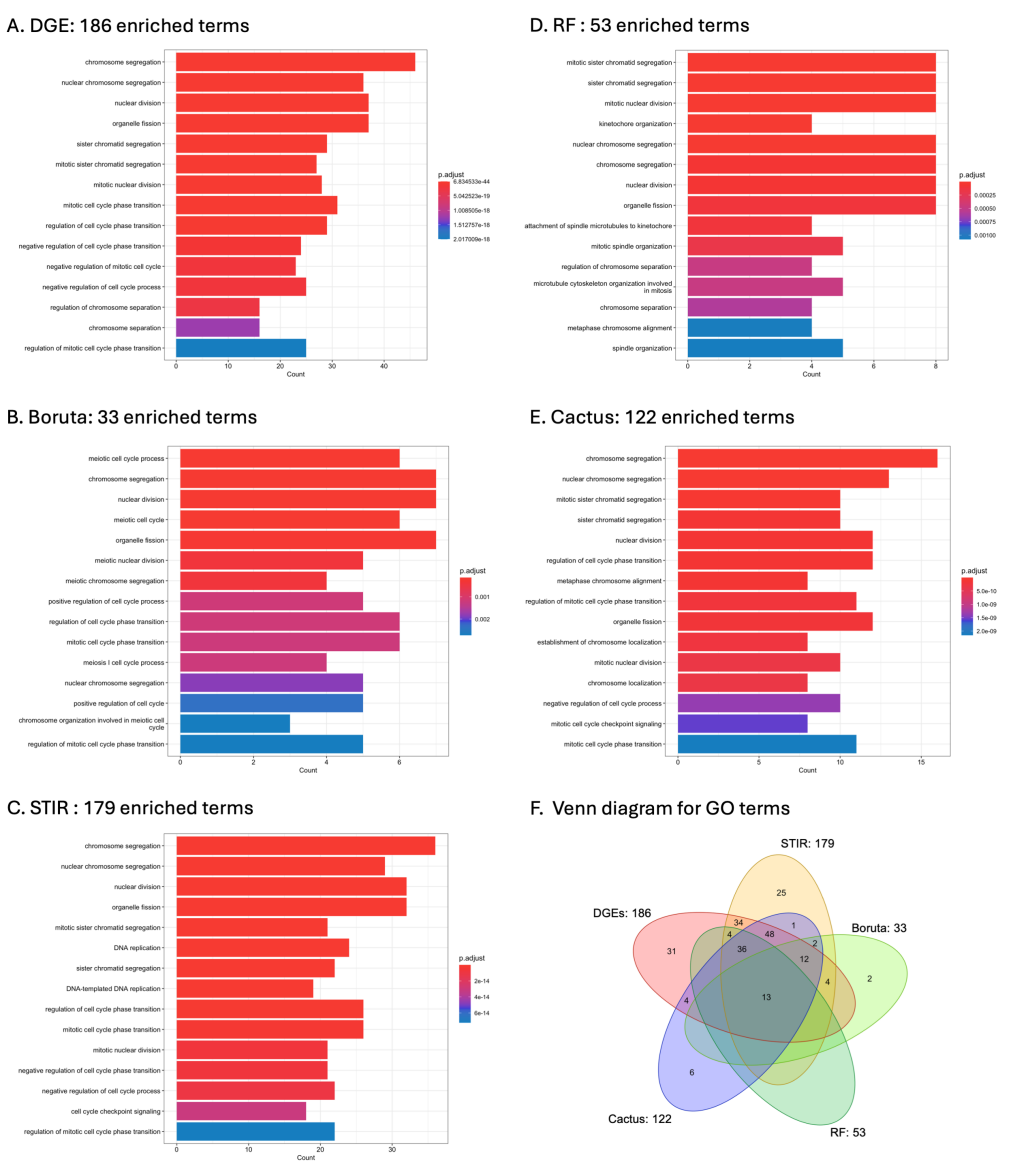
Comparison of the total number of significantly enriched GO terms identified for the methods: DGE (**A**), Boruta (**B**), STIR (**C**), RF (**D**), and CACTUS (**E**). The top 15 most significant GO terms are plotted and the number of genes identified for that GO term is indicated as the Count on the x-axis. The colour bar indicates the significance of the enrichment for that GO term as an adjusted *p*-value. (**F**) Overlap in the enriched GO terms identified by the five methods and the total number identified by each method. No enriched GO terms were found for genes selected with LASSO regression.

The highest number of enriched GO terms was found for DGE (186), followed by STIR (179), CACTUS (122), RF (53) and Boruta (33; Figure 4, Table S3). No enriched GO terms were found for genes selected with LASSO regression, even when the adjusted *p*-value was increased to 0.05. The top 15 most significant GO terms identified from each method and the number of overlapping terms are presented in (Figure 5A–F). A total of 13 GO terms were concordant between five of the methods, these included: nuclear division, mitotic spindle organisation, chromosome segregation, meiotic chromosome segregation, positive regulation of cell cycle, organelle fission, chromosome separation, kinetochore organisation, regulation of chromosome segregation, positive regulation of cell cycle process, nuclear chromosome segregation, mitotic nuclear division, and regulation of chromosome separation (Figure 5E). Many GO terms overlapped between DGE, STIR, CACTUS and RF (n = 36), as well as between DGE, STIR, and CACTUS (n = 48). No GO term was identified only by RF. For the other methods, the proportion of genes identified by only one method was as follows: 16% for DGE, 14% for STIR, 6% for Boruta and 5% for CACTUS (see Figure 5E).

#### 3.5.2. KEGG Pathway Enrichment Analyses

**Figure 6:**
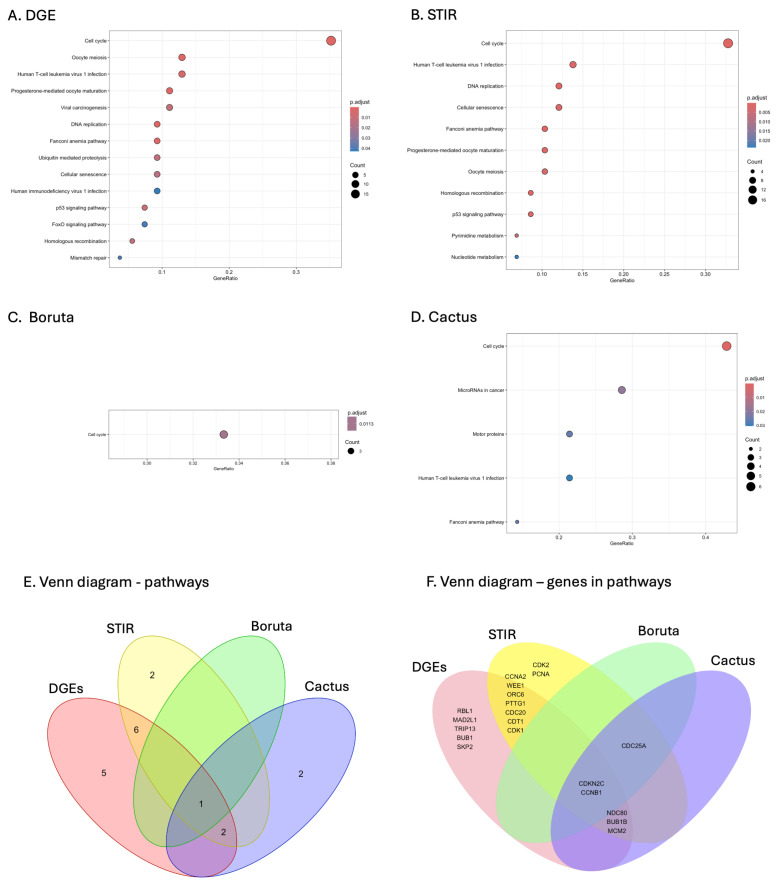
Comparison of the significant KEGG pathways identified from the signature genes of the models DGE (**A**), STIR (**B**), Boruta (**C**), and CACTUS (**D**). Overlap in the KEGG pathways identified from the signature gene sets of all models (**E**). Overlap in the genes identified for the cell cycle pathway (**F**).

No KEGG pathways were significantly enriched in the selected gene sets of RF and LASSO regression (Figure 6, Table S4). Only one KEGG pathway (cell cycle) was significantly enriched for the Boruta signature gene set. Five enriched pathways were found for the CACTUS signature gene set, with cell cycle being the most significantly enriched. A much higher number of pathways were enriched for DGE (n = 14) and STIR (n = 11), with the cell cycle also being the most significantly enriched pathway.

**Figure 7:**
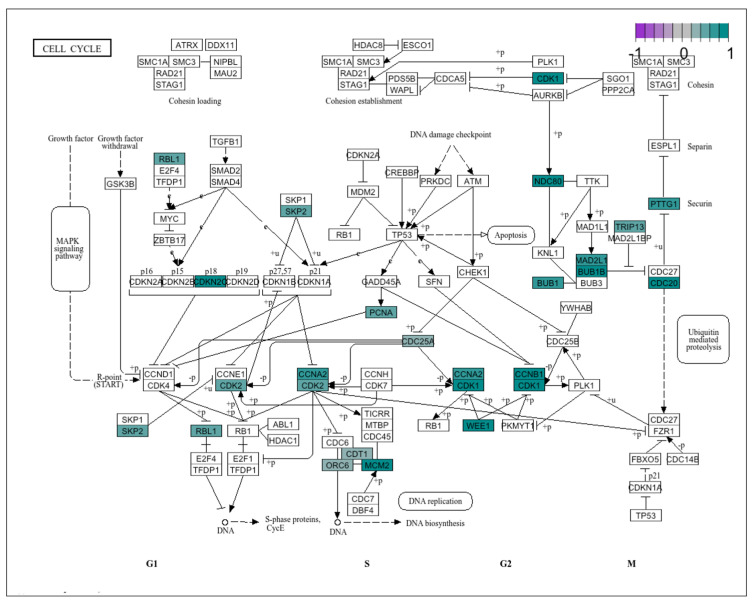
The cell cycle pathway visualisation. Genes marked in green belong to the any of four signature genes with significant enrichment of cell cycle pathway, colour gradient indicates the genes $log_{2}FC$.

Two KEGG pathways, Human T-cell leukaemia virus 1 infection and Fanconi anaemia pathway, were identified by DGE, STIR and CACTUS. For all methods, the most significantly enriched KEGG pathway identified by each of the four methods was the cell cycle pathway and all twenty genes identified were upregulated in grade 3 astrocytoma compared to grade 2 (Figure 7). Two genes: CDKN2C and CCNB1 were selected by all of the tested algorithms (Figure 6F). Seventeen genes were indicated by DGE signature genes selection, fifteen were indicated by STIR, six were selected only by CACTUS and three for the Boruta algorithm.

In the signatures datasets comparing grade 2 and grade 3 astrocytoma, the stimulation of the cell cycle pathway is visible as most of the genes were up regulated (Figure 7). Three main groups of genes are distinguishable: cycline genes (CCNA2,CCNB2, CCNB1), cycline-dependent kinases (CDK1, CDKN2C) and mitotic checkpoint serine/threonine-protein kinase (BUB1 and BUB1B). From the twenty genes enriched in this pathway four of them were indicated in the KEGG database as possible drug targets for dinaciclib (CDK1, CDK2), adavosertib (WEE1), alvocidib (CDK1, CDK2), zotiraciclib and zotiraciclib citrate (CDK1, CDK2) and tagtociclib (CDK2). The possible drug targets were indicated by the DGE or STIR signature gene sets.

### 3.6. Signature Genes and Their Prognostic Value as Biomarkers

**Figure 8:**
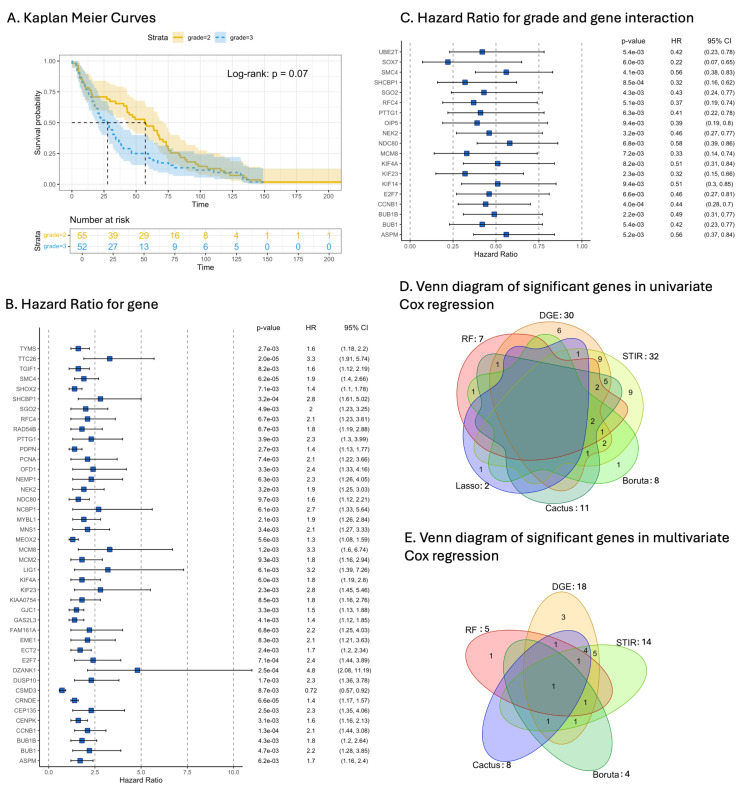
Results of the survival analysis comparing grade 2 and grade 3 astrocytoma where time estimated is in months (**A**). Results of the univariate Cox proportional hazard analysis of selected gene sets. The forest plot displays the hazard ratio and 95% confidence interval (CI) of months of survival after diagnosis as a function of gene (**B**). Results of the multivariate Cox proportional hazards model examining astrocytoma grade and gene interaction (**C**). Overlap between the genes identified to be significant for modifying the HR from the univariate (**D**) and multivariate (**E**) Cox model analysis of the signature gene sets.

There was no statistically significant difference in survival between grade 2 and grade 3 astrocytoma (Figure 8A). The prognostic value of the selected signature gene sets was assessed using Cox regression. Univariate Cox proportional hazard analysis revealed that 42 genes significantly influenced HR (*p*-value = 0.01) (Figure 8B). Of the 42 genes, only one (CSMD3) reduced HR (0.72) indicating improved survival associated with this gene whilst the rest decreased survival. The analysis showed that the gene DZANK1 has the highest influence on the increase in HR (4.8), although the 95%CI was very wide 95%CI (2.08–11.19). DZANK1 was followed by MCM8 and TTC26 both with 3.30 HR.

Multivariate Cox regression showed that the interaction between grade and gene significantly modified the HR for 19 genes. Surprisingly, the interaction between grade 3 and gene decreased the HR for all significant genes (Figure 8B,C). For univariate Cox regression, all six signature gene sets selected at least two genes that significantly influenced HR (Figure 8D). Many genes overlapped between methods, with the CACTUS signature gene set being the only set that did not contain genes that did not overlap between other methods. For multivariate Cox regression, five signature gene sets contained genes significantly modifying HR, the only exception being the LASSO regression method where no single gene was statistically significant. Of the selected genes, only CCNB1 was selected by all five methods. The CACTUS, Boruta and STIR gene sets all had genes that overlapped with other methods(Figure 8E). The full list of genes significantly modifying HR and the corresponding signature gene sets are presented in the Supplementary Materials Table S4.

## 4. Discussion

Brain tumours, and astrocytomas, in particular, are the subject of intense research to better understand their physiology and to develop new treatment strategies such as targeted therapies, immunotherapies and viral therapies [38,39,40]. Despite clinical trials, new treatments have shown little to no success in improving patient survival. Previously, microarrays have been one of the most important tools in cancer research for gene expression analysis, subclass identification and prognosis [41]. With their many limitations, they have been superseded by more advanced technologies such as next and third generation sequencing [42]. Nevertheless, methods for analysing and interpreting microarray experiments are still being developed, as relatively large amounts of data are still available in open repositories such as Array Express [43] and Gene Expression Omnibus [44]. With the growing collections of biological databases such as DrugBank, STRING, etc., and the evolution of computational methods, the re-analysis of old microarray data could provide new insights into the molecular mechanisms of disease development.

Machine learning-based frameworks have enhanced the discovery of biological patterns by enabling their multi-level interpretation. This has improved our understanding of systems biology for disease diagnosis and patient management. With a large number of features and a relatively small number of samples, determining the most relevant features and reducing the dimensionality of the data is an indispensable part of any data analysis pipeline. As the number and relevance of the features selected varies greatly between different algorithms, feature selection is a critical step that affects classification accuracy and the biological interpretation of the obtained results [10].

To further investigate the challenges associated with the feature selection step, this study applied six different feature extraction algorithms to grade 2 and 3 astrocytoma samples from the REMBRANDT cohort [17]. Results were compared considering the number of features selected, their impact on the accuracy of the tumour grade classification and their functional interpretation. The sets of signature genes and the number of genes in the set varied considerably between feature selection methods. Two methods (DGE, STIR) selected a significantly higher number of genes (113 and 128, respectively) compared with the other methods tested (Boruta: 28, LASSO: 42, RF: 37, CACTUS: 30). There was also a large difference in the genes selected, with not a single gene being selected by all of the methods tested (Figure 2). The difference in the number of selected features, as well as the difference in the number of overlapping features between the methods, is a result of the different computational approaches used in these methods.

The first three methods are based on traditional statistical approaches [25,27,45]. DGE implemented empirical Bayesian modelling, which aims to reduce the number of hyperparameters (features) in order to obtain the best estimate of the distribution of the data. The final decision is made by applying significance thresholds based on calculated moderated t-statistics. STIR provides a similar approach, it is an extension of the traditional RelieF algorithm of sample variance calculation and t-statistic-based feature elimination. The CACTUS algorithm is also inspired by traditional statistics at the level of feature selection. According to z-scoring, it allows the selection of the features that, when considered individually, provide the best possible classification. The user is able to indicate the level of confidence in the final classification based on a single feature. All of the above three methods are very sensitive to the difference in the values of the features between the two analysed classes and the difference in the number of features is caused by the significance threshold application.

RF and developed on top of it the Boruta algorithm aims to create decision boundaries in the dataset by applying gini impurity, reducing the number of selected features to the number that gives the highest possible gini impurity value [26]. LASSO regression performs an automatic feature selection based on the cost function calculation. The presence of two linearly correlated features increases the value of the cost function. In order to select the most informative feature for the regression, LASSO will try to reduce the coefficient of the less important feature to 0. This means that using these methods for feature selection can give good prediction values, with low numbers of genes needed for classification. However, in the case of co-regulated genes, perhaps only one of these genes will be selected, which could lead to the misinterpretation of biological processes. This was evident from the gene set enrichment analyses for GO and KEGG pathways, where no terms and pathways were selected. The more traditional statistical-based methods provided more informative results compared with RF, Boruta and LASSO regression selection.

Furthermore, as shown in subsequent analyses, the method’s suitability for two different tasks, tumour grade classification and functional analysis can differ. The influence of feature selection on the classification process was evaluated for two types of algorithms: mixed and single. Mixed algorithms comprised those that had both classification and selection capabilities, while single algorithms implemented classification based on pre-selected signature gene sets. Based on our analysis, pre-selection followed by classification gives much better results compared to the mixed type algorithms as well as the analysis of the whole datasets without pre-selection of features (Table 1). The pre-selection of features based on the specific algorithms allows the elimination of redundant information (correlated features) and removes noise in the data that could have a negative effect on the final classification calculation [46]. It can be seen that simple algorithms such as logistic regression are not powerful enough for the complex problem of multi-feature classification, even with the pre-selected set of features. This demonstrates how important a feature selection step is for further classification, but also how influential the choice of model is. Furthermore, even mixed models such as LASSO regression and RF suffer from the ‘curse of dimensionality’ when analysing high-dimensional data. The only exception was the CACTUS algorithm, which was able to select genes with the highest classification accuracy of all mixed methods tested (RF, LASSO).

The tested methods differ not only in the number of genes that were selected but also in their identity. There was no overlap between the methods even for a single gene, hence our analysis focused on genes selected by at least five different methods. These included CCNB1(cyclin B1), CDKN2C (cyclin dependent kinase inhibitor 2C), and GJC1 (gap junction protein gamma 1). A role for these genes in tumourigenesis has previously been suggested for astrocytoma [47]. CCNB1 is a regulatory protein that is essential for the control of the cell cycle at the G2/M (mitosis) transition. Several central nervous system cancers are associated with altered levels of the G2/M phase cyclin B1 encoded by the CCNB1 gene [48]. In histological evaluation, it has been shown that cyclin A and B protein expression are related to the grade of malignancy in astrocytoma. For grade 4 glioblastoma brain cancer, CCNB1 was indicated as a part of hub genes (with CDC6, KIF23, and KIF20A) and its expression correlated with prognosis, suggesting it as a potential biomarker for diagnosis and as a target for treatment [49]. Moreover, CCNB1, MAPK7, CD44, and CDC42 oncogenes have been associated with patients’ clinical outcomes in glioblastoma and it has been identified in simulation studies for druggable candidates of SJ10 [50].

CDKN2C has previously been described as a candidate tumour suppressor gene, but it has been observed in many types of cancer, including leukaemia [51] and prostate cancer [52], as well as malignant meningiomas and oligodendrogliomas [53,54]. The protein encoded by this gene is a member of the INK4 family of cyclin-dependent kinase inhibitors. It has been shown that CDKN2C expression is significantly increased in glioblastoma compared to non-neoplastic white matter [55]. Furthermore, its overexpression has been indicated as a potential prognostic biomarker for astrocytoma progression from grade 2 to grade 3 [56].

The GJC1 gene is a member of the connexin gene family and its protein is a component of gap junctions that provide a route for the diffusion of low molecular weight materials from cell to cell. The GJC1 protein plays a critical role in regulating cell growth and differentiation and maintaining tissue homeostasis. Although there is no evidence in the available literature of GJC1 involvement in tumourgenesis in central nervous system-related cancers, there is some evidence of its involvement in the development of liver (HG-mediated liver cancer) [57,58] or colorectal cancer [59].

It appears that the set of signature genes selected by DGE, STIR and CACTUS provides greater insight into biological phenomena and better explains differences between cancer stages than the sets of signature genes selected by RF and Boruta and LASSO. A higher number of selected genes does not give a higher probability of finding an enriched GO term. This was reflected in the analysis based on CACTUS signature genes, where a low number of features (n = 30) resulted in the selection of a high number of GO terms (n = 122) with high significance. It is important to mention that no single enriched GO term was selected by LASSO regression. Since the aim of LASSO regression is to eliminate correlated features, perhaps genes that are co-expressed were excluded thereby leading to fewer pathways being identified. This would hinder the interpretation of biological results from LASSO. Similarly, with RF and Boruta, which are tree-based algorithms, the correction of impurity could also eliminate co-expressed genes, leading to a reduction in the number of genes in a particular pathway.

Additionally, GO terms selected for DGE, STIR and CACTUS, focused on cellular processes including chromosome segregation, nuclear chromosome segregation, and nuclear division. The disruption of the cell cycle and cell division has previously been linked to the transformation of astrocytoma from grade 2 to grade 3 [60,61]. In the case of RF and Boruta signature gene sets, the enriched GO terms identified were more specific but less characteristic for brain cancer and astrocytoma. Terms identified included meiotic spindle assembly checkpoint signalling or meiotic cell cycle processes, for example.

Trends were even more evident when analysing the KEGG pathways. For a small number of non-correlated gene selections (RF and LASSO), not a single KEGG pathway was enriched. For the Boruta selection, only one pathway was enriched, which was the cell cycle pathway. More pathways were enriched for DGE, STIR and CACTUS, with the cell cycle pathway being the only pathway significantly enriched for all algorithms. It has been shown that astrocytoma development is associated with changes in the cell cycle pathway, but other pathways are also affected [62]. In addition, some of the other enriched pathways have been previously described in the brain cancer studies [63,64].

The prognostic value of the selected signature genes was assessed. Analysis revealed that the gene itself (independent of tumour grade) increased HR more than when the interaction with tumour grade was included in the analysis. From the 230 selected unique genes, 41 increased the HR and decreased survival. Only one gene (CSMD3) decreased HR and increased survival. Interestingly, multivariate Cox regression indicated that for all statistically significant gene-grade 3 interactions the HR was decreased. Some of the selected genes have previously been described as survival modifiers associated with poor prognosis in patients with different types of gliomas, including: SMC4 [65], CCNB1 [66], RFC2 [67], SHOX2 [68], KIF23 [69], NEK2 [70], CRNDE [71], TGIF1 [72], GAS2L3 [73]. The signature gene sets contained different numbers of genes significant for HR change. The number was generally proportional to the total number of genes in the signature gene sets, with two exceptions. The CACTUS signature gene set contained a much higher proportion of significant genes (36.7%) for the genes alone and 26.7% for the interaction. For the LASSO regression signature genes, only 0.05% of the total number of genes were significant in influencing HR for genes alone, and no single gene was significant when the interaction with grade was considered. This result may also be related to the mathematical basis of LASSO regression to eliminate correlated features that have a strong influence on the subsequent biological analysis.

The selection of the most important features influences the subsequent analytical steps. In this study, we have shown that different feature selections yield different sets of signature genes. The type of genes in the signature set influences the classification process and gives different accuracy results as well as insight into the biological processes. Having more features increases the complexity of the classification algorithm, without evidence that this will represent a better classification accuracy. This could be related to the impact that redundant features can have by adding noise, which is reduced when implementing RF, Boruta and LASSO regression. On the other hand, for the following biological interpretation tasks, algorithms based on traditional statistical approaches appear to work better.

## 5. Conclusions

This study demonstrates the critical importance of selecting and applying an appropriate feature selection method. It is crucial to have a comprehensive understanding of the task for which the algorithm has been designed and its mathematical foundation in order to select the most appropriate approach for the given research question. The analysis demonstrated that the utilisation of classification-type algorithms may result in the inadvertent omission of biological phenomena, while simultaneously achieving enhanced classification outcomes. As algorithms resulted in highly different signature gene sets, it is of paramount importance to subject the derived results to rigorous evaluation through the expertise knowledge or comprehensive literature searches to ensure the rational design of subsequent experiments, founded upon a robust foundation of established knowledge. Lastly, the genes and pathways identified to be concordant between models should provide greater insights into the biological mechanisms underlying disease progression in astrocytoma. Additionally, they may prove useful as biomarkers for early detection diagnostics or as targets for future therapies for precision medicine.

## Figures and Tables

**Table 1:** Signature genes commonly selected by four and five different algorithms and their mean expression ($\bar{x}$) in grade 2 *vs* grade 3 astrocytoma. Log fold change ($Log_{2}FC$) in expression between grades and the gene’s adjusted *p*-values from DGE results are also presented.

Gene	Description	${\mathrm{Log}}_{2}\mathrm{FC}$	${\bar{x}}_{\mathrm{Grade}2}$	${\bar{x}}_{\mathrm{Grade}3}$	Adjusted *p*-Value	Selection Algorithm					
*ACYP1*	Acylphosphatase 1	0.47	7.38	7.85	$3.40\times 10^{-3}$	DGE	Boruta	STIR			CACTUS
*CCNB1*	Cyclin B1	0.91	6.69	7.60	$2.69\times 10^{-3}$	DGE	Boruta	STIR		RF	CACTUS
*CDKN2C*	Cyclin Dependent Kinase Inhibitor 2C	1.19	8.72	9.91	$2.28\times 10^{-3}$	DGE	Boruta	STIR	LASSO		CACTUS
*CENPK*	Centromere Protein K	0.95	5.28	6.23	$4.20\times 10^{-3}$	DGE		STIR		RF	CACTUS
*DEPDC1*	DEP Domain Containing 1	0.43	5.98	6.41	$1.33\times 10^{-3}$		Boruta	STIR		RF	CACTUS
*FANCD2*	FA Complementation Group D2	0.50	5.56	6.06	$6.39\times 10^{-3}$	DGE	Boruta	STIR			CACTUS
*GINS1*	GINS Complex Subunit 1	0.75	7.25	8.00	$3.46\times 10^{-3}$	DGE	Boruta	STIR			CACTUS
*GJC1*	Gap Junction Protein Gamma 1	0.97	7.29	8.26	$4.07\times 10^{-3}$	DGE	Boruta	STIR		RF	CACTUS
*ITGB3BP*	Integrin Subunit Beta 3 Binding Protein	0.57	7.94	8.52	$4.12\times 10^{-3}$	DGE	Boruta	STIR			CACTUS
*MCM8*	Minichromosome Maintenance 8 Homologous	0.60	6.13	6.73	$2.28\times 10^{-3}$	DGE	Boruta	STIR			CACTUS
*NDC80*	NDC80 Kinetochore Complex Component	1.00	5.25	6.24	$4.28\times 10^{-3}$	DGE		STIR		RF	CACTUS
*NUF2*	NUF2 Component Of NDC80 Kinetochore Complex	1.05	5.16	6.21	$3.47\times 10^{-3}$	DGE	Boruta	STIR		RF	
*PIK3IP1*	Phosphoinositide-3-Kinase Interacting Protein1	−0.32	10.1	9.82	$6.39\times 10^{-3}$	DGE	Boruta	STIR		RF	
*PLK4*	Polo Like Kinase 4	0.30	7.83	8.13	$5.21\times 10^{-3}$	DGE	Boruta	STIR		RF	
*SMC4*	Structural Maintenance Of Chromosomes 4	1.10	7.67	8.78	$1.66\times 10^{-3}$	DGE	Boruta	STIR		RF	

**Table 2:** Comparison of model performance for feature selection and classification with DGE, STIR, Boruta, Random Forest, LASSO regression and CACTUS. Metrics for Sensitivity, Specificity and Balanced Accuracy are presented. Selected gene sets for DGE, STIR and Boruta were analysed using three classification models (logistic regression, KNN, SVM). Comparative results are also provided for analysis of the whole data set (*All genes*) using the classification models without pre-selection of genes. Bold indicates the highest obtained values for balanced accuracy for each experimental branch (see Figure 1).

Classification	Selection	Sensitivity	Specificity	Balanced Accuracy
**KNN**	All genes	0.923	0.462	0.692
	DGE	0.538	0.769	0.654
	Boruta	0.923	0.769	**0.846**
	STIR	0.692	0.692	0.692
**Logistic** **regression**	All genes	0.692	0.385	0.538
	DGE	0.615	0.615	**0.615**
	Boruta	0.462	0.615	0.538
	STIR	0.462	0.615	0.538
**SVM** **radial kernel**	All genes	0.692	0.769	0.731
	DGE	0.846	0.769	**0.808**
	Boruta	0.769	0.769	0.769
	STIR	0.769	0.615	0.692
**SVM** **sigmoid kernel**	All genes	0.769	0.615	0.692
	DGE	0.769	0.615	0.692
	Boruta	0.923	0.692	**0.808**
	STIR	0.923	0.615	0.769
**Random Forest**		0.714	0.667	0.691
**LASSO Regression**		0.714	0.583	0.649
**CACTUS**		0.818	0.673	**0.746**

## Data Availability

The original data presented in the study are openly available from REMBRANDT (REpository for Molecular BRAin Neoplasia DaTa), at https://sites.google.com/georgetown.edu/g-doc/home#h.vqfmcbcbp0o (accessed on 27 March 2024) raw and processed genomics and transcriptomics data are avaliable in NCBI GEO repository under the accession number GSE108476.

## Supplementary Materials

The following supporting information can be downloaded at: https://www.mdpi.com/article/10.3390/cancers16193263/s1. Table S1. Description of the clinical characteristics of the astrocytoma Grade 2 and 3 patients from the REMBRANDT cohort included in the study (n = 107); Table S2. Signature genes selected different algorithms and their mean expression (x) in grade 2 vs. grade 3. Log fold change (log_2_ FC) in expression between grades and the gene’s adjusted *p*-values from DGE results are also presented. The last column shows the total number of methods in which the gene was selected; Table S3. Results of gene ontology (GO) analysis of bilogical pathways, results were corrected for multiple testing using Benjamini-Hochberg and an adjusted *p*-value < 0.01; Table S4. Results of KEGG analysis of bilogical pathways, results were corrected for multiple testing using Benjamini-Hochberg and an adjusted *p*-value < 0.05; Table S5. Results of univariate and mulitvariet Cox regression.

## Abbreviations

The following abbreviations are used in this manuscript:

BA	Balanced Accuracy
CACTUS	Comprehensive Abstraction and Classification Tool for Uncovering Structure
CDKN2A/B	Cyclin-Dependent Kinase Inhibitor 2A/B
GO	Gene Onthology
IDH	Isocitrate Dehydrogenase
DGE	Differential Gene Expression
KEGG	Kyoto Encyclopedia of Genes and Genomes
KNN	K-Nearest Neighbors
PCA	Principal Component Analysis
QC	Quality Control
REMBRANDT	REpository for Molecular BRAin Neoplasia DaTa
STIR	STatistical Inference RelieF
SVM	Support Vector Machines
UMAP	Uniform Manifold Approximation and Projection
WHO	World Health Organisation
