# Supplementary material for: Signature Genes Selection and Functional Analysis of Astrocytoma Phenotypes: A Comparative Study
Source: Cancers (Basel). 2024 Sep 25;16(19):3263. doi: 10.3390/cancers16193263 (PMC11476064; doi:10.3390/cancers16193263)
Supplement: Supplementary file 1 [file cancers-16-03263-s001.zip › cancers-3172527-supplementary.pdf]

Supplementary materials

1. Table S1. Description of the clinical characteristics of the Astrocytoma Grade 2 and 3 patients from the REMBRANDT cohort included in the study (n = 107).

**Characteristic**	**Level**	**Grade 2**	**Grade 3**
No of patients	N	55	52
Averrage survival	Months	58	40
Gender	Male	32	24
	Female	12	15
	Unknown	11	13
Age Range	15-19	1	1
	20-24	3	1
	25-29	4	5
	30-34	9	8
	35-39	8	5
	40-44	5	5
	45-49	3	4
	50-54	5	6
	55-59	4	2
	50-64	4	4
	65-69	3	3
	70-74	2	2
	75-79	0	1
	Unknown	4	5

Table S2. Signature genes selected different algorithms and their mean expression (x) in grade 2 vs grade 3 Astrocytoma. Log fold change ($\log_2$ FC) inexpression between grades and the gene's adjusted P-values from DGE results are also presented. The last column shows the total number of methods in which the gene was selected.

Gene	Selection alghoritm						$\log_2$FC	x_{grade2}	x_{grade3}	adj.P.Val	Selection count
CCNB1	DGE	STIR	Boruta	RF	CACTUS		0,91	6,69	7,60	2,69E-03	5
CDKN2C	DGE	STIR	Boruta		CACTUS	Lasso	1,19	8,72	9,91	2,28E-03	5
GJC1	DGE	STIR	Boruta	RF	CACTUS		0,97	7,29	8,26	4,07E-03	5
ACYP1	DGE	STIR	Boruta		CACTUS		0,47	7,38	7,85	3,40E-03	4
CENPK	DGE	STIR		RF	CACTUS		0,95	5,28	6,23	4,20E-03	4
DEPDC1		STIR	Boruta	RF	CACTUS		0,43	5,98	6,41	1,33E-02	4
FANCD2	DGE	STIR	Boruta		CACTUS		0,50	5,56	6,06	6,39E-03	4

GINS1	DGE	STIR	Boruta		CACTUS		0,75	7,25	8,00	3,46E-03	4
ITGB3BP	DGE	STIR	Boruta		CACTUS		0,57	7,94	8,52	4,12E-03	4
MCM8	DGE	STIR	Boruta		CACTUS		0,60	6,13	6,73	2,28E-03	4
NDC80	DGE	STIR		RF	CACTUS		1,00	5,25	6,24	4,28E-03	4
NUF2	DGE	STIR	Boruta	RF			1,05	5,16	6,20	3,47E-03	4
PIK3IP1	DGE	STIR	Boruta	RF			-0,32	10,14	9,82	6,39E-03	4
PLK4	DGE	STIR	Boruta	RF			0,30	7,83	8,13	5,21E-03	4
SMC4	DGE	STIR	Boruta	RF			1,10	7,67	8,78	1,66E-03	4
A2M.AS1			Boruta	RF		Lasso	-0,32	6,74	6,41	1,15E-02	3
ASPM	DGE	STIR			CACTUS		1,01	5,28	6,30	3,96E-03	3
ATAD2	DGE	STIR			CACTUS		0,61	6,30	6,91	4,07E-03	3
BRCA1	DGE	STIR			CACTUS		0,37	6,84	7,21	5,51E-03	3
BUB1B	DGE	STIR			CACTUS		0,82	7,23	8,06	5,83E-03	3
C11orf80			Boruta	RF		Lasso	0,51	5,92	6,44	1,40E-02	3
CDC25A		STIR	Boruta		CACTUS		0,35	6,96	7,30	1,84E-02	3
CENPF	DGE	STIR			CACTUS		0,95	5,73	6,68	3,27E-03	3
DTL	DGE	STIR			CACTUS		0,88	6,38	7,26	5,88E-03	3
E2F7	DGE	STIR	Boruta				0,85	4,89	5,74	2,69E-03	3
ECT2	DGE	STIR			CACTUS		0,93	6,48	7,41	3,40E-03	3
EZH2	DGE	STIR			CACTUS		0,89	6,70	7,59	3,40E-03	3
FAM161A	DGE	STIR				Lasso	0,36	7,58	7,94	7,84E-03	3
HAUS1	DGE	STIR			CACTUS		0,47	7,89	8,36	5,44E-03	3
HELLS	DGE	STIR			CACTUS		0,67	5,68	6,35	4,20E-03	3
IFI27L2		STIR		RF		Lasso	-0,24	9,24	8,99	1,74E-02	3
KIF14	DGE	STIR			CACTUS		0,77	5,02	5,79	4,20E-03	3
KIF23	DGE			RF	CACTUS		0,53	4,43	4,96	7,04E-03	3
KNTC1	DGE	STIR			CACTUS		0,48	6,49	6,97	3,27E-03	3
MCM2	DGE	STIR			CACTUS		0,60	8,49	9,09	8,35E-03	3
MYBL1	DGE	STIR	Boruta				0,68	6,63	7,31	7,45E-03	3
NEK2	DGE	STIR			CACTUS		0,65	5,22	5,87	9,08E-03	3
ODF2	DGE	STIR		RF			0,42	7,34	7,75	7,84E-03	3
PRC1	DGE	STIR		RF			0,95	7,17	8,12	3,40E-03	3
PSMC3IP	DGE	STIR	Boruta				0,44	6,57	7,01	4,07E-03	3
WDR34	DGE	STIR		RF			0,44	9,04	9,48	4,20E-03	3
BRIP1	DGE	STIR					0,51	6,35	6,86	9,31E-03	2
C18orf54	DGE	STIR					0,47	6,51	6,98	7,47E-03	2
CC2	DGE	STIR					0,73	5,99	6,73	3,40E-03	2
CCNB2	DGE	STIR					0,58	6,77	7,35	7,77E-03	2
CDC20	DGE	STIR					0,80	6,23	7,03	5,44E-03	2
CDCA7	DGE	STIR					0,80	7,82	8,62	5,88E-03	2
CDK1	DGE	STIR					0,93	6,86	7,79	6,39E-03	2
CDT1	DGE	STIR					0,38	5,64	6,02	5,79E-03	2
CENPH	DGE	STIR					0,58	6,91	7,48	6,39E-03	2
CENPN	DGE	STIR					0,35	6,22	6,57	9,08E-03	2
CENPU	DGE	STIR					0,95	7,18	8,14	6,39E-03	2
CEP135	DGE	STIR					0,47	6,90	7,37	6,39E-03	2
CRNDE	DGE	STIR					1,14	6,93	8,07	4,96E-02	2
CTPS1	DGE	STIR					0,43	7,47	7,90	6,31E-03	2
DNMT1	DGE	STIR					0,38	8,77	9,15	6,39E-03	2
DZANK1			Boruta	RF			0,34	7,60	7,94	1,26E-02	2
FANCI	DGE	STIR					0,74	6,64	7,38	3,40E-03	2
GINS2	DGE	STIR					0,49	7,55	8,04	4,07E-03	2
KIF15	DGE	STIR					0,75	5,95	6,70	5,84E-03	2
KIF2C		STIR			CACTUS		0,44	7,89	8,33	1,27E-02	2
KIF4A	DGE	STIR					0,84	6,96	7,80	3,27E-03	2
LINC01355		STIR			CACTUS		0,34	7,40	7,74	1,96E-02	2
MCM4	DGE	STIR					0,50	8,53	9,03	8,11E-03	2
MELK	DGE	STIR					0,70	7,04	7,74	6,21E-03	2
MTHFD1	DGE	STIR					0,30	9,51	9,81	6,54E-03	2
NCAPG2	DGE	STIR					0,49	7,05	7,54	9,59E-03	2
NEMP1	DGE	STIR					0,46	8,03	8,50	5,44E-03	2
NUSAP1	DGE	STIR					1,04	8,27	9,31	5,60E-03	2
ORC6	DGE	STIR					0,37	8,31	8,68	4,20E-03	2
PBK	DGE	STIR					1,19	6,08	7,27	6,11E-03	2
PCLAF	DGE	STIR					0,85	8,00	8,85	4,20E-03	2
POLA1	DGE	STIR					0,28	8,29	8,57	5,05E-03	2
PTTG1	DGE	STIR					0,61	9,45	10,06	4,51E-03	2

RAD54B	DGE	STIR					0,60	6,89	7,49	4,07E-03	2
RDGE4	DGE	STIR					0,53	8,28	8,81	3,93E-03	2
RFWD3	DGE	STIR					0,37	5,95	6,31	6,39E-03	2
RNF13				RF		Lasso	-0,24	11,21	10,97	7,36E-02	2
RRM2	DGE	STIR					1,16	6,47	7,63	6,39E-03	2
SHCBP1	DGE	STIR					0,53	4,86	5,39	7,04E-03	2
SHOX2	DGE	STIR					1,41	5,12	6,53	4,20E-03	2
SLC11A2				RF		Lasso	-0,31	9,81	9,50	1,96E-02	2
STIL	DGE	STIR					0,50	6,47	6,97	3,27E-03	2
STIM2			Boruta	RF			0,39	8,32	8,72	1,05E-02	2
TOP2A	DGE	STIR					1,40	6,78	8,18	3,47E-03	2
TPX2	DGE	STIR					0,79	7,23	8,03	3,93E-03	2
UBE2T	DGE	STIR					0,61	7,24	7,84	9,78E-03	2
WEE1	DGE	STIR					0,93	8,39	9,32	4,07E-03	2
ZNF367	DGE	STIR					0,77	6,50	7,28	4,20E-03	2
ABAT				RF			0,11	8,68	8,79	5,55E-01	1
ABLIM3				RF			-0,48	9,31	8,83	5,78E-02	1
AMFR						Lasso	-0,43	8,82	8,39	2,19E-01	1
ATG10						Lasso	0,16	6,13	6,29	1,21E-01	1
B3GALNT2	DGE						0,41	8,22	8,63	8,59E-03	1
BAALC.AS2						Lasso	0,15	7,96	8,11	2,74E-01	1
BAG1		STIR					-0,16	9,44	9,28	1,86E-01	1
BARD1		STIR					0,43	7,77	8,20	2,07E-02	1
BUB1	DGE						0,66	5,13	5,78	7,04E-03	1
C14orf132						Lasso	-0,35	9,97	9,62	1,20E-01	1
CALD1				RF			0,47	8,35	8,82	9,66E-02	1
CARS2				RF			0,18	8,47	8,64	8,43E-02	1
CDCA3		STIR					0,36	7,36	7,72	1,54E-02	1
CDCA8		STIR					0,25	7,88	8,14	2,02E-02	1
CDK2		STIR					0,46	8,22	8,68	1,34E-02	1
CENPM	DGE						0,36	6,67	7,03	9,31E-03	1
CGREF1				RF			-0,10	7,78	7,68	4,08E-01	1
CHI3L1	DGE						1,03	9,06	10,09	2,03E-01	1
CHML			Boruta				0,41	7,64	8,05	5,59E-02	1
COL4A1				RF			0,40	9,14	9,54	3,48E-01	1
COQ9						Lasso	0,24	10,09	10,32	1,07E-02	1
CSMD3		STIR					-0,55	8,02	7,47	1,36E-01	1
DEK		STIR					0,25	10,66	10,91	7,04E-02	1
DMRTA2		STIR					0,61	7,04	7,65	2,24E-02	1
DMWD						Lasso	0,26	7,89	8,15	4,95E-02	1
DTYMK		STIR					0,32	8,89	9,22	2,29E-02	1
DUSP10		STIR					0,27	8,72	8,99	1,94E-01	1
EHD3				RF			-0,31	9,90	9,59	1,39E-01	1
EIF5A						Lasso	0,55	9,29	9,85	1,96E-01	1
ELMO1						Lasso	-0,47	10,16	9,69	3,91E-02	1
EME1		STIR					0,40	6,85	7,25	1,28E-02	1
EPHA5				RF			0,27	4,97	5,24	1,45E-01	1
F5				RF			-0,64	6,29	5,65	1,84E-01	1
FAM133A		STIR					-0,48	7,50	7,02	6,00E-02	1
FBXO22						Lasso	0,44	5,63	6,07	1,37E-02	1
DGEGBP				RF			0,00	9,59	9,59	9,96E-01	1
FIBP				RF			0,17	9,96	10,13	1,59E-01	1
FOXM1		STIR					0,66	7,04	7,71	1,01E-02	1
GAB2				RF			-0,25	10,32	10,07	3,28E-02	1
GAS1	DGE						0,86	9,06	9,92	4,20E-03	1
GAS2L3		STIR					0,84	6,13	6,97	3,23E-02	1
GPATCH8		STIR					-0,20	8,45	8,26	1,41E-01	1
GPC1				RF			0,37	9,97	10,34	3,80E-02	1
GSTM3						Lasso	-0,19	10,53	10,34	4,71E-01	1
GSTM5						Lasso	0,10	8,96	9,06	5,76E-01	1
HACD3		STIR					-0,21	11,73	11,52	1,88E-01	1
HEATR1		STIR					0,24	8,32	8,56	4,07E-02	1
HIST3H2A						Lasso	0,11	9,21	9,32	5,04E-01	1
HMMR	DGE						0,52	6,00	6,52	8,11E-03	1
HS3ST3B1	DGE						1,03	5,28	6,31	2,35E-02	1
IGFBP2	DGE						1,15	8,50	9,66	4,07E-02	1
IP6K3						Lasso	-0,28	7,48	7,20	1,41E-01	1

KCB1				RF			-0,47	7,63	7,16	1,36E-01	1
KCNH8						Lasso	-0,47	7,37	6,90	7,78E-02	1
KIAA0754	DGE						0,49	6,59	7,08	8,42E-03	1
KIDGE1		STIR					0,22	8,97	9,19	2,69E-02	1
KLHDC8A	DGE						0,80	8,40	9,20	4,20E-03	1
LIG1	DGE						0,42	7,89	8,31	4,07E-03	1
LOC100506725	STIR						-0,55	9,37	8,82	4,71E-02	1
LURAP1L						Lasso	-0,29	7,26	6,97	4,73E-02	1
MAD2L1	DGE						0,79	7,51	8,30	6,68E-03	1
MAP3K1					CACTUS		0,53	9,14	9,67	2,99E-02	1
Mar.08		STIR					-0,38	9,72	9,34	5,98E-02	1
MCM3		STIR					0,36	8,99	9,35	1,09E-02	1
MCM5		STIR					0,36	8,05	8,41	7,98E-02	1
MEOX2	DGE						1,09	4,76	5,85	5,09E-02	1
METTL4		STIR					0,27	7,21	7,48	1,85E-02	1
MNS1						Lasso	0,49	6,28	6,77	5,97E-02	1
MTX3	DGE						0,31	9,09	9,40	8,22E-03	1
NCAM2		STIR					-0,28	6,53	6,25	9,14E-02	1
NCAPG	DGE						0,60	5,77	6,38	4,55E-03	1
NCBP1		STIR					0,28	7,09	7,37	7,61E-02	1
NCOA4		STIR					-0,27	12,07	11,81	1,78E-02	1
NFKBIA		STIR					-0,39	11,64	11,25	2,80E-02	1
NOS1						Lasso	-0,24	5,78	5,53	4,63E-02	1
NPIPB15						Lasso	-0,15	8,72	8,58	2,19E-01	1
NPTN.IT1						Lasso	0,18	7,36	7,55	3,13E-01	1
NSL1				RF			-0,04	7,19	7,15	7,87E-01	1
OFD1	DGE						0,45	5,78	6,23	8,11E-03	1
OIP5	DGE						0,52	6,16	6,68	8,11E-03	1
OTUD1		STIR					-0,39	9,62	9,23	2,38E-02	1
OXTR						Lasso	0,52	6,83	7,35	1,07E-01	1
PCDHB6						Lasso	-0,23	6,36	6,12	4,90E-01	1
PC		STIR					0,52	9,56	10,08	1,15E-02	1
PDPN		STIR					0,71	7,00	7,71	9,95E-02	1
PEX6						Lasso	0,10	8,09	8,18	5,92E-01	1
PIMREG		STIR					0,48	7,01	7,50	1,33E-02	1
POLN						Lasso	0,16	7,38	7,54	1,72E-01	1
POU3F3		STIR					0,31	10,82	11,13	1,88E-02	1
PPP5C	DGE						0,28	8,24	8,52	9,31E-03	1
PSTPIP1		STIR					-0,31	7,32	7,01	4,41E-02	1
PTAR1		STIR					0,26	8,34	8,59	6,77E-02	1
PXMP2		STIR					0,26	9,19	9,45	2,93E-02	1
RABGAP1L						Lasso	-0,51	7,31	6,80	3,51E-02	1
RACGAP1	DGE						0,64	8,34	8,98	5,02E-03	1
RAD51AP1		STIR					0,65	5,84	6,48	1,46E-02	1
RAP1GDS1						Lasso	-0,23	9,46	9,23	1,42E-01	1
RBL1	DGE						0,41	6,01	6,42	5,00E-03	1
RBP7						Lasso	-0,31	7,04	6,73	1,24E-01	1
RCC1			Boruta				0,25	7,60	7,85	2,39E-02	1
RHOJ	DGE						0,85	7,35	8,20	6,39E-03	1
RRP7A						Lasso	0,14	6,91	7,06	1,73E-01	1
RUVBL2	DGE						0,37	9,84	10,21	4,07E-03	1
RWDD2A		STIR					-0,05	7,05	7,00	7,12E-01	1
RYBP						Lasso	-0,25	9,53	9,28	9,08E-02	1
SCARB1			Boruta				-0,15	9,85	9,70	2,36E-01	1
SCRG1						Lasso	-0,37	7,35	6,99	1,53E-01	1
SFRP2	DGE						-1,34	9,87	8,53	3,77E-02	1
SGO2	DGE						0,62	6,30	6,91	6,68E-03	1
SKP2	DGE						0,43	8,42	8,84	8,68E-03	1
SOX7				RF			0,07	6,16	6,24	5,82E-01	1
SPDL1	DGE						0,31	7,23	7,53	6,45E-03	1
SPPL3		STIR					-0,17	8,27	8,10	1,22E-01	1
SSTR2		STIR					-0,30	7,84	7,54	4,71E-02	1
TACC1		STIR					-0,36	6,95	6,58	1,91E-02	1
TACC3	DGE						0,51	6,73	7,24	7,47E-03	1
TAF1A		STIR					0,33	6,17	6,51	3,29E-02	1
TGIF1		STIR					0,60	7,95	8,55	3,43E-02	1
THUMPD2						Lasso	0,01	8,09	8,10	9,56E-01	1

TIPIN	DGE						0,36	6,56	6,92	9,64E-03	1
TMEM237						Lasso	0,46	7,74	8,20	2,22E-02	1
TNFAIP8L3						Lasso	-0,36	6,94	6,58	7,44E-02	1
TRAF5	DGE						0,61	7,32	7,93	6,39E-03	1
TRIOBP		STIR					0,25	6,26	6,51	2,69E-02	1
TRIP13	DGE						0,46	6,77	7,23	7,04E-03	1
TTC26			Boruta				0,42	4,92	5,34	4,03E-02	1
TTC39C						Lasso	0,27	7,17	7,44	1,07E-01	1
TYMS		STIR					0,83	8,63	9,47	1,54E-02	1
UBE2C	DGE						0,68	8,13	8,80	8,68E-03	1
UBE2S	DGE						0,61	8,84	9,45	7,60E-03	1
VPS26A		STIR					-0,20	10,53	10,34	9,56E-02	1
XAF1						Lasso	0,65	8,35	9,00	5,78E-02	1
ZMYM1		STIR					0,20	6,35	6,55	1,23E-01	1
ZNF232			Boruta				0,33	8,11	8,45	2,34E-02	1
ZNF365				RF			-0,45	9,27	8,82	1,26E-01	1
ZNF432	DGE						0,48	8,10	8,58	6,21E-03	1
ZNF473			Boruta				0,24	6,98	7,22	1,11E-02	1
ZNF493						Lasso	0,21	5,48	5,69	1,62E-01	1
ZNF558	DGE						0,40	8,72	9,12	8,65E-03	1
ZNF789			Boruta				0,42	7,41	7,83	1,48E-02	1
ZWILCH	DGE						0,47	7,94	8,41	7,23E-03	1

Table S3 . Results of gene ontology (GO) analysis of bilogical pathways, results were corrected for multiple testing using Benjamini-Hochberg and an adjusted P − value < 0.01.

DGE									
	ID	Description	GeneRatio	BgRatio	pvalue	p.adjust	qvalue	geneID	Count
**GO:0007059**	GO:0007059	chromosome segregation	46/109	424/18870	4.72978030899816e-47	6.83453254650233e-44	5.58611948073256e-44	SMC4/CCNB1/KIF4A/STIL/KNTC1/CENPF/ECT2/PRC1/NUF2/TOP2A/TPX2/ASPM/PSMC3IP/ITGB3BP/CENPK/KIF14/NDC80/PTTG1/NCAPG/RACGAP1/CDC20/HAUS1/BRCA1/NUSAP1/CDT1/BUB1B/CENPH/CDK1/FANCD2/CENPU/SPDL1/SGO2/MAD2L1/TRIP13/KIF23/BUB1/ZWILCH/TACC3/CCNB2/OIP5/UBE2C/CENPN/NEK2/BRIP1/CENPM/NCAPG2	46
**GO:0098813**	GO:0098813	nuclear chromosome segregation	36/109	312/18870	2.79741390100847e-37	2.02113154347862e-34	1.65194652470079e-34	SMC4/CCNB1/KIF4A/KNTC1/CENPF/ECT2/PRC1/NUF2/TOP2A/TPX2/ASPM/PSMC3IP/CENPK/KIF14/NDC80/PTTG1/NCAPG/RACGAP1/CDC20/NUSAP1/CDT1/BUB1B/CDK1/FANCD2/SPDL1/MAD2L1/TRIP13/KIF23/BUB1/ZWILCH/TACC3/CCNB2/UBE2C/NEK2/BRIP1/NCAPG2	36
**GO:0000280**	GO:0000280	nuclear division	37/109	441/18870	3.58205490391448e-33	1.72535644538548e-30	1.41019845690949e-30	SMC4/CCNB1/KIF4A/KNTC1/CENPF/PRC1/NUF2/TOP2A/TPX2/ASPM/PSMC3IP/RAD54B/CENPK/KIF14/NDC80/PTTG1/NCAPG/RACGAP1/CDC20/NUSAP1/CDT1/BUB1B/CDK1/FANCD2/SPDL1/MAD2L1/TRIP13/KIF23/BUB1/ZWILCH/MYBL1/UBE2S/CCNB2/UBE2C/NEK2/BRIP1/NCAPG2	37
**GO:0048285**	GO:0048285	organelle fission	37/109	488/18870	1.48040189487643e-31	5.34795184524109e-29	4.37108138434566e-29	SMC4/CCNB1/KIF4A/KNTC1/CENPF/PRC1/NUF2/TOP2A/TPX2/ASPM/PSMC3IP/RAD54B/CENPK/KIF14/NDC80/PTTG1/NCAPG/RACGAP1/CDC20/NUSAP1/CDT1/BUB1B/CDK1/FANCD2/SPDL1/MAD2L1/TRIP13/KIF23/BUB1/ZWILCH/MYBL1/UBE2S/CCNB2/UBE2C/NEK2/BRIP1/NCAPG2	37
**GO:0000819**	GO:0000819	sister chromatid segregation	29/109	225/18870	2.58695605794845e-31	7.47630300747102e-29	6.11066252003823e-29	SMC4/CCNB1/KIF4A/KNTC1/CENPF/PRC1/NUF2/TOP2A/TPX2/CENPK/KIF14/NDC80/NCAPG/RACGAP1/CDC20/NUSAP1/CDT1/BUB1B/CDK1/SPDL1/MAD2L1/TRIP13/KIF23/BUB1/ZWILCH/TACC3/UBE2C/NEK2/NCAPG2	29
**GO:0000070**	GO:0000070	mitotic sister chromatid segregation	27/109	184/18870	9.97031670108332e-31	2.4011846055109e-28	1.96257812958166e-28	SMC4/CCNB1/KIF4A/KNTC1/CENPF/PRC1/NUF2/TPX2/CENPK/KIF14/NDC80/NCAPG/RACGAP1/CDC20/NUSAP1/CDT1/BUB1B/CDK1/SPDL1/MAD2L1/TRIP13/KIF23/BUB1/ZWILCH/UBE2C/NEK2/NCAPG2	27
**GO:0140014**	GO:0140014	mitotic nuclear division	28/109	274/18870	2.44975801655509e-27	5.05700047703159e-25	4.13327593169145e-25	SMC4/CCNB1/KIF4A/KNTC1/CENPF/PRC1/NUF2/TPX2/CENPK/KIF14/NDC80/NCAPG/RACGAP1/CDC20/NUSAP1/CDT1/BUB1B/CDK1/SPDL1/MAD2L1/TRIP13/KIF23/BUB1/ZWILCH/UBE2S/UBE2C/NEK2/NCAPG2	28
**GO:0044772**	GO:0044772	mitotic cell cycle phase transition	31/109	470/18870	1.87803457335596e-24	3.3921999481242e-22	2.77257209382288e-22	CDKN2C/E2F7/CCNB1/STIL/KNTC1/CENPF/EZH2/CCNA2/NUF2/WEE1/KIF14/NDC80/RBL1/CDC20/BRCA1/BUB1B/DTL/MELK/RFWD3/CDK1/RRM2/SPDL1/MAD2L1/TRIP13/BUB1/ZWILCH/TACC3/UBE2S/CCNB2/SKP2/UBE2C	31
**GO:1901987**	GO:1901987	regulation of cell cycle phase transition	29/109	456/18870	2.06447344539903e-22	3.31462680955734e-20	2.70916866168154e-20	CDKN2C/E2F7/CCNB1/STIL/KNTC1/CENPF/EZH2/NUF2/WEE1/KIF14/NDC80/RBL1/CDC20/BRCA1/CDT1/BUB1B/DTL/RFWD3/CDK1/RRM2/FANCD2/SPDL1/MAD2L1/TRIP13/BUB1/ZWILCH/UBE2C/BRIP1/TIPIN	29
**GO:1901988**	GO:1901988	negative regulation of cell cycle phase transition	24/109	277/18870	9.98650663253771e-22	1.4430502084017e-19	1.17945899386393e-19	E2F7/CCNB1/KNTC1/CENPF/EZH2/NUF2/WEE1/NDC80/RBL1/CDC20/BRCA1/CDT1/BUB1B/DTL/RFWD3/CDK1/FANCD2/SPDL1/MAD2L1/TRIP13/BUB1/ZWILCH/BRIP1/TIPIN	24
**GO:0045930**	GO:0045930	negative regulation of mitotic cell cycle	23/109	248/18870	1.62469414898995e-21	1.98912018973463e-19	1.62578244609889e-19	E2F7/CCNB1/KNTC1/CENPF/EZH2/NUF2/WEE1/GAS1/NDC80/RBL1/CDC20/BRCA1/BUB1B/DTL/RFWD3/CDK1/FANCD2/SPDL1/MAD2L1/TRIP13/BUB1/ZWILCH/TIPIN	23

**GO:0010948**	GO:0010948	negative regulation of cell cycle process	25/109	320/18870	1.65186451742668e-21	1.98912018973463e-19	1.62578244609889e-19	E2F7/CCNB1/KNTC1/CENPF/EZH2/NUF2/WEE1/NDC80/RBL1/CDC20/BRCA1/CDT1/BUB1B/DTL/RFWD3/CDK1/FANCD2/SPDL1/MAD2L1/TRIP13/BUB1/ZWILCH/NEK2/BRIP1/TIPIN	25
**GO:1905818**	GO:1905818	regulation of chromosome separation	16/109	74/18870	2.49530258966343e-21	2.77362480158743e-19	2.26698745392904e-19	SMC4/CCNB1/KNTC1/CENPF/NUF2/NDC80/NCAPG/CDC20/BUB1B/SPDL1/MAD2L1/TRIP13/BUB1/ZWILCH/UBE2C/NCAPG2	16
**GO:0051304**	GO:0051304	chromosome separation	16/109	81/18870	1.20628911349146e-20	1.2450626921394e-18	1.01763637995294e-18	SMC4/CCNB1/KNTC1/CENPF/NUF2/NDC80/NCAPG/CDC20/BUB1B/SPDL1/MAD2L1/TRIP13/BUB1/ZWILCH/UBE2C/NCAPG2	16
**GO:1901990**	GO:1901990	regulation of mitotic cell cycle phase transition	25/109	355/18870	2.0937810377567e-20	2.01700906637228e-18	1.64857706972843e-18	CDKN2C/E2F7/CCNB1/STIL/KNTC1/CENPF/EZH2/NUF2/WEE1/KIF14/NDC80/RBL1/CDC20/BRCA1/BUB1B/DTL/RFWD3/CDK1/RRM2/SPDL1/MAD2L1/TRIP13/BUB1/ZWILCH/UBE2C	25
**GO:0045786**	GO:0045786	negative regulation of cell cycle	26/109	407/18870	3.81056157139717e-20	3.44141341916807e-18	2.81279610730765e-18	E2F7/CCNB1/KNTC1/CENPF/EZH2/NUF2/WEE1/GAS1/NDC80/RBL1/CDC20/BRCA1/CDT1/BUB1B/DTL/RFWD3/CDK1/FANCD2/SPDL1/MAD2L1/TRIP13/BUB1/ZWILCH/NEK2/BRIP1/TIPIN	26
**GO:0051983**	GO:0051983	regulation of chromosome segregation	18/109	131/18870	4.12907149833146e-20	3.50971077358174e-18	2.86861809357764e-18	SMC4/CCNB1/KNTC1/CENPF/NUF2/NDC80/NCAPG/CDC20/BUB1B/CDK1/SPDL1/MAD2L1/TRIP13/BUB1/ZWILCH/TACC3/UBE2C/NCAPG2	18
**GO:0000075**	GO:0000075	cell cycle checkpoint signaling	20/109	192/18870	8.46736970654386e-20	6.79741623664216e-18	5.55578293025861e-18	CCNB1/KNTC1/CENPF/NUF2/NDC80/CDC20/BRCA1/CDT1/BUB1B/DTL/RFWD3/CDK1/FANCD2/SPDL1/MAD2L1/TRIP13/BUB1/ZWILCH/BRIP1/TIPIN	20
**GO:1901991**	GO:1901991	negative regulation of mitotic cell cycle phase transition	20/109	196/18870	1.28341865668165e-19	9.76073662581567e-18	7.97781569416513e-18	E2F7/CCNB1/KNTC1/CENPF/EZH2/NUF2/WEE1/NDC80/RBL1/CDC20/BRCA1/BUB1B/DTL/RFWD3/CDK1/SPDL1/MAD2L1/TRIP13/BUB1/ZWILCH	20
**GO:0007093**	GO:0007093	mitotic cell cycle checkpoint signaling	18/109	142/18870	1.85004943163765e-19	1.33666071435821e-17	1.09250287489339e-17	CCNB1/KNTC1/CENPF/NUF2/NDC80/CDC20/BRCA1/BUB1B/DTL/RFWD3/CDK1/FANCD2/SPDL1/MAD2L1/TRIP13/BUB1/ZWILCH/TIPIN	18
**GO:0033044**	GO:0033044	regulation of chromosome organization	21/109	247/18870	6.64983718085367e-19	4.57572129825407e-17	3.73990842953274e-17	SMC4/CCNB1/KNTC1/CENPF/NUF2/TOP2A/RUVBL2/NDC80/NCAPG/CDC20/BUB1B/CDK1/SPDL1/MAD2L1/TRIP13/BUB1/ZWILCH/TACC3/UBE2C/NEK2/NCAPG2	21
**GO:0090068**	GO:0090068	positive regulation of cell cycle process	21/109	262/18870	2.25670212694995e-18	1.48224298792849e-16	1.21149272078366e-16	SMC4/E2F7/CCNB1/STIL/EZH2/ECT2/KIF14/NDC80/NCAPG/RACGAP1/PLK4/CDC20/NUSAP1/DTL/CDK1/RRM2/MAD2L1/KIF23/BUB1/UBE2C/NCAPG2	21
**GO:0033047**	GO:0033047	regulation of mitotic sister chromatid segregation	13/109	54/18870	3.46822616969512e-18	2.1789507892215e-16	1.78093810636061e-16	CCNB1/KNTC1/CENPF/NUF2/NDC80/CDC20/BUB1B/CDK1/SPDL1/MAD2L1/TRIP13/BUB1/ZWILCH	13
**GO:0010965**	GO:0010965	regulation of mitotic sister chromatid separation	13/109	59/18870	1.23700559132539e-17	7.44780449777159e-16	6.08736962046966e-16	CCNB1/KNTC1/CENPF/NUF2/NDC80/CDC20/BUB1B/SPDL1/MAD2L1/TRIP13/BUB1/ZWILCH/UBE2C	13

**GO:0007094**	GO:0007094	mitotic spindle assembly checkpoint signaling	12/109	46/18870	2.44571986852185e-17	1.29192748775998e-15	1.05594073302983e-15	CCNB1/KNTC1/CENPF/NUF2/NDC80/CDC20/BUB1B/SPDL1/MAD2L1/TRIP13/BUB1/ZWILCH	12
**GO:0071173**	GO:0071173	spindle assembly checkpoint signaling	12/109	46/18870	2.44571986852185e-17	1.29192748775998e-15	1.05594073302983e-15	CCNB1/KNTC1/CENPF/NUF2/NDC80/CDC20/BUB1B/SPDL1/MAD2L1/TRIP13/BUB1/ZWILCH	12
**GO:0071174**	GO:0071174	mitotic spindle checkpoint signaling	12/109	46/18870	2.44571986852185e-17	1.29192748775998e-15	1.05594073302983e-15	CCNB1/KNTC1/CENPF/NUF2/NDC80/CDC20/BUB1B/SPDL1/MAD2L1/TRIP13/BUB1/ZWILCH	12
**GO:0051306**	GO:0051306	mitotic sister chromatid separation	13/109	62/18870	2.50338890361795e-17	1.29192748775998e-15	1.05594073302983e-15	CCNB1/KNTC1/CENPF/NUF2/NDC80/CDC20/BUB1B/SPDL1/MAD2L1/TRIP13/BUB1/ZWILCH/UBE2C	13
**GO:0033045**	GO:0033045	regulation of sister chromatid segregation	15/109	104/18870	3.06382686340206e-17	1.52663097159172e-15	1.2477726826632e-15	CCNB1/KNTC1/CENPF/NUF2/NDC80/CDC20/BUB1B/CDK1/SPDL1/MAD2L1/TRIP13/BUB1/ZWILCH/TACC3/UBE2C	15
**GO:0031577**	GO:0031577	spindle checkpoint signaling	12/109	47/18870	3.26864777765333e-17	1.57439867956969e-15	1.28681501983405e-15	CCNB1/KNTC1/CENPF/NUF2/NDC80/CDC20/BUB1B/SPDL1/MAD2L1/TRIP13/BUB1/ZWILCH	12
**GO:0033046**	GO:0033046	negative regulation of sister chromatid segregation	12/109	48/18870	4.3374893208609e-17	1.84343296136588e-15	1.5067068167201e-15	CCNB1/KNTC1/CENPF/NUF2/NDC80/CDC20/BUB1B/SPDL1/MAD2L1/TRIP13/BUB1/ZWILCH	12
**GO:0033048**	GO:0033048	negative regulation of mitotic sister chromatid segregation	12/109	48/18870	4.3374893208609e-17	1.84343296136588e-15	1.5067068167201e-15	CCNB1/KNTC1/CENPF/NUF2/NDC80/CDC20/BUB1B/SPDL1/MAD2L1/TRIP13/BUB1/ZWILCH	12
**GO:0045841**	GO:0045841	negative regulation of mitotic metaphase/anaphase transition	12/109	48/18870	4.3374893208609e-17	1.84343296136588e-15	1.5067068167201e-15	CCNB1/KNTC1/CENPF/NUF2/NDC80/CDC20/BUB1B/SPDL1/MAD2L1/TRIP13/BUB1/ZWILCH	12
**GO:2000816**	GO:2000816	negative regulation of mitotic sister chromatid separation	12/109	48/18870	4.3374893208609e-17	1.84343296136588e-15	1.5067068167201e-15	CCNB1/KNTC1/CENPF/NUF2/NDC80/CDC20/BUB1B/SPDL1/MAD2L1/TRIP13/BUB1/ZWILCH	12
**GO:1902850**	GO:1902850	microtubule cytoskeleton organization	17/109	163/18870	5.84772026060235e-17	2.41427307902011e-15	1.97327582929198e-15	CCNB1/KIF4A/STIL/PRC1/NUF2/TPX2/NDC80/RACGAP1/CDC20/NUSAP1/CENPH/CDK1/SPDL1/MAD2L1/KIF23/TACC3/NEK2	17

		involved in mitosis							
**GO:0051985**	GO:0051985	negative regulation of chromosome segregation	12/109	50/18870	7.4865576303512e-17	2.84686204627829e-15	2.3268469975773e-15	CCNB1/KNTC1/CENPF/NUF2/NDC80/CDC20/BUB1B/SPDL1/MAD2L1/TRIP13/BUB1/ZWILCH	12
**GO:1902100**	GO:1902100	negative regulation of metaphase/anaphase transition of cell cycle	12/109	50/18870	7.4865576303512e-17	2.84686204627829e-15	2.3268469975773e-15	CCNB1/KNTC1/CENPF/NUF2/NDC80/CDC20/BUB1B/SPDL1/MAD2L1/TRIP13/BUB1/ZWILCH	12
**GO:1905819**	GO:1905819	negative regulation of chromosome separation	12/109	50/18870	7.4865576303512e-17	2.84686204627829e-15	2.3268469975773e-15	CCNB1/KNTC1/CENPF/NUF2/NDC80/CDC20/BUB1B/SPDL1/MAD2L1/TRIP13/BUB1/ZWILCH	12
**GO:0006260**	GO:0006260	DNA replication	20/109	278/18870	1.29997213069081e-16	4.81656340730312e-15	3.9367577075171e-15	MCM8/E2F7/CCNA2/GINS1/RFC4/RUVBL2/LIG1/GINS2/PCLAF/ORC6/POLA1/BRCA1/CDT1/DTL/RFWD3/CDK1/RRM2/MCM4/MCM2/TIPIN	20
**GO:0007088**	GO:0007088	regulation of mitotic nuclear division	15/109	118/18870	2.17518534966114e-16	7.85785707565086e-15	6.42252095347315e-15	CCNB1/KNTC1/CENPF/NUF2/NDC80/CDC20/NUSAP1/BUB1B/SPDL1/MAD2L1/TRIP13/BUB1/ZWILCH/UBE2C/NEK2	15
**GO:0007091**	GO:0007091	metaphase/anaphase transition of mitotic cell cycle	14/109	94/18870	2.31211765039015e-16	8.14880488978968e-15	6.66032350125225e-15	CCNB1/KNTC1/CENPF/NUF2/NDC80/CDC20/BUB1B/SPDL1/MAD2L1/TRIP13/BUB1/ZWILCH/TACC3/UBE2C	14
**GO:0045787**	GO:0045787	positive regulation of cell cycle	21/109	334/18870	3.20102271678616e-16	1.10130424422762e-14	9.00137215096257e-15	SMC4/E2F7/CCNB1/STIL/EZH2/ECT2/KIF14/NDC80/NCAPG/RACGAP1/PLK4/CDC20/NUSAP1/DTL/CDK1/RRM2/MAD2L1/KIF23/BUB1/UBE2C/NCAPG2	21
**GO:0045839**	GO:0045839	negative regulation of mitotic nuclear division	12/109	56/18870	3.34645610003086e-16	1.1245648987313e-14	9.19149019396482e-15	CCNB1/KNTC1/CENPF/NUF2/NDC80/CDC20/BUB1B/SPDL1/MAD2L1/TRIP13/BUB1/ZWILCH	12
**GO:0044784**	GO:0044784	metaphase/anaphase transition of cell cycle	14/109	97/18870	3.65800681511038e-16	1.20132269268966e-14	9.81886039845417e-15	CCNB1/KNTC1/CENPF/NUF2/NDC80/CDC20/BUB1B/SPDL1/MAD2L1/TRIP13/BUB1/ZWILCH/TACC3/UBE2C	14
**GO:0051784**	GO:0051784	negative regulation of nuclear division	12/109	63/18870	1.54676748901738e-15	4.96684227028914e-14	4.05958625187719e-14	CCNB1/KNTC1/CENPF/NUF2/NDC80/CDC20/BUB1B/SPDL1/MAD2L1/TRIP13/BUB1/ZWILCH	12
**GO:0030071**	GO:0030071	regulation of mitotic metaphase/anaphase transition	13/109	90/18870	4.33556898487889e-15	1.36193417025e-13	1.11315981717028e-13	CCNB1/KNTC1/CENPF/NUF2/NDC80/CDC20/BUB1B/SPDL1/MAD2L1/TRIP13/BUB1/ZWILCH/UBE2C	13

**GO:0051783**	GO:0051783	regulation of nuclear division	15/109	146/18870	5.59102673139382e-15	1.71894332486 47e-13	1.40495677326402e-13	CCNB1/KNTC1/CENPF/NUF2/NDC80/CDC20/NUSAP1/BUB1B/SPDL1/MAD2L1/TRIP13/BUB1/ZWILCH/UBE2C/NEK2	15
**GO:1902099**	GO:1902099	regulation of metaphase/anaphase transition of cell cycle	13/109	93/18870	6.75088977372364e-15	2.03229910896472e-13	1.66107419432411e-13	CCNB1/KNTC1/CENPF/NUF2/NDC80/CDC20/BUB1B/SPDL1/MAD2L1/TRIP13/BUB1/ZWILCH/UBE2C	13
**GO:2001251**	GO:2001251	negative regulation of chromosome organization	13/109	94/18870	7.79725784919157e-15	2.29939542695547e-13	1.87938202079333e-13	CCNB1/KNTC1/CENPF/NUF2/TOP2A/NDC80/CDC20/BUB1B/SPDL1/MAD2L1/TRIP13/BUB1/ZWILCH	13
**GO:0007051**	GO:0007051	spindle organization	16/109	202/18870	4.07172331353615e-14	1.17672803761195e-12	9.61783906902644e-13	CCNB1/KIF4A/STIL/PRC1/NUF2/TPX2/ASPM/NDC80/RACGAP1/CDC20/HAUS1/CENPH/KIF23/TACC3/CCNB2/NEK2	16
**GO:0051310**	GO:0051310	metaphase chromosome alignment	12/109	96/18870	3.09398323177552e-13	8.76628582336398e-12	7.16501379990121e-12	CCNB1/KNTC1/CENPF/ECT2/NUF2/KIF14/NDC80/RACGAP1/CDT1/SPDL1/ZWILCH/NEK2	12
**GO:0006261**	GO:0006261	DNA-templated DNA replication	14/109	161/18870	4.8720002823129e-13	1.35385392460426e-11	1.10655552970751e-11	MCM8/E2F7/GINS1/RFC4/LIG1/GINS2/ORC6/POLA1/BRCA1/CDT1/RFWD3/MCM4/MCM2/TIPIN	14
**GO:0007052**	GO:0007052	mitotic spindle organization	13/109	130/18870	5.70723110490409e-13	1.55602810312951e-11	1.27180005952381e-11	CCNB1/KIF4A/STIL/PRC1/NUF2/TPX2/NDC80/RACGAP1/CDC20/CENPH/KIF23/TACC3/NEK2	13
**GO:0051303**	GO:0051303	establishment of chromosome localization	12/109	108/18870	1.3044014049214e-12	3.49048153724337e-11	2.8529013183661e-11	CCNB1/KNTC1/CENPF/ECT2/NUF2/KIF14/NDC80/RACGAP1/CDT1/SPDL1/ZWILCH/NEK2	12
**GO:0140694**	GO:0140694	non-membrane-bounded organelle assembly	19/109	408/18870	2.04508926395579e-12	5.37300724802929e-11	4.39156010365243e-11	KIF4A/STIL/KNTC1/CENPF/PRC1/TPX2/ASPM/CENPK/NDC80/RACGAP1/PLK4/CDC20/HAUS1/BRCA1/CEP135/CENPH/KIF23/CCNB2/NEK2	19
**GO:0050000**	GO:0050000	chromosome localization	12/109	116/18870	3.09682810376408e-12	7.9909225177484e-11	6.53128032410395e-11	CCNB1/KNTC1/CENPF/ECT2/NUF2/KIF14/NDC80/RACGAP1/CDT1/SPDL1/ZWILCH/NEK2	12
**GO:0140013**	GO:0140013	meiotic nuclear division	14/109	194/18870	6.27387535255696e-12	1.59048243586751e-10	1.29996087637468e-10	SMC4/NUF2/TOP2A/ASPM/PSMC3IP/RAD54B/NDC80/PTTG1/CDC20/FANCD2/TRIP13/MYBL1/CCNB2/BRIP1	14
**GO:0008608**	GO:0008608	attachment of spindle microtubules to kinetochore	9/109	49/18870	8.66979285129282e-12	2.15997425346864e-10	1.76542787280409e-10	CCNB1/KNTC1/ECT2/NUF2/NDC80/RACGAP1/CDT1/ZWILCH/NEK2	9
**GO:0051321**	GO:0051321	meiotic cell cycle	16/109	287/18870	9.05960365798698e-12	2.21883513318495e-10	1.81353707480132e-10	SMC4/NUF2/TOP2A/ASPM/PSMC3IP/RAD54B/NDC80/PTTG1/CDC20/FANCD2/SGO2/TRIP13/MYBL1/CCNB2/NEK2/BRIP1	16

**GO:0051225**	GO:0051225	spindle assembly	12/109	133/18870	1.59261186775535e-11	3.83554024817748e-10	3.13493072916054e-10	KIF4A/STIL/PRC1/TPX2/ASPM/NDC80/RACGAP1/CDC20/HAUS1/KIF23/CCNB2/NEK2	12
**GO:0051383**	GO:0051383	kinetochore organization	7/109	21/18870	1.92023312921467e-11	4.54874897002493e-10	3.71786293525257e-10	SMC4/KNTC1/CENPF/NUF2/CENPK/NDC80/CENPH	7
**GO:1903046**	GO:1903046	meiotic cell cycle process	14/109	214/18870	2.35980619927796e-11	5.4998708999301e-10	4.49525051882831e-10	SMC4/NUF2/TOP2A/ASPM/PSMC3IP/RAD54B/NDC80/PTTG1/CDC20/FANCD2/TRIP13/MYBL1/CCNB2/BRIP1	14
**GO:0032508**	GO:0032508	DNA duplex unwinding	10/109	79/18870	2.80559935573979e-11	6.43506518895873e-10	5.25961984484552e-10	MCM8/GINS1/TOP2A/RFC4/RUVBL2/GINS2/RAD54B/MCM4/MCM2/BRIP1	10
**GO:0032392**	GO:0032392	DNA geometric change	10/109	85/18870	5.92127102246301e-11	1.33691197304048e-09	1.09270823802689e-09	MCM8/GINS1/TOP2A/RFC4/RUVBL2/GINS2/RAD54B/MCM4/MCM2/BRIP1	10
**GO:0044839**	GO:0044839	cell cycle G2/M phase transition	12/109	155/18870	9.69646918411766e-11	2.15559968785385e-09	1.7618523764502e-09	CCNB1/CENPF/CCNA2/WEE1/KIF14/NDC80/BRCA1/DTL/MELK/CDK1/CCNB2/SKP2	12
**GO:0045132**	GO:0045132	meiotic chromosome segregation	10/109	91/18870	1.18172548277957e-10	2.58726261002498e-09	2.11466665339503e-09	SMC4/NUF2/ASPM/PSMC3IP/NDC80/PTTG1/FANCD2/TRIP13/CCNB2/BRIP1	10
**GO:0071103**	GO:0071103	DNA conformation change	10/109	92/18870	1.31950770728091e-10	2.8458039358521e-09	2.32598216428779e-09	MCM8/GINS1/TOP2A/RFC4/RUVBL2/GINS2/RAD54B/MCM4/MCM2/BRIP1	10
**GO:0044786**	GO:0044786	cell cycle DNA replication	8/109	44/18870	1.42441205818331e-10	3.02687562363953e-09	2.47397883789732e-09	E2F7/GINS1/LIG1/POLA1/CDT1/MCM4/MCM2/TIPIN	8
**GO:0034508**	GO:0034508	centromere complex assembly	7/109	30/18870	3.22148523708835e-10	6.74644372114879e-09	5.51412118384916e-09	KNTC1/CENPF/ITGB3BP/CENPK/CENPH/OIP5/CENPN	7
**GO:0061982**	GO:0061982	meiosis I cell cycle process	11/109	136/18870	3.81001814378884e-10	7.86496602539268e-09	6.42833136440764e-09	TOP2A/PSMC3IP/RAD54B/NDC80/PTTG1/CDC20/FANCD2/TRIP13/MYBL1/CCNB2/BRIP1	11
**GO:0051988**	GO:0051988	regulation of attachment of spindle microtubules to kinetochore	6/109	21/18870	1.63470565049377e-09	3.32697135910352e-08	2.71925832446851e-08	CCNB1/KNTC1/ECT2/RACGAP1/ZWILCH/NEK2	6
**GO:0010639**	GO:0010639	negative regulation of organelle organization	15/109	363/18870	2.70392180551291e-09	5.42662084578634e-08	4.43538050553434e-08	CCNB1/KNTC1/CENPF/NUF2/TOP2A/TPX2/NDC80/CDC20/BRCA1/BUB1B/SPDL1/MAD2L1/TRIP13/BUB1/ZWILCH	15
**GO:0033260**	GO:0033260	nuclear DNA replication	7/109	40/18870	2.81364451726078e-09	5.56947442115319e-08	4.55214008416236e-08	GINS1/LIG1/POLA1/CDT1/MCM4/MCM2/TIPIN	7
**GO:0007127**	GO:0007127	meiosis I	10/109	129/18870	3.76957958503522e-09	7.36086824375121e-08	6.01631336331369e-08	TOP2A/PSMC3IP/RAD54B/NDC80/PTTG1/FANCD2/TRIP13/MYBL1/CCNB2/BRIP1	10

**GO:0007098**	GO:0007098	centrosome cycle	10/109	138/18870	7.25856785589341e-09	1.3984840735688e-07	1.14303342235964e-07	STIL/PCLAF/NDC80/PLK4/HAUS1/BRCA1/CEP135/CDK1/ODF2/NEK2	10
**GO:0000086**	GO:0000086	G2/M transition of mitotic cell cycle	10/109	140/18870	8.34261941225389e-09	1.5861954014088e-07	1.29645692251369e-07	CCNB1/CENPF/CCNA2/WEE1/KIF14/BRCA1/DTL/MELK/CDK1/SKP2	10
**GO:0006271**	GO:0006271	DNA strand elongation involved in DNA replication	5/109	15/18870	1.68137805529479e-08	3.15531336350776e-07	2.57895581413637e-07	GINS1/RFC4/LIG1/POLA1/MCM4	5
**GO:0031023**	GO:0031023	microtubule organizing center organization	10/109	154/18870	2.08634832951259e-08	3.86509402069961e-07	3.15908613456563e-07	STIL/PCLAF/NDC80/PLK4/HAUS1/BRCA1/CEP135/CDK1/ODF2/NEK2	10
**GO:1902969**	GO:1902969	mitotic DNA replication	5/109	16/18870	2.43441521328682e-08	4.45282276354361e-07	3.63945885317497e-07	GINS1/LIG1/POLA1/MCM4/MCM2	5
**GO:0000082**	GO:0000082	G1/S transition of mitotic cell cycle	12/109	252/18870	2.46822930296101e-08	4.45823917847332e-07	3.64388589200296e-07	CDKN2C/E2F7/STIL/EZH2/CCNA2/WEE1/KIF14/RBL1/RFWD3/CDK1/RRM2/SKP2	12
**GO:0006302**	GO:0006302	double-strand break repair	13/109	307/18870	2.50869717360649e-08	4.4753918714338e-07	3.65790543052174e-07	MCM8/RUVBL2/GINS2/RAD54B/POLA1/BRCA1/RFWD3/FANCD2/TRIP13/MCM4/MCM2/PPP5C/BRIP1	13
**GO:0006275**	GO:0006275	regulation of DNA replication	9/109	124/18870	4.18313471460514e-08	7.37149958854199e-07	6.02500275967519e-07	E2F7/CCNA2/RFC4/RUVBL2/CDT1/CDK1/MCM4/MCM2/TIPIN	9
**GO:0022616**	GO:0022616	DNA strand elongation	6/109	37/18870	6.49758644271013e-08	1.13120631442363e-06	9.24577297237 89e-07	GINS1/RFC4/RUVBL2/LIG1/POLA1/MCM4	6
**GO:0044843**	GO:0044843	cell cycle G1/S phase transition	12/109	279/18870	7.55269863356866e-08	1.29924399113175e-06	1.06192078532131e-06	CDKN2C/E2F7/STIL/EZH2/CCNA2/WEE1/KIF14/RBL1/RFWD3/CDK1/RRM2/SKP2	12
**GO:0006310**	GO:0006310	DNA recombination	13/109	340/18870	8.28866606857428e-08	1.40907323165763e-06	1.15168833794927e-06	MCM8/TOP2A/RUVBL2/PSMC3IP/GINS2/RAD54B/BRCA1/RFWD3/FANCD2/TRIP13/MCM4/MCM2/BRIP1	13
**GO:0035825**	GO:0035825	homologous recombination	7/109	68/18870	1.28136142032083e-07	2.15298517716697e-06	1.75971543892285e-06	TOP2A/PSMC3IP/RAD54B/BRCA1/FANCD2/TRIP13/BRIP1	7
**GO:0090307**	GO:0090307	mitotic spindle assembly	7/109	71/18870	1.73299581291658e-07	2.87836660880972e-06	2.35259685673612e-06	KIF4A/PRC1/TPX2/RACGAP1/CDC20/KIF23/NEK2	7
**GO:0030261**	GO:0030261	chromosome condensation	6/109	47/18870	2.8637898372395e-07	4.6496363087765e-06	3.80032193658513e-06	SMC4/TOP2A/NCAPG/NUSAP1/CDK1/NCAPG2	6
**GO:0090329**	GO:0090329	regulation of DNA-	6/109	47/18870	2.8637898372395e-07	4.6496363087765e-06	3.80032193658513e-06	E2F7/RFC4/CDT1/MCM4/MCM2/TIPIN	6

		templated DNA replication							
**GO:0051256**	GO:0051256	mitotic spindle midzone assembly	4/109	11/18870	3.36933499920979e-07	5.40965452650905e-06	4.42151329720863e-06	KIF4A/PRC1/RACGAP1/KIF23	4
**GO:0051984**	GO:0051984	positive regulation of chromosome segregation	5/109	27/18870	4.27740596069734e-07	6.79214462989853e-06	5.55147424858579e-06	SMC4/CCNB1/NCAPG/CDK1/NCAPG2	5
**GO:0034502**	GO:0034502	protein localization to chromosome	8/109	119/18870	4.40862202493231e-07	6.84995572691095e-06	5.59872542385291e-06	MCM8/KNTC1/EZH2/RUVBL2/BUB1B/CDK1/SPDL1/ZWILCH	8
**GO:1901989**	GO:1901989	positive regulation of cell cycle phase transition	8/109	119/18870	4.40862202493231e-07	6.84995572691095e-06	5.59872542385291e-06	CCNB1/STIL/EZH2/CDC20/DTL/CDK1/RRM2/UBE2C	8
**GO:0000022**	GO:0000022	mitotic spindle elongation	4/109	12/18870	5.03152229002892e-07	7.73462735009765e-06	6.32180068243276e-06	KIF4A/PRC1/RACGAP1/KIF23	4
**GO:0051656**	GO:0051656	establishment of organelle localization	14/109	469/18870	5.25562233355986e-07	7.99407818104632e-06	6.5338595659326e-06	CCNB1/KNTC1/CENPF/ECT2/NUF2/KIF14/NDC80/RACGAP1/NUSAP1/CDT1/SPDL1/MAD2L1/ZWILCH/NEK2	14
**GO:1905820**	GO:1905820	positive regulation of chromosome separation	5/109	29/18870	6.23456037813757e-07	9.38431223584249e-06	7.67014993889294e-06	SMC4/NCAPG/CDC20/UBE2C/NCAPG2	5
**GO:0007080**	GO:0007080	mitotic metaphase chromosome alignment	6/109	57/18870	9.23587477673324e-07	1.3758596961216e-05	1.12454167113345e-05	CCNB1/NUF2/KIF14/NDC80/CDT1/SPDL1	6
**GO:0051231**	GO:0051231	spindle elongation	4/109	14/18870	1.00845614089159e-06	1.4719385086751e-05	1.20307048387067e-05	KIF4A/PRC1/RACGAP1/KIF23	4
**GO:0051255**	GO:0051255	spindle midzone assembly	4/109	14/18870	1.00845614089159e-06	1.4719385086751e-05	1.20307048387067e-05	KIF4A/PRC1/RACGAP1/KIF23	4
**GO:0031570**	GO:0031570	DNA integrity checkpoint signaling	8/109	135/18870	1.15376613505332e-06	1.65231955925948e-05	1.35050267382199e-05	BRCA1/CDT1/DTL/RFWD3/CDK1/FANCD2/BRIP1/TIPIN	8
**GO:2000045**	GO:2000045	regulation of G1/S transition of	9/109	183/18870	1.15490848086649e-06	1.65231955925948e-05	1.35050267382199e-05	CDKN2C/E2F7/STIL/EZH2/WEE1/KIF14/RBL1/RFWD3/RRM2	9

		mitotic cell cycle							
**GO:0007131**	GO:0007131	reciprocal meiotic recombination	6/109	60/18870	1.25643975750594e-06	1.7626751937826e-05	1.4407004679833e-05	TOP2A/PSMC3IP/RAD54B/FANCD2/TRIP13/BRIP1	6
**GO:0140527**	GO:0140527	reciprocal homologous recombination	6/109	60/18870	1.25643975750594e-06	1.7626751937826e-05	1.4407004679833e-05	TOP2A/PSMC3IP/RAD54B/FANCD2/TRIP13/BRIP1	6
**GO:0034501**	GO:0034501	protein localization to kinetochore	4/109	15/18870	1.36905336283292e-06	1.8840781993272e-05	1.53992769232936e-05	KNTC1/CDK1/SPDL1/ZWILCH	4
**GO:1903083**	GO:1903083	protein localization to condensed chromosome	4/109	15/18870	1.36905336283292e-06	1.8840781993272e-05	1.53992769232936e-05	KNTC1/CDK1/SPDL1/ZWILCH	4
**GO:1901992**	GO:1901992	positive regulation of mitotic cell cycle phase transition	7/109	98/18870	1.58658797017317e-06	2.1628486951889e-05	1.76777726170238e-05	CCNB1/STIL/CDC20/DTL/CDK1/RRM2/UBE2C	7
**GO:0006270**	GO:0006270	DNA replication initiation	5/109	37/18870	2.20586382932871e-06	2.9789469470841e-05	2.43480493507802e-05	ORC6/POLA1/CDT1/MCM4/MCM2	5
**GO:0051382**	GO:0051382	kinetochore assembly	4/109	17/18870	2.36589299806458e-06	3.16547720574381e-05	2.58726310314665e-05	KNTC1/CENPF/CENPK/CENPH	4
**GO:1904666**	GO:1904666	regulation of ubiquitin protein ligase activity	4/109	18/18870	3.02834394404262e-06	4.01463944875375e-05	3.28131521508046e-05	CDC20/MAD2L1/UBE2S/UBE2C	4
**GO:2001252**	GO:2001252	positive regulation of chromosome organization	7/109	109/18870	3.24403544181548e-06	4.26148292129397e-05	3.48306963226504e-05	SMC4/RUVBL2/NCAPG/CDK1/BUB1/NEK2/NCAPG2	7
**GO:0071459**	GO:0071459	protein localization to chromosome/centromeric region	5/109	40/18870	3.28431143481734e-06	4.27552254352348e-05	3.49454474145572e-05	KNTC1/BUB1B/CDK1/SPDL1/ZWILCH	5
**GO:1902806**	GO:1902806	regulation of cell cycle G1/S phase transition	9/109	209/18870	3.4576987817276e-06	4.46104887463962e-05	3.64618236193455e-05	CDKN2C/E2F7/STIL/EZH2/WEE1/KIF14/RBL1/RFWD3/RRM2	9

**GO:0006268**	GO:0006268	DNA unwinding involved in DNA replication	4/109	21/18870	5.84448851732166e-06	7.47370434294673e-05	6.10853853417318e-05	GINS1/GINS2/MCM4/MCM2	4
**GO:1901976**	GO:1901976	regulation of cell cycle checkpoint	5/109	46/18870	6.6560155118427e-06	8.43679159176553e-05	6.89570582113344e-05	CCNB1/NDC80/BRCA1/RFWD3/MAD2L1	5
**GO:0000724**	GO:0000724	double-strand break repair via homologous recombination	8/109	175/18870	7.98657155038409e-06	0.000100353007741783	8.2022272581519e-05	MCM8/RUVBL2/GINS2/RAD54B/BRCA1/RFWD3/MCM4/MCM2	8
**GO:0000077**	GO:0000077	DNA damage checkpoint signaling	7/109	126/18870	8.48262757485401e-06	0.000105066751676242	8.58749920821295e-05	BRCA1/DTL/RFWD3/CDK1/FANCD2/BRIP1/TIPIN	7
**GO:0044773**	GO:0044773	mitotic DNA damage checkpoint signaling	6/109	83/18870	8.50713491080988e-06	0.000105066751676242	8.58749920821295e-05	BRCA1/DTL/RFWD3/CDK1/FANCD2/TIPIN	6
**GO:0045931**	GO:0045931	positive regulation of mitotic cell cycle	7/109	128/18870	9.40750225547499e-06	0.000114428705854151	9.35268679427113e-05	CCNB1/STIL/CDC20/DTL/CDK1/RRM2/UBE2C	7
**GO:0000725**	GO:0000725	recombinational repair	8/109	179/18870	9.42354048210652e-06	0.000114428705854151	9.35268679427113e-05	MCM8/RUVBL2/GINS2/RAD54B/BRCA1/RFWD3/MCM4/MCM2	8
**GO:0065004**	GO:0065004	protein-DNA complex assembly	9/109	240/18870	1.05916184107099e-05	0.000127540738362299	0.000104243823305408	KNTC1/CENPF/ITGB3BP/CENPK/CDT1/CENPH/OIP5/MCM2/CENPN	9
**GO:0044774**	GO:0044774	mitotic DNA integrity checkpoint signaling	6/109	87/18870	1.11709224508292e-05	0.00013340481769792	0.000109036755022448	BRCA1/DTL/RFWD3/CDK1/FANCD2/TIPIN	6
**GO:0051302**	GO:0051302	regulation of cell division	8/109	186/18870	1.2464124414691e-05	0.000147628358846136	0.000120662188035232	E2F7/ECT2/PRC1/ASPM/KIF14/RACGAP1/KIF23/SFRP2	8
**GO:0000910**	GO:0000910	cytokinesis	8/109	188/18870	1.34711454997972e-05	0.000158258579245585	0.00012935066538958	E2F7/KIF4A/ECT2/PRC1/KIF14/RACGAP1/NUSAP1/KIF23	8
**GO:0042770**	GO:0042770	signal transduction in response to DNA damage	8/109	189/18870	1.3999813317316e-05	0.000163142985834852	0.00013334287387121	E2F7/BRCA1/DTL/RFWD3/CDK1/FANCD2/BRIP1/TIPIN	8
**GO:0032465**	GO:0032465	regulation of cytokinesis	6/109	93/18870	1.63961540079031e-05	0.00018953954033136	0.000154917766710461	E2F7/ECT2/PRC1/KIF14/RACGAP1/KIF23	6

**GO:0071897**	GO:0071897	DNA biosynthetic process	8/109	194/18870	1.69128843370307e-05	0.000193961252912773	0.000158531798046353	CENPF/CCNA2/RFC4/LIG1/PCLAF/POLA1/DTL/NEK2	8
**GO:0031055**	GO:0031055	chromatin remodeling at centromere	3/109	10/18870	2.18444395840088e-05	0.000246603243741349	0.000201558069187976	ITGB3BP/OIP5/CENPN	3
**GO:0034080**	GO:0034080	CENP-A containing chromatin assembly	3/109	10/18870	2.18444395840088e-05	0.000246603243741349	0.000201558069187976	ITGB3BP/OIP5/CENPN	3
**GO:0051653**	GO:0051653	spindle localization	5/109	62/18870	2.91991507042959e-05	0.000327075757889206	0.000267331269606038	ASPM/NDC80/NUSAP1/SPDL1/MAD2L1	5
**GO:0085020**	GO:0085020	protein K6-linked ubiquitination	3/109	11/18870	2.99097543655417e-05	0.000332458423524675	0.000271730723871561	BRCA1/UBE2S/UBE2T	3
**GO:0010389**	GO:0010389	regulation of G2/M transition of mitotic cell cycle	6/109	104/18870	3.10061829626413e-05	0.000342014766267303	0.000279541480788136	CCNB1/CENPF/KIF14/BRCA1/DTL/CDK1	6
**GO:0045143**	GO:0045143	homologous chromosome segregation	5/109	65/18870	3.67625147939157e-05	0.000399412284791039	0.000326454622863264	PSMC3IP/PTTG1/FANCD2/TRIP13/BRIP1	5
**GO:0070192**	GO:0070192	chromosome organization involved in meiotic cell cycle	5/109	65/18870	3.67625147939157e-05	0.000399412284791039	0.000326454622863264	SMC4/PSMC3IP/FANCD2/TRIP13/BRIP1	5
**GO:0009314**	GO:0009314	response to radiation	11/109	428/18870	3.75479236852205e-05	0.000404901117351818	0.000330940851333993	ECT2/RUVBL2/RAD54B/PCLAF/BRCA1/DTL/PBK/RFWD3/FANCD2/TIPIN/SFRP2	11
**GO:0000727**	GO:0000727	double-strand break repair via break-induced replication	3/109	12/18870	3.97119550777213e-05	0.000421939522700788	0.000344866978306527	GINS2/MCM4/MCM2	3
**GO:0090306**	GO:0090306	meiotic spindle assembly	3/109	12/18870	3.97119550777213e-05	0.000421939522700788	0.000344866978306527	ASPM/NDC80/CCNB2	3
**GO:0031398**	GO:0031398	positive regulation of protein ubiquitination	6/109	110/18870	4.25587823106254e-05	0.000448886426560976	0.000366891692297516	FANCI/CDC20/BRCA1/UBE2S/SKP2/UBE2C	6
**GO:0007144**	GO:0007144	female meiosis I	3/109	13/18870	5.14084786383189e-05	0.00053060894023122	0.000433686564151833	NDC80/TRIP13/CCNB2	3

**GO:0044771**	GO:0044771	meiotic cell cycle phase transition	3/109	13/18870	5.14084786383189e-05	0.00053060894023122	0.000433686564151833	NDC80/CDC20/CCNB2	3
**GO:0060623**	GO:0060623	regulation of chromosome condensation	3/109	13/18870	5.14084786383189e-05	0.00053060894023122	0.000433686564151833	SMC4/NCAPG/NCAPG2	3
**GO:1902749**	GO:1902749	regulation of cell cycle G2/M phase transition	6/109	116/18870	5.73321708065438e-05	0.000587553097981956	0.000480229157483704	CCNB1/CENPF/KIF14/BRCA1/DTL/CDK1	6
**GO:0051177**	GO:0051177	meiotic sister chromatid cohesion	3/109	14/18870	6.51539393194354e-05	0.000663010157159043	0.000541903038668692	BUB1B/SGO2/BUB1	3
**GO:0007143**	GO:0007143	female meiotic nuclear division	4/109	38/18870	6.68345048141105e-05	0.000675355660534193	0.000551993481055811	TOP2A/NDC80/TRIP13/CCNB2	4
**GO:0051298**	GO:0051298	centrosome duplication	5/109	75/18870	7.337986051374e-05	0.000736346516960794	0.000601843592810061	STIL/NDC80/PLK4/BRCA1/CEP135	5
**GO:0051438**	GO:0051438	regulation of ubiquitin-protein transferase activity	4/109	39/18870	7.41427916064309e-05	0.00073887126807788	0.000603907166478515	CDC20/MAD2L1/UBE2S/UBE2C	4
**GO:0010457**	GO:0010457	centriole-centriole cohesion	3/109	15/18870	8.11001608085858e-05	0.000795389641080997	0.000650101749985706	CEP135/ODF2/NEK2	3
**GO:0030174**	GO:0030174	regulation of DNA-templated DNA replication initiation	3/109	15/18870	8.11001608085858e-05	0.000795389641080997	0.000650101749985706	CDT1/MCM4/MCM2	3
**GO:0036297**	GO:0036297	interstrand cross-link repair	4/109	40/18870	8.20159560699436e-05	0.000795389641080997	0.000650101749985706	MCM8/FANCI/RFWD3/FANCD2	4
**GO:0040001**	GO:0040001	establishment of mitotic spindle localization	4/109	40/18870	8.20159560699436e-05	0.000795389641080997	0.000650101749985706	NDC80/NUSAP1/SPDL1/MAD2L1	4
**GO:0007099**	GO:0007099	centriole replication	4/109	43/18870	0.000109286761781992	0.00105279580516652	0.000860489450662418	STIL/PLK4/BRCA1/CEP135	4
**GO:1903322**	GO:1903322	positive regulation of protein modification by small	6/109	132/18870	0.000117418310235851	0.00112363879662785	0.000918392081454342	FANCI/CDC20/BRCA1/UBE2S/SKP2/UBE2C	6

		protein conjugation or removal							
**GO:2000134**	GO:2000134	negative regulation of G1/S transition of mitotic cell cycle	5/109	86/18870	0.000140803586423259	0.00133856041040532	0.00109405556763779	E2F7/EZH2/WEE1/RBL1/RFWD3	5
**GO:0051347**	GO:0051347	positive regulation of transferase activity	10/109	414/18870	0.00014308670389089	0.00135137442563618	0.00110452894231564	EZH2/ECT2/TPX2/RFC4/KIF14/CDC20/UBE2S/UBE2C/NEK2/CHI3L1	10
**GO:0010212**	GO:0010212	response to ionizing radiation	6/109	138/18870	0.000149844569251813	0.00140600910759006	0.00114918391456278	ECT2/RAD54B/BRCA1/RFWD3/FANCD2/SFRP2	6
**GO:0032467**	GO:0032467	positive regulation of cytokinesis	4/109	47/18870	0.000155174724020719	0.0014466288787738	0.00118238397522069	ECT2/KIF14/RACGAP1/KIF23	4
**GO:0000281**	GO:0000281	mitotic cytokinesis	5/109	88/18870	0.000156947508874994	0.00145377660464338	0.001188226079337	KIF4A/ECT2/RACGAP1/NUSAP1/KIF23	5
**GO:0031396**	GO:0031396	regulation of protein ubiquitination	7/109	200/18870	0.000161848388699407	0.00148962370490856	0.00121752525726272	FANCI/CDC20/BRCA1/MAD2L1/UBE2S/SKP2/UBE2C	7
**GO:0098534**	GO:0098534	centriole assembly	4/109	48/18870	0.000168532557582523	0.00153380634619431	0.00125363738512476	STIL/PLK4/BRCA1/CEP135	4
**GO:0000212**	GO:0000212	meiotic spindle organization	3/109	19/18870	0.000169833228644353	0.00153380634619431	0.00125363738512476	ASPM/NDC80/CCNB2	3
**GO:0007076**	GO:0007076	mitotic chromosome condensation	3/109	19/18870	0.000169833228644353	0.00153380634619431	0.00125363738512476	SMC4/NCAPG/NUSAP1	3
**GO:1902807**	GO:1902807	negative regulation of cell cycle G1/S phase transition	5/109	95/18870	0.000224819149192845	0.00201778677381156	0.00164921271915248	E2F7/EZH2/WEE1/RBL1/RFWD3	5
**GO:0051315**	GO:0051315	attachment of mitotic spindle microtubules to kinetochore	3/109	21/18870	0.000231151922255763	0.00202433047066411	0.00165456112772546	NUF2/NDC80/CDT1	3
**GO:0090231**	GO:0090231	regulation of spindle checkpoint	3/109	21/18870	0.000231151922255763	0.00202433047066411	0.00165456112772546	CCNB1/NDC80/MAD2L1	3

**GO:0090266**	GO:0090266	regulation of mitotic cell cycle spindle assembly checkpoint	3/109	21/18870	0.000231151922255763	0.00202433047066411	0.00165456112772546	CCNB1/NDC80/MAD2L1	3
**GO:1903504**	GO:1903504	regulation of mitotic spindle checkpoint	3/109	21/18870	0.000231151922255763	0.00202433047066411	0.00165456112772546	CCNB1/NDC80/MAD2L1	3
**GO:0007129**	GO:0007129	homologous chromosome pairing at meiosis	4/109	53/18870	0.000248067310282813	0.00215938110457027	0.00176494306998932	PSMC3IP/FANCD2/TRIP13/BRIP1	4
**GO:0051443**	GO:0051443	positive regulation of ubiquitin-protein transferase activity	3/109	22/18870	0.00026652672000206	0.00230617431379028	0.00188492265894933	CDC20/UBE2S/UBE2C	3
**GO:0046599**	GO:0046599	regulation of centriole replication	3/109	23/18870	0.000305220157252547	0.00262525670970197	0.00214572065436941	STIL/PLK4/BRCA1	3
**GO:0045005**	GO:0045005	DNA-templated DNA replication maintenance of fidelity	4/109	56/18870	0.000307043220449704	0.00262531037603445	0.00214576451787336	MCM8/BRCA1/RFWD3/TIPIN	4
**GO:1901875**	GO:1901875	positive regulation of post-translational protein modification	6/109	159/18870	0.000322642279865691	0.00274245937885837	0.00224151478643533	FANCI/CDC20/BRCA1/UBE2S/SKP2/UBE2C	6
**GO:0051293**	GO:0051293	establishment of spindle localization	4/109	57/18870	0.000328757322818664	0.00277809550568988	0.00227064152787036	NDC80/NUSAP1/SPDL1/MAD2L1	4
**GO:0051054**	GO:0051054	positive regulation of DNA metabolic process	8/109	298/18870	0.000336817741291445	0.00282966067538453	0.00231278767276011	E2F7/CCNA2/RFC4/RUVBL2/BRCA1/CDT1/CDK1/NEK2	8
**GO:0000209**	GO:0000209	protein polyubiquitination	7/109	226/18870	0.000341087657601381	0.00284896916320229	0.00232856922317462	BRCA1/DTL/TRAF5/UBE2S/SKP2/UBE2C/UBE2T	7
**GO:0031145**	GO:0031145	anaphase-promoting complex-dependent	3/109	24/18870	0.00034736033687634	0.00288468785509374	0.00235776344812615	CDC20/UBE2S/UBE2C	3

		catabolic process							
**GO:0007292**	GO:0007292	female gamete generation	6/109	163/18870	0.000368484907475959	0.00304263252173006	0.00248685754098061	MCM8/TOP2A/ASPM/NDC80/TRIP13/CCNB2	6
**GO:0007062**	GO:0007062	sister chromatid cohesion	4/109	59/18870	0.000375451427933109	0.00308254155320081	0.00251947668744586	CDC20/BUB1B/SGO2/BUB1	4
**GO:0001701**	GO:0001701	in utero embryonic development	9/109	392/18870	0.000450182527182443	0.00367521893660243	0.00300389411536545	E2F7/CCNB1/STIL/GINS1/PLK4/RRM2/CCNB2/NEK2/NCAPG2	9
**GO:1903320**	GO:1903320	regulation of protein modification by small protein conjugation or removal	7/109	243/18870	0.000526638485974541	0.00427523939456861	0.00349431331320778	FANCI/CDC20/BRCA1/MAD2L1/UBE2S/SKP2/UBE2C	7
**GO:0061640**	GO:0061640	cytoskeleton-dependent cytokinesis	5/109	117/18870	0.000588053833465245	0.00474713848802949	0.00388001412024702	KIF4A/ECT2/RACGAP1/NUSAP1/KIF23	5
**GO:0010971**	GO:0010971	positive regulation of G2/M transition of mitotic cell cycle	3/109	29/18870	0.00061407356681663	0.00492964613361128	0.00402918445595473	CCNB1/DTL/CDK1	3
**GO:0070979**	GO:0070979	protein K11-linked ubiquitination	3/109	30/18870	0.000679447157258609	0.00542431570297619	0.00443349642596196	UBE2S/UBE2C/UBE2T	3
**GO:0010458**	GO:0010458	exit from mitosis	3/109	32/18870	0.000823129717967618	0.00649957618832354	0.00531234710129231	KNTC1/UBE2S/UBE2C	3
**GO:1902751**	GO:1902751	positive regulation of cell cycle G2/M phase transition	3/109	32/18870	0.000823129717967618	0.00649957618832354	0.00531234710129231	CCNB1/DTL/CDK1	3
**GO:0006301**	GO:0006301	postreplication repair	3/109	34/18870	0.000984774901553093	0.00773369419969685	0.00632103798365315	PCLAF/BRCA1/DTL	3
**GO:0000132**	GO:0000132	establishment of mitotic spindle orientation	3/109	35/18870	0.00107259338809966	0.00837782403137302	0.00684750942502315	NDC80/SPDL1/MAD2L1	3
**GO:0007095**	GO:0007095	mitotic G2 DNA damage checkpoint signaling	3/109	37/18870	0.00126272278449489	0.00980986249244684	0.00801796810528163	BRCA1/DTL/CDK1	3

STIR										
	ID	Description	GeneRatio	BgRatio	pvalue	p.adjust	qvalue	geneID	Count
**GO:0007059**	GO:0007059	chromosome segregation	36/120	424/18870	1.00573826956451e-30	1.62929599669451e-27	1.35827599984344e-27	SMC4/CENPF/TOP2A/ITGB3BP/ECT2/PSMC3IP/NUSAP1/CENPU/CCNB1/KNTC1/CENPK/ASPM/NUF2/BRCA1/BRIP1/PRC1/STIL/CENPN/NDC80/KIF14/KIF4A/HAUS1/TPX2/CDC20/PTTG1/KIF2C/BUB1B/KIFC1/CDCA8/CDT1/NEK2/CENPH/FANCD2/CCNB2/CDK1/NCAPG2	36
**GO:0098813**	GO:0098813	nuclear chromosome segregation	29/120	312/18870	8.47622789839288e-26	6.86574459769823e-23	5.72368441770424e-23	SMC4/CENPF/TOP2A/ECT2/PSMC3IP/NUSAP1/CCNB1/KNTC1/CENPK/ASPM/NUF2/BRIP1/PRC1/NDC80/KIF14/KIF4A/TPX2/CDC20/PTTG1/KIF2C/BUB1B/KIFC1/CDCA8/CDT1/NEK2/FANCD2/CCNB2/CDK1/NCAPG2	29
**GO:0000280**	GO:0000280	nuclear division	32/120	441/18870	4.2739777337032e-25	2.30794797619973e-22	1.92403980082148e-22	SMC4/RAD54B/CENPF/TOP2A/PSMC3IP/NUSAP1/CCNB1/KNTC1/CENPK/ASPM/NUF2/BRIP1/PRC1/NDC80/KIF14/KIF4A/TPX2/CDC20/PTTG1/KIF2C/BUB1B/RAD51AP1/KIFC1/CDCA8/CDT1/EME1/NEK2/FANCD2/CCNB2/CDK1/MYBL1/NCAPG2	32
**GO:0048285**	GO:0048285	organelle fission	32/120	488/18870	9.83843300174612e-24	3.98456536570718e-21	3.32176566874744e-21	SMC4/RAD54B/CENPF/TOP2A/PSMC3IP/NUSAP1/CCNB1/KNTC1/CENPK/ASPM/NUF2/BRIP1/PRC1/NDC80/KIF14/KIF4A/TPX2/CDC20/PTTG1/KIF2C/BUB1B/RAD51AP1/KIFC1/CDCA8/CDT1/EME1/NEK2/FANCD2/CCNB2/CDK1/MYBL1/NCAPG2	32
**GO:0000070**	GO:0000070	mitotic sister chromatid segregation	21/120	184/18870	1.12128370009985e-20	3.34970440993491e-18	2.79250861465009e-18	SMC4/CENPF/NUSAP1/CCNB1/KNTC1/CENPK/NUF2/PRC1/NDC80/KIF14/KIF4A/TPX2/CDC20/KIF2C/BUB1B/KIFC1/CDCA8/CDT1/NEK2/CDK1/NCAPG2	21
**GO:0006260**	GO:0006260	DNA replication	24/120	278/18870	1.24063126293885e-20	3.34970440993491e-18	2.79250861465009e-18	DTL/E2F7/PCLAF/MCM2/BRCA1/CCNA2/RFC4/RRM2/POLA1/MCM8/GINS1/MCM4/ORC6/RFWD3/CDK2/MCM3/PCNA/GINS2/METTL4/MCM5/CDT1/EME1/BARD1/CDK1	24
**GO:0000819**	GO:0000819	sister chromatid segregation	22/120	225/18870	3.84075424998259e-20	8.88860269281684e-18	7.41005669583106e-18	SMC4/CENPF/TOP2A/NUSAP1/CCNB1/KNTC1/CENPK/NUF2/PRC1/NDC80/KIF14/KIF4A/TPX2/CDC20/KIF2C/BUB1B/KIFC1/CDCA8/CDT1/NEK2/CDK1/NCAPG2	22
**GO:0006261**	GO:0006261	DNA-templated DNA replication	19/120	161/18870	4.61721674100368e-19	9.34986390053246e-17	7.79459089303648e-17	E2F7/MCM2/BRCA1/RFC4/POLA1/MCM8/GINS1/MCM4/ORC6/RFWD3/CDK2/MCM3/PCNA/GINS2/METTL4/MCM5/CDT1/EME1/BARD1	19
**GO:1901987**	GO:1901987	regulation of cell cycle phase transition	26/120	456/18870	8.52282021706888e-18	1.5341076390724e-15	1.27892144309934e-15	CDKN2C/CENPF/DTL/E2F7/WEE1/CCNB1/KNTC1/EZH2/NUF2/BRCA1/BRIP1/STIL/NDC80/KIF14/RRM2/CDC20/RFWD3/CDK2/BUB1B/CDCA8/CDT1/EME1/FANCD2/BARD1/CDC25A/CDK1	26
**GO:0044772**	GO:0044772	mitotic cell cycle phase transition	26/120	470/18870	1.78552312521412e-17	2.89254746284688e-15	2.41139596805234e-15	CDKN2C/CENPF/DTL/E2F7/WEE1/CCNB1/KNTC1/EZH2/NUF2/BRCA1/CCNA2/STIL/NDC80/KIF14/RRM2/CDC20/RFWD3/CDK2/BUB1B/FOXM1/CDCA8/BARD1/CCNB2/CDC25A/CDK1/MELK	26
**GO:0140014**	GO:0140014	mitotic nuclear division	21/120	274/18870	4.53838426365029e-17	6.68380227919407e-15	5.57200670838596e-15	SMC4/CENPF/NUSAP1/CCNB1/KNTC1/CENPK/NUF2/PRC1/NDC80/KIF14/KIF4A/TPX2/CDC20/KIF2C/BUB1B/KIFC1/CDCA8/CDT1/NEK2/CDK1/NCAPG2	21
**GO:1901988**	GO:1901988	negative regulation of cell cycle phase transition	21/120	277/18870	5.66752950969864e-17	7.65116483809317e-15	6.37845645696786e-15	CENPF/DTL/E2F7/WEE1/CCNB1/KNTC1/EZH2/NUF2/BRCA1/BRIP1/NDC80/CDC20/RFWD3/CDK2/BUB1B/CDCA8/CDT1/EME1/FANCD2/BARD1/CDK1	21
**GO:0010948**	GO:0010948	negative regulation of cell cycle process	22/120	320/18870	7.61897617106125e-17	9.49441645932248e-15	7.91509832183934e-15	CENPF/DTL/E2F7/WEE1/CCNB1/KNTC1/EZH2/NUF2/BRCA1/BRIP1/NDC80/CDC20/RFWD3/CDK2/BUB1B/CDCA8/CDT1/EME1/NEK2/FANCD2/BARD1/CDK1	22

**GO:0000075**	GO:0000075	cell cycle checkpoint signaling	18/120	192/18870	2.68248064016304e-16	3.10401331218866e-14	2.5876862115257e-14	CENPF/DTL/CCNB1/KNTC1/NUF2/BRCA1/BRIP1/NDC80/CDC20/RFWD3/CDK2/BUB1B/CDCA8/CDT1/EME1/FANCD2/BARD1/CDK1	18
**GO:1901990**	GO:1901990	regulation of mitotic cell cycle phase transition	22/120	355/18870	6.7474950801553e-16	7.20881228471746e-14	6.00968561487492e-14	CDKN2C/CENPF/DTL/E2F7/WEE1/CCNB1/KNTC1/EZH2/NUF2/BRCA1/STIL/NDC80/KIF14/RRM2/CDC20/RFWD3/CDK2/BUB1B/CDCA8/BARD1/CDC25A/CDK1	22
**GO:0007093**	GO:0007093	mitotic cell cycle checkpoint signaling	16/120	142/18870	7.11981460219009e-16	7.20881228471746e-14	6.00968561487492e-14	CENPF/DTL/CCNB1/KNTC1/NUF2/BRCA1/NDC80/CDC20/RFWD3/CDK2/BUB1B/CDCA8/EME1/FANCD2/BARD1/CDK1	16
**GO:0045930**	GO:0045930	negative regulation of mitotic cell cycle	19/120	248/18870	1.61424432109128e-15	1.53827988245169e-13	1.28239966808676e-13	CENPF/DTL/E2F7/WEE1/CCNB1/KNTC1/EZH2/NUF2/BRCA1/NDC80/CDC20/RFWD3/CDK2/BUB1B/CDCA8/EME1/FANCD2/BARD1/CDK1	19
**GO:1901991**	GO:1901991	negative regulation of mitotic cell cycle phase transition	17/120	196/18870	7.05313100390614e-15	6.34781790351553e-13	5.2919105719366e-13	CENPF/DTL/E2F7/WEE1/CCNB1/KNTC1/EZH2/NUF2/BRCA1/NDC80/CDC20/RFWD3/CDK2/BUB1B/CDCA8/BARD1/CDK1	17
**GO:0045786**	GO:0045786	negative regulation of cell cycle	22/120	407/18870	1.14411283034055e-14	9.75506729027202e-13	8.13239202951202e-13	CENPF/DTL/E2F7/WEE1/CCNB1/KNTC1/EZH2/NUF2/BRCA1/BRIP1/NDC80/CDC20/RFWD3/CDK2/BUB1B/CDCA8/CDT1/EME1/NEK2/FANCD2/BARD1/CDK1	22
**GO:0044839**	GO:0044839	cell cycle G2/M phase transition	15/120	155/18870	5.92922456066237e-14	4.80267189413652e-12	4.00378690069991e-12	CENPF/DTL/WEE1/CCNB1/BRCA1/CCNA2/NDC80/KIF14/CDK2/FOXM1/BARD1/CCNB2/CDC25A/CDK1/MELK	15
**GO:0090068**	GO:0090068	positive regulation of cell cycle process	18/120	262/18870	6.29209081659748e-14	4.85389862994663e-12	4.04649249007247e-12	SMC4/DTL/E2F7/ECT2/PLK4/NUSAP1/CCNB1/EZH2/STIL/NDC80/KIF14/RRM2/CDC20/RAD51AP1/CDCA8/CDC25A/CDK1/NCAPG2	18
**GO:1902850**	GO:1902850	microtubule cytoskeleton organization involved in mitosis	15/120	163/18870	1.25302085417636e-13	9.22678992620777e-12	7.69198926271902e-12	NUSAP1/CCNB1/NUF2/PRC1/STIL/NDC80/KIF4A/TPX2/CDC20/TACC1/KIFC1/CDCA8/NEK2/CENPH/CDK1	15
**GO:0007051**	GO:0007051	spindle organization	16/120	202/18870	1.91190553128842e-13	1.34664650464663e-11	1.12264292752542e-11	CCNB1/ASPM/NUF2/PRC1/STIL/NDC80/KIF4A/HAUS1/TPX2/CDC20/TACC1/KIFC1/CDCA8/NEK2/CENPH/CCNB2	16
**GO:0051321**	GO:0051321	meiotic cell cycle	18/120	287/18870	3.00625321763805e-13	2.02922092190568e-11	1.6916767009779e-11	SMC4/RAD54B/TOP2A/PSMC3IP/ASPM/NUF2/BRIP1/NDC80/CDC20/PTTG1/CDK2/RAD51AP1/EME1/NEK2/FANCD2/CCNB2/CDC25A/MYBL1	18
**GO:0090329**	GO:0090329	regulation of DNA-templated DNA replication	10/120	47/18870	3.13709304679666e-13	2.03283629432423e-11	1.69469068591163e-11	E2F7/MCM2/RFC4/MCM4/CDK2/MCM3/PCNA/METTL4/MCM5/CDT1	10
**GO:1903046**	GO:1903046	meiotic cell cycle process	16/120	214/18870	4.68172684599883e-13	2.91707595789158e-11	2.43184434956134e-11	SMC4/RAD54B/TOP2A/PSMC3IP/ASPM/NUF2/BRIP1/NDC80/CDC20/PTTG1/RAD51AP1/EME1/FANCD2/CCNB2/CDC25A/MYBL1	16

**GO:0051310**	GO:0051310	metaphase chromosome alignment	12/120	96/18870	9.94717158925225e-13	5.96830295355135e-11	4.9755248144291e-11	CENPF/ECT2/CCNB1/KNTC1/NUF2/NDC80/KIF14/KIF2C/KIFC1/CDCA8/CDT1/NEK2	12
**GO:0140694**	GO:0140694	non-membrane-bounded organelle assembly	20/120	408/18870	1.27627000042261e-12	7.3841335738737e-11	6.15584364865494e-11	CENPF/CEP135/PLK4/KNTC1/CENPK/ASPM/BRCA1/PRC1/STIL/NDC80/KIF4A/HAUS1/TPX2/CDC20/CDK2/KIFC1/CDCA8/NEK2/CENPH/CCNB2	20
**GO:0140013**	GO:0140013	meiotic nuclear division	15/120	194/18870	1.61687491333593e-12	9.03219779173866e-11	7.52976593034484e-11	SMC4/RAD54B/TOP2A/PSMC3IP/ASPM/NUF2/BRIP1/NDC80/CDC20/PTTG1/RAD51AP1/EME1/FANCD2/CCNB2/MYBL1	15
**GO:0007052**	GO:0007052	mitotic spindle organization	13/120	130/18870	2.0060198240008e-12	1.08325070496043e-10	9.03060854102818e-11	CCNB1/NUF2/PRC1/STIL/NDC80/KIF4A/TPX2/CDC20/TACC1/KIFC1/CDCA8/NEK2/CENPH	13
**GO:0032508**	GO:0032508	DNA duplex unwinding	11/120	79/18870	2.70851378765487e-12	1.41541688258093e-10	1.17997391835694e-10	RAD54B/TOP2A/MCM2/BRIP1/RFC4/MCM8/GINS1/MCM4/MCM3/GINS2/MCM5	11
**GO:0061982**	GO:0061982	meiosis I cell cycle process	13/120	136/18870	3.59375259135339e-12	1.81933724937265e-10	1.51670545220605e-10	RAD54B/TOP2A/PSMC3IP/BRIP1/NDC80/CDC20/PTTG1/RAD51AP1/EME1/FANCD2/CCNB2/CDC25A/MYBL1	13
**GO:0045787**	GO:0045787	positive regulation of cell cycle	18/120	334/18870	3.90372309102634e-12	1.91637315377657e-10	1.59760023151094e-10	SMC4/DTL/E2F7/ECT2/PLK4/NUSAP1/CCNB1/EZH2/STIL/NDC80/KIF14/RRM2/CDC20/RAD51AP1/CDCA8/CDC25A/CDK1/NCAPG2	18
**GO:0051303**	GO:0051303	establishment of chromosome localization	12/120	108/18870	4.16654631030917e-12	1.9852367713826e-10	1.6550089523612e-10	CENPF/ECT2/CCNB1/KNTC1/NUF2/NDC80/KIF14/KIF2C/KIFC1/CDCA8/CDT1/NEK2	12
**GO:0000086**	GO:0000086	G2/M transition of mitotic cell cycle	13/120	140/18870	5.21895095117795e-12	2.41562872597379e-10	2.01380874296581e-10	CENPF/DTL/WEE1/CCNB1/BRCA1/CCNA2/KIF14/CDK2/FOXM1/BARD1/CDC25A/CDK1/MELK	13
**GO:0032392**	GO:0032392	DNA geometric change	11/120	85/18870	6.19371234989891e-12	2.78717055745451e-10	2.32354764471354e-10	RAD54B/TOP2A/MCM2/BRIP1/RFC4/MCM8/GINS1/MCM4/MCM3/GINS2/MCM5	11
**GO:0050000**	GO:0050000	chromosome localization	12/120	116/18870	9.84928234458078e-12	4.31238848600564e-10	3.59505810756675e-10	CENPF/ECT2/CCNB1/KNTC1/NUF2/NDC80/KIF14/KIF2C/KIFC1/CDCA8/CDT1/NEK2	12
**GO:0071103**	GO:0071103	DNA conformation change	11/120	92/18870	1.5034482652537e-11	6.40943734134472e-10	5.34328012277146e-10	RAD54B/TOP2A/MCM2/BRIP1/RFC4/MCM8/GINS1/MCM4/MCM3/GINS2/MCM5	11
**GO:0008608**	GO:0008608	attachment of spindle microtubules to kinetochore	9/120	49/18870	2.08202630862049e-11	8.64841697426973e-10	7.20982389732818e-10	ECT2/CCNB1/KNTC1/NUF2/NDC80/KIF2C/CDCA8/CDT1/NEK2	9
**GO:0006275**	GO:0006275	regulation of DNA replication	12/120	124/18870	2.18555507860426e-11	8.85149806834724e-10	7.37912412065595e-10	E2F7/MCM2/CCNA2/RFC4/MCM4/CDK2/MCM3/PCNA/METTL4/MCM5/CDT1/CDK1	12

**GO:1905818**	GO:1905818	regulation of chromosome separation	10/120	74/18870	3.7703478685249e-11	1.46811812891883e-09	1.22390874555091e-09	SMC4/CENPF/CCNB1/KNTC1/NUF2/NDC80/CDC20/BUB1B/CDCA8/NCAPG2	10
**GO:0051383**	GO:0051383	kinetochore organization	7/120	21/18870	3.80623218608585e-11	1.46811812891883e-09	1.22390874555091e-09	SMC4/CENPF/KNTC1/CENPK/NUF2/NDC80/CENPH	7
**GO:0051983**	GO:0051983	regulation of chromosome segregation	12/120	131/18870	4.19529597940929e-11	1.58055336898676e-09	1.31764130760884e-09	SMC4/CENPF/CCNB1/KNTC1/NUF2/NDC80/CDC20/KIF2C/BUB1B/CDCA8/CDK1/NCAPG2	12
**GO:0051225**	GO:0051225	spindle assembly	12/120	133/18870	5.01890233598949e-11	1.84786858734158e-09	1.5404908366207e-09	ASPM/PRC1/STIL/NDC80/KIF4A/HAUS1/TPX2/CDC20/KIFC1/CDCA8/NEK2/CCNB2	12
**GO:0033047**	GO:0033047	regulation of mitotic sister chromatid segregation	9/120	54/18870	5.24811982880034e-11	1.88932313836812e-09	1.57504976382476e-09	CENPF/CCNB1/KNTC1/NUF2/NDC80/CDC20/BUB1B/CDCA8/CDK1	9
**GO:0006270**	GO:0006270	DNA replication initiation	8/120	37/18870	6.98954240817432e-11	2.46153450027009e-09	2.05207846903608e-09	MCM2/POLA1/MCM4/ORC6/CDK2/MCM3/MCM5/CDT1	8
**GO:0051304**	GO:0051304	chromosome separation	10/120	81/18870	9.49800057827045e-11	3.273778922723e-09	2.729212708157e-09	SMC4/CENPF/CCNB1/KNTC1/NUF2/NDC80/CDC20/BUB1B/CDCA8/NCAPG2	10
**GO:0006302**	GO:0006302	double-strand break repair	16/120	307/18870	1.10442129645464e-10	3.72742187553442e-09	3.10739588454234e-09	RAD54B/MCM2/BRCA1/BRIP1/POLA1/MCM8/MCM4/RFWD3/MCM3/RAD51AP1/GINS2/FOXM1/MCM5/EME1/FANCD2/DEK	16
**GO:0006271**	GO:0006271	DNA strand elongation involved in DNA replication	6/120	15/18870	2.78468295137694e-10	9.02237276246129e-09	7.52157521392971e-09	RFC4/POLA1/GINS1/MCM4/MCM3/PCNA	6
**GO:0030174**	GO:0030174	regulation of DNA-templated DNA replication initiation	6/120	15/18870	2.78468295137694e-10	9.02237276246129e-09	7.52157521392971e-09	MCM2/MCM4/CDK2/MCM3/MCM5/CDT1	6
**GO:0044786**	GO:0044786	cell cycle DNA replication	8/120	44/18870	3.09198103515379e-10	9.82158681754735e-09	8.18784658019054e-09	E2F7/MCM2/POLA1/GINS1/MCM4/MCM3/PCNA/CDT1	8
**GO:0007094**	GO:0007094	mitotic spindle assembly checkpoint signaling	8/120	46/18870	4.5043128547356e-10	1.32986019286869e-08	1.10864888073459e-08	CENPF/CCNB1/KNTC1/NUF2/NDC80/CDC20/BUB1B/CDCA8	8
**GO:0071173**	GO:0071173	spindle assembly checkpoint signaling	8/120	46/18870	4.5043128547356e-10	1.32986019286869e-08	1.10864888073459e-08	CENPF/CCNB1/KNTC1/NUF2/NDC80/CDC20/BUB1B/CDCA8	8
**GO:0071174**	GO:0071174	mitotic spindle	8/120	46/18870	4.5043128547356e-10	1.32986019286869e-08	1.10864888073459e-08	CENPF/CCNB1/KNTC1/NUF2/NDC80/CDC20/BUB1B/CDCA8	8

		checkpoint signaling							
**GO:0035825**	GO:0035825	homologous recombination	9/120	68/18870	4.51495744492456e-10	1.32986019286869e-08	1.10864888073459e-08	RAD54B/TOP2A/PSMC3IP/BRCA1/BRIP1/RAD51AP1/EME1/FANCD2/BARD1	9
**GO:0006310**	GO:0006310	DNA recombination	16/120	340/18870	4.94667266868492e-10	1.43100173629814e-08	1.19296635975992e-08	RAD54B/TOP2A/MCM2/PSMC3IP/BRCA1/BRIP1/MCM8/MCM4/RFWD3/MCM3/RAD51AP1/GINS2/MCM5/EME1/FANCD2/BARD1	16
**GO:0031577**	GO:0031577	spindle checkpoint signaling	8/120	47/18870	5.3996451790044e-10	1.53463599824336e-08	1.27936191406512e-08	CENPF/CCNB1/KNTC1/NUF2/NDC80/CDC20/BUB1B/CDCA8	8
**GO:0033044**	GO:0033044	regulation of chromosome organization	14/120	247/18870	5.85969933686637e-10	1.63667464236612e-08	1.36442726845727e-08	SMC4/CENPF/TOP2A/CCNB1/KNTC1/NUF2/NDC80/CDC20/CDK2/BUB1B/CDCA8/NEK2/CDK1/NCAPG2	14
**GO:0007127**	GO:0007127	meiosis I	11/120	129/18870	6.11187225191407e-10	1.657388826477e-08	1.38169581830409e-08	RAD54B/TOP2A/PSMC3IP/BRIP1/NDC80/PTTG1/RAD51AP1/EME1/FANCD2/CCNB2/MYBL1	11
**GO:0033046**	GO:0033046	negative regulation of sister chromatid segregation	8/120	48/18870	6.44540099185501e-10	1.657388826477e-08	1.38169581830409e-08	CENPF/CCNB1/KNTC1/NUF2/NDC80/CDC20/BUB1B/CDCA8	8
**GO:0033048**	GO:0033048	negative regulation of mitotic sister chromatid segregation	8/120	48/18870	6.44540099185501e-10	1.657388826477e-08	1.38169581830409e-08	CENPF/CCNB1/KNTC1/NUF2/NDC80/CDC20/BUB1B/CDCA8	8
**GO:0045841**	GO:0045841	negative regulation of mitotic metaphase/anaphase transition	8/120	48/18870	6.44540099185501e-10	1.657388826477e-08	1.38169581830409e-08	CENPF/CCNB1/KNTC1/NUF2/NDC80/CDC20/BUB1B/CDCA8	8
**GO:2000816**	GO:2000816	negative regulation of mitotic sister chromatid separation	8/120	48/18870	6.44540099185501e-10	1.657388826477e-08	1.38169581830409e-08	CENPF/CCNB1/KNTC1/NUF2/NDC80/CDC20/BUB1B/CDCA8	8
**GO:0051985**	GO:0051985	negative regulation of chromosome segregation	8/120	50/18870	9.07381609652607e-10	2.22720940551095e-08	1.85673142772615e-08	CENPF/CCNB1/KNTC1/NUF2/NDC80/CDC20/BUB1B/CDCA8	8
**GO:1902100**	GO:1902100	negative regulation of metaphase/anaphase transition of cell cycle	8/120	50/18870	9.07381609652607e-10	2.22720940551095e-08	1.85673142772615e-08	CENPF/CCNB1/KNTC1/NUF2/NDC80/CDC20/BUB1B/CDCA8	8

**GO:1905819**	GO:1905819	negative regulation of chromosome separation	8/120	50/18870	9.07381609652607e-10	2.22720940551095e-08	1.85673142772615e-08	CENPF/CCNB1/KNTC1/NUF2/NDC80/CDC20/BUB1B/CDCA8		8
**GO:0007098**	GO:0007098	centrosome cycle	11/120	138/18870	1.25985064516555e-09	3.04620603756447e-08	2.53949470188123e-08	PCLAF/CEP135/PLK4/BRCA1/STIL/NDC80/HAUS1/ODF2/CDK2/NEK2/CDK1		11
**GO:0051783**	GO:0051783	regulation of nuclear division	11/120	146/18870	2.29631159066982e-09	5.46061638417512e-08	4.55228773287633e-08	CENPF/NUSAP1/CCNB1/KNTC1/NUF2/NDC80/CDC20/BUB1B/RAD51AP1/CDCA8/NEK2		11
**GO:0045839**	GO:0045839	negative regulation of mitotic nuclear division	8/120	56/18870	2.32581808955607e-09	5.46061638417512e-08	4.55228773287633e-08	CENPF/CCNB1/KNTC1/NUF2/NDC80/CDC20/BUB1B/CDCA8		8
**GO:0007080**	GO:0007080	mitotic metaphase chromosome alignment	8/120	57/18870	2.69127726210621e-09	6.22838452087436e-08	5.19234395080039e-08	CCNB1/NUF2/NDC80/KIF14/KIF2C/KIFC1/CDCA8/CDT1		8
**GO:0006268**	GO:0006268	DNA unwinding involved in DNA replication	6/120	21/18870	2.92658065598666e-09	6.67755022915267e-08	5.56679463547945e-08	MCM2/GINS1/MCM4/MCM3/GINS2/MCM5		6
**GO:0010965**	GO:0010965	regulation of mitotic sister chromatid separation	8/120	59/18870	3.57360267412213e-09	8.04060601677479e-08	6.70311729663551e-08	CENPF/CCNB1/KNTC1/NUF2/NDC80/CDC20/BUB1B/CDCA8		8
**GO:0031023**	GO:0031023	microtubule organizing center organization	11/120	154/18870	4.03937963671682e-09	8.86110947479606e-08	7.38713674864415e-08	PCLAF/CEP135/PLK4/BRCA1/STIL/NDC80/HAUS1/ODF2/CDK2/NEK2/CDK1		11
**GO:0007088**	GO:0007088	regulation of mitotic nuclear division	10/120	118/18870	4.04766729095623e-09	8.86110947479606e-08	7.38713674864415e-08	CENPF/NUSAP1/CCNB1/KNTC1/NUF2/NDC80/CDC20/BUB1B/CDCA8/NEK2		10
**GO:0051306**	GO:0051306	mitotic sister chromatid separation	8/120	62/18870	5.36313150724847e-09	1.15843640556567e-07	9.65740031410496e-08	CENPF/CCNB1/KNTC1/NUF2/NDC80/CDC20/BUB1B/CDCA8		8
**GO:0033260**	GO:0033260	nuclear DNA replication	7/120	40/18870	5.52319323917169e-09	1.1773122430866e-07	9.81476028512088e-08	MCM2/POLA1/GINS1/MCM4/MCM3/PCNA/CDT1		7
**GO:0051784**	GO:0051784	negative regulation of nuclear division	8/120	63/18870	6.11083469718917e-09	1.28565613109694e-07	1.07179780129784e-07	CENPF/CCNB1/KNTC1/NUF2/NDC80/CDC20/BUB1B/CDCA8		8
**GO:0045132**	GO:0045132	meiotic chromosom	9/120	91/18870	6.35550686341385e-09	1.31998988701672e-07	1.1004204191309e-07	SMC4/PSMC3IP/ASPM/NUF2/BRIP1/NDC80/PTTG1/FANCD2/CCNB2		9

		e segregation							
**GO:0000727**	GO:0000727	double-strand break repair via break-induced replication	5/120	12/18870	7.30923420183875e-09	1.49885562113655e-07	1.24953330858882e-07	MCM2/MCM4/MCM3/GINS2/MCM5	5
**GO:2001251**	GO:2001251	negative regulation of chromosome organization	9/120	94/18870	8.48902414018291e-09	1.71902738838704e-07	1.43308131208614e-07	CENPF/TOP2A/CCNB1/KNTC1/NUF2/NDC80/CDC20/BUB1B/CDCA8	9
**GO:0031570**	GO:0031570	DNA integrity checkpoint signaling	10/120	135/18870	1.49427959980293e-08	2.98855919960586e-07	2.49143694158175e-07	DTL/BRCA1/BRIP1/RFWD3/CDK2/CDT1/EME1/FANCD2/BARD1/CDK1	10
**GO:0010389**	GO:0010389	regulation of G2/M transition of mitotic cell cycle	9/120	104/18870	2.07937871390317e-08	4.05854640544956e-07	3.38344057062494e-07	CENPF/DTL/CCNB1/BRCA1/KIF14/CDK2/BARD1/CDC25A/CDK1	9
**GO:0033045**	GO:0033045	regulation of sister chromatid segregation	9/120	104/18870	2.07937871390317e-08	4.05854640544956e-07	3.38344057062494e-07	CENPF/CCNB1/KNTC1/NUF2/NDC80/CDC20/BUB1B/CDCA8/CDK1	9
**GO:0034508**	GO:0034508	centromere complex assembly	6/120	30/18870	3.05629948391344e-08	5.89429186183307e-07	4.91382485947487e-07	CENPF/ITGB3BP/KNTC1/CENPK/CENPN/CENPH	6
**GO:0042770**	GO:0042770	signal transduction in response to DNA damage	11/120	189/18870	3.41797408039385e-08	6.51425648263299e-07	5.43066346148026e-07	DTL/E2F7/BRCA1/BRIP1/RFWD3/CDK2/FOXM1/EME1/FANCD2/BARD1/CDK1	11
**GO:1902969**	GO:1902969	mitotic DNA replication	5/120	16/18870	3.94993254526685e-08	7.44057060852593e-07	6.20289284648393e-07	MCM2/POLA1/GINS1/MCM4/MCM3	5
**GO:1902749**	GO:1902749	regulation of cell cycle G2/M phase transition	9/120	116/18870	5.41385334121875e-08	1.00809682905453e-06	8.40408207717321e-07	CENPF/DTL/CCNB1/BRCA1/KIF14/CDK2/BARD1/CDC25A/CDK1	9
**GO:0000082**	GO:0000082	G1/S transition of mitotic cell cycle	12/120	252/18870	7.29467980210736e-08	1.34288423629704e-06	1.11950648159136e-06	CDKN2C/E2F7/WEE1/EZH2/CCNA2/STIL/KIF14/RRM2/RFWD3/CDK2/CDC25A/CDK1	12
**GO:0007131**	GO:0007131	reciprocal meiotic recombination	7/120	60/18870	1.03016658103425e-07	1.85429984586165e-06	1.54585230814847e-06	RAD54B/TOP2A/PSMC3IP/BRIP1/RAD51AP1/EME1/FANCD2	7

**GO:0140527**	GO:0140527	reciprocal homologous recombination	7/120	60/18870	1.03016658103425e-07	1.85429984586165e-06	1.54585230814847e-06	RAD54B/TOP2A/PSMC3IP/BRIP1/RAD51AP1/EME1/FANCD2	7
**GO:0030071**	GO:0030071	regulation of mitotic metaphase/anaphase transition	8/120	90/18870	1.06044427366242e-07	1.88782387179465e-06	1.57379988792237e-06	CENPF/CCNB1/KNTC1/NUF2/NDC80/CDC20/BUB1B/CDCA8	8
**GO:0000077**	GO:0000077	DNA damage checkpoint signaling	9/120	126/18870	1.10893030577916e-07	1.95268162539374e-06	1.62786908731655e-06	DTL/BRCA1/BRIP1/RFWD3/CDK2/EME1/FANCD2/BARD1/CDK1	9
**GO:0022616**	GO:0022616	DNA strand elongation	6/120	37/18870	1.15397296167977e-07	2.01014644937767e-06	1.67577511016995e-06	RFC4/POLA1/GINS1/MCM4/MCM3/PCNA	6
**GO:1902099**	GO:1902099	regulation of metaphase/anaphase transition of cell cycle	8/120	93/18870	1.37132728572903e-07	2.36335127966067e-06	1.97022722014596e-06	CENPF/CCNB1/KNTC1/NUF2/NDC80/CDC20/BUB1B/CDCA8	8
**GO:0007091**	GO:0007091	metaphase/anaphase transition of mitotic cell cycle	8/120	94/18870	1.49099584799202e-07	2.54254028815481e-06	2.11960960994322e-06	CENPF/CCNB1/KNTC1/NUF2/NDC80/CDC20/BUB1B/CDCA8	8
**GO:0000724**	GO:0000724	double-strand break repair via homologous recombination	10/120	175/18870	1.75241801653904e-07	2.95720540290964e-06	2.46529859124955e-06	RAD54B/MCM2/BRCA1/MCM8/MCM4/RFWD3/MCM3/RAD51AP1/GINS2/MCM5	10
**GO:0051315**	GO:0051315	attachment of mitotic spindle microtubules to kinetochore	5/120	21/18870	1.79393073014184e-07	2.96547732941815e-06	2.47219455077549e-06	NUF2/NDC80/KIF2C/CDCA8/CDT1	5
**GO:0051988**	GO:0051988	regulation of attachment of spindle microtubules to kinetochore	5/120	21/18870	1.79393073014184e-07	2.96547732941815e-06	2.47219455077549e-06	ECT2/CCNB1/KNTC1/CDCA8/NEK2	5
**GO:0044784**	GO:0044784	metaphase/anaphase transition of cell cycle	8/120	97/18870	1.90532541966759e-07	3.11780523218333e-06	2.59918395899364e-06	CENPF/CCNB1/KNTC1/NUF2/NDC80/CDC20/BUB1B/CDCA8	8
**GO:0000725**	GO:0000725	recombinational repair	10/120	179/18870	2.16341460547243e-07	3.50473166086534e-06	2.92174835665382e-06	RAD54B/MCM2/BRCA1/MCM8/MCM4/RFWD3/MCM3/RAD51AP1/GINS2/MCM5	10

**GO:0044843**	GO:0044843	cell cycle G1/S phase transition	12/120	279/18870	2.19993675313414e-07	3.52861142581911e-06	2.9416559189902e-06	CDKN2C/E2F7/WEE1/EZH2/CCNA2/STIL/KIF14/RRM2/RFWD3/CDK2/CDC25A/CDK1	12
**GO:0090307**	GO:0090307	mitotic spindle assembly	7/120	71/18870	3.34852652255822e-07	5.31824800641599e-06	4.43360116454303e-06	PRC1/KIF4A/TPX2/CDC20/KIFC1/CDCA8/NEK2	7
**GO:1901976**	GO:1901976	regulation of cell cycle checkpoint	6/120	46/18870	4.43763731196333e-07	6.97958489842777e-06	5.81858832013179e-06	CCNB1/BRCA1/NDC80/RFWD3/CDCA8/BARD1	6
**GO:0051984**	GO:0051984	positive regulation of chromosome segregation	5/120	27/18870	6.90322079970344e-07	1.07530939379996e-05	8.96440514779303e-06	SMC4/CCNB1/CDCA8/CDK1/NCAPG2	5
**GO:1901989**	GO:1901989	positive regulation of cell cycle phase transition	8/120	119/18870	9.20536105866138e-07	1.42025570619347e-05	1.18400784343484e-05	DTL/CCNB1/EZH2/STIL/RRM2/CDC20/CDC25A/CDK1	8
**GO:0044773**	GO:0044773	mitotic DNA damage checkpoint signaling	7/120	83/18870	9.81687443466256e-07	1.50031477208994e-05	1.25074974177478e-05	DTL/BRCA1/RFWD3/CDK2/EME1/FANCD2/CDK1	7
**GO:0044771**	GO:0044771	meiotic cell cycle phase transition	4/120	13/18870	1.06387710682436e-06	1.61072982528548e-05	1.34279815844137e-05	NDC80/CDC20/CCNB2/CDC25A	4
**GO:0044774**	GO:0044774	mitotic DNA integrity checkpoint signaling	7/120	87/18870	1.35296159716146e-06	2.02944239574219e-05	1.69186133446213e-05	DTL/BRCA1/RFWD3/CDK2/EME1/FANCD2/CDK1	7
**GO:0045005**	GO:0045005	DNA-templated DNA replication maintenance of fidelity	6/120	56/18870	1.46060159235792e-06	2.1708023666237e-05	1.80970723611319e-05	BRCA1/MCM8/RFWD3/PCNA/EME1/BARD1	6
**GO:2000045**	GO:2000045	regulation of G1/S transition of mitotic cell cycle	9/120	183/18870	2.58541681878379e-06	3.80761386039068e-05	3.17424859186565e-05	CDKN2C/E2F7/WEE1/EZH2/STIL/KIF14/RRM2/RFWD3/CDK2	9
**GO:1901992**	GO:1901992	positive regulation of mitotic cell cycle phase transition	7/120	98/18870	3.02387440230537e-06	4.4132221006619e-05	3.67911887923925e-05	DTL/CCNB1/STIL/RRM2/CDC20/CDC25A/CDK1	7
**GO:0051054**	GO:0051054	positive regulation of DNA	11/120	298/18870	3.19763265703635e-06	4.62514723607043e-05	3.85579201031733e-05	E2F7/BRCA1/CCNA2/RFC4/CDK2/RAD51AP1/PCNA/FOXM1/CDT1/NEK2/CDK1	11

		metabolic process							
**GO:0051382**	GO:0051382	kinetochore assembly	4/120	17/18870	3.47225004838619e-06	4.97571649992035e-05	4.14804695867304e-05	CENPF/KNTC1/CENPK/CENPH	4
**GO:0010639**	GO:0010639	negative regulation of organelle organization	12/120	363/18870	3.50143012957358e-06	4.97571649992035e-05	4.14804695867304e-05	CENPF/TOP2A/CCNB1/KNTC1/NUF2/BRCA1/TRIOBP/NDC80/TPX2/CDC20/BUB1B/CDCA8	12
**GO:0045740**	GO:0045740	positive regulation of DNA replication	5/120	38/18870	4.05889762097882e-06	5.7177514312919e-05	4.76665047845842e-05	E2F7/CDK2/PCNA/CDT1/CDK1	5
**GO:0071897**	GO:0071897	DNA biosynthetic process	9/120	194/18870	4.17214198242922e-06	5.82661207890977e-05	4.85740305213856e-05	CENPF/DTL/PCLAF/CCNA2/RFC4/POLA1/TYMS/PCNA/NEK2	9
**GO:0036297**	GO:0036297	interstrand cross-link repair	5/120	40/18870	5.26717156590173e-06	7.29300678355624e-05	6.07987505087892e-05	FANCI/MCM8/RFWD3/RAD51AP1/FANCD2	5
**GO:0007099**	GO:0007099	centriole replication	5/120	43/18870	7.58893123425753e-06	0.000103957848896545	8.66653152269442e-05	CEP135/PLK4/BRCA1/STIL/CDK2	5
**GO:1902806**	GO:1902806	regulation of cell cycle G1/S phase transition	9/120	209/18870	7.63640988807953e-06	0.000103957848896545	8.66653152269442e-05	CDKN2C/E2F7/WEE1/EZH2/STIL/KIF14/RRM2/RFWD3/CDK2	9
**GO:0051298**	GO:0051298	centrosome duplication	6/120	75/18870	8.21024198924828e-06	0.000110838266854852	9.24012322123294e-05	CEP135/PLK4/BRCA1/STIL/NDC80/CDK2	6
**GO:0051656**	GO:0051656	establishment of organelle localization	13/120	469/18870	9.13744573894549e-06	0.000122336050389188	0.000101986453963176	CENPF/ECT2/NUSAP1/CCNB1/KNTC1/NUF2/NDC80/KIF14/KIF2C/KIFC1/CDCA8/CDT1/NEK2	13
**GO:0030261**	GO:0030261	chromosome condensation	5/120	47/18870	1.18503973456606e-05	0.000157357735245657	0.000131182569408823	SMC4/TOP2A/NUSAP1/CDK1/NCAPG2	5
**GO:0098534**	GO:0098534	centriole assembly	5/120	48/18870	1.31614452718191e-05	0.000173345864555666	0.000144511204824509	CEP135/PLK4/BRCA1/STIL/CDK2	5
**GO:0031297**	GO:0031297	replication fork processing	5/120	49/18870	1.45829408266136e-05	0.000190519065638016	0.000158827785064051	BRCA1/RFWD3/PCNA/EME1/BARD1	5
**GO:0045931**	GO:0045931	positive regulation of mitotic cell cycle	7/120	128/18870	1.76596205344689e-05	0.000228868682126718	0.000190798258069252	DTL/CCNB1/STIL/RRM2/CDC20/CDC25A/CDK1	7
**GO:0009314**	GO:0009314	response to radiation	12/120	428/18870	1.8390676672842e-05	0.000236451557216829	0.000197119784216499	RAD54B/DTL/PCLAF/ECT2/PBK/BRCA1/RFWD3/RAD51AP1/PCNA/FANCD2/BARD1/CDC25A	12

**GO:0010212**	GO:0010212	response to ionizing radiation	7/120	138/18870	2.87277132054234e-05	0.000366447995218787	0.000305492383 27856	RAD54B/ECT2/BRCA1/RFWD3/RAD51AP1/FANCD2/BARD1	7
**GO:0010971**	GO:0010971	positive regulation of G2/M transition of mitotic cell cycle	4/120	29/18870	3.26659975595556e-05	0.000410224155399071	0.000341986739036392	DTL/CCNB1/CDC25A/CDK1	4
**GO:1905820**	GO:1905820	positive regulation of chromosome separation	4/120	29/18870	3.26659975595556e-05	0.000410224155399071	0.000341986739036392	SMC4/CDC20/CDCA8/NCAPG2	4
**GO:0051256**	GO:0051256	mitotic spindle midzone assembly	3/120	11/18870	3.98718143266728e-05	0.000493071291673358	0.00041105293516369	PRC1/KIF4A/CDCA8	3
**GO:0085020**	GO:0085020	protein K6-linked ubiquitination	3/120	11/18870	3.98718143266728e-05	0.000493071291673358	0.00041105293516369	BRCA1/BARD1/UBE2T	3
**GO:1902751**	GO:1902751	positive regulation of cell cycle G2/M phase transition	4/120	32/18870	4.87345309725236e-05	0.000598105607390062	0.000498615655803411	DTL/CCNB1/CDC25A/CDK1	4
**GO:0000022**	GO:0000022	mitotic spindle elongation	3/120	12/18870	5.29156969790078e-05	0.000639727082880542	0.000533313740958892	PRC1/KIF4A/CDCA8	3
**GO:0090306**	GO:0090306	meiotic spindle assembly	3/120	12/18870	5.29156969790078e-05	0.000639727082880542	0.000533313740958892	ASPM/NDC80/CCNB2	3
**GO:0031100**	GO:0031100	animal organ regeneration	5/120	64/18870	5.40423073063908e-05	0.000648507687676689	0.000540633764320463	EZH2/CCNA2/TYMS/PCNA/CDK1	5
**GO:0006301**	GO:0006301	postreplication repair	4/120	34/18870	6.22368488839562e-05	0.000741350699941244	0.00061803310462938	DTL/PCLAF/BRCA1/PCNA	4
**GO:2000105**	GO:2000105	positive regulation of DNA-templated DNA replication	3/120	13/18870	6.84712429406627e-05	0.000809659953020975	0.000674979674935615	E2F7/CDK2/CDT1	3
**GO:0051231**	GO:0051231	spindle elongation	3/120	14/18870	8.67410058315998e-05	0.0010109383413467	0.000842777057795854	PRC1/KIF4A/CDCA8	3
**GO:0051255**	GO:0051255	spindle midzone assembly	3/120	14/18870	8.67410058315998e-05	0.0010109383413467	0.000842777057795854	PRC1/KIF4A/CDCA8	3

**GO:0009263**	GO:0009263	deoxyribonucleotide biosynthetic process	3/120	15/18870	0.000107923473018816	0.00122262955447889	0.0010192551776455	RRM2/TYMS/DTYMK	3
**GO:0009265**	GO:0009265	2'-deoxyribonucleotide biosynthetic process	3/120	15/18870	0.000107923473018816	0.00122262955447889	0.0010192551776455	RRM2/TYMS/DTYMK	3
**GO:0010457**	GO:0010457	centriole-centriole cohesion	3/120	15/18870	0.000107923473018816	0.00122262955447889	0.0010192551776455	CEP135/ODF2/NEK2	3
**GO:0046385**	GO:0046385	deoxyribose phosphate biosynthetic process	3/120	15/18870	0.000107923473018816	0.00122262955447889	0.0010192551776455	RRM2/TYMS/DTYMK	3
**GO:0000731**	GO:0000731	DNA synthesis involved in DNA repair	4/120	40/18870	0.000119089463700124	0.0013397564666264	0.00111689899069634	DTL/PCLAF/POLA1/PCNA	4
**GO:0065004**	GO:0065004	protein-DNA complex assembly	8/120	240/18870	0.000150542583939683	0.00168192404125715	0.00140214980177578	CENPF/MCM2/ITGB3BP/KNTC1/CENPK/CENPN/CDT1/CENPH	8
**GO:0071478**	GO:0071478	cellular response to radiation	7/120	181/18870	0.000159059477664261	0.00176490653298701	0.00147132883809118	ECT2/PBK/BRCA1/RAD51AP1/PCNA/BARD1/CDC25A	7
**GO:0000079**	GO:0000079	regulation of cyclin-dependent protein serine/threonine kinase activity	5/120	82/18870	0.000176573573284983	0.00194591284844675	0.0016222262407779	CDKN2C/CCNB1/CCNA2/CCNB2/CDC25A	5
**GO:0000910**	GO:0000910	cytokinesis	7/120	188/18870	0.000200903324491288	0.00219907693024247	0.00183327855848024	E2F7/ECT2/NUSAP1/PRC1/KIF14/KIF4A/CDCA8	7
**GO:1904029**	GO:1904029	regulation of cyclin-dependent protein kinase activity	5/120	85/18870	0.000209079137950357	0.00227320941932603	0.00189507971734587	CDKN2C/CCNB1/CCNA2/CCNB2/CDC25A	5
**GO:2000134**	GO:2000134	negative regulation of G1/S transition of mitotic cell cycle	5/120	86/18870	0.000220867306233073	0.00238536690731719	0.00198858072910198	E2F7/WEE1/EZH2/RFWD3/CDK2	5
**GO:0000212**	GO:0000212	meiotic spindle organization	3/120	19/18870	0.000225610668634888	0.0024045347578192	0.00200456016522549	ASPM/NDC80/CCNB2	3

**GO:0009148**	GO:0009148	pyrimidine nucleoside triphosphate biosynthetic process	3/120	19/18870	0.000225610668634888	0.0024045347578192	0.00200456016522549	TYMS/DTYMK/CTPS1	3
**GO:0006305**	GO:0006305	DNA alkylation	4/120	50/18870	0.00028575815813799	0.00300602737781522	0.00250599943192783	EZH2/METTL4/HELLS/DNMT1	4
**GO:0006306**	GO:0006306	DNA methylation	4/120	50/18870	0.00028575815813799	0.00300602737781522	0.00250599943192783	EZH2/METTL4/HELLS/DNMT1	4
**GO:0090231**	GO:0090231	regulation of spindle checkpoint	3/120	21/18870	0.000306800816538737	0.00316571543180098	0.00263912469070868	CCNB1/NDC80/CDCA8	3
**GO:0090266**	GO:0090266	regulation of mitotic cell cycle spindle assembly checkpoint	3/120	21/18870	0.000306800816538737	0.00316571543180098	0.00263912469070868	CCNB1/NDC80/CDCA8	3
**GO:1903504**	GO:1903504	regulation of mitotic spindle checkpoint	3/120	21/18870	0.000306800816538737	0.00316571543180098	0.00263912469070868	CCNB1/NDC80/CDCA8	3
**GO:0032465**	GO:0032465	regulation of cytokinesis	5/120	93/18870	0.00031816017244549	0.00326214860355503	0.00271951699698577	E2F7/ECT2/PRC1/KIF14/CDCA8	5
**GO:1902807**	GO:1902807	negative regulation of cell cycle G1/S phase transition	5/120	95/18870	0.000351142776898605	0.00357768112311786	0.00298256327547772	E2F7/WEE1/EZH2/RFWD3/CDK2	5
**GO:0044818**	GO:0044818	mitotic G2/M transition checkpoint	4/120	53/18870	0.000358046236185931	0.00362521814138255	0.00302219290149046	DTL/BRCA1/BARD1/CDK1	4
**GO:0046599**	GO:0046599	regulation of centriole replication	3/120	23/18870	0.00040475729438121	0.00407271314843205	0.00339525079235758	PLK4/BRCA1/STIL	3
**GO:2000001**	GO:2000001	regulation of DNA damage checkpoint	3/120	24/18870	0.000460440056861707	0.00460440056861707	0.00383849638046504	BRCA1/RFWD3/BARD1	3
**GO:0009147**	GO:0009147	pyrimidine nucleoside triphosphate metabolic process	3/120	25/18870	0.000520807771746788	0.0051133853953321	0.00426281576491949	TYMS/DTYMK/CTPS1	3
**GO:0019985**	GO:0019985	translesion synthesis	3/120	25/18870	0.000520807771746788	0.0051133853953321	0.00426281576491949	DTL/PCLAF/PCNA	3
**GO:1901985**	GO:1901985	positive regulation of	3/120	25/18870	0.000520807771746788	0.0051133853953321	0.00426281576491949	BRCA1/DEK/FAM161A	3

		protein acetylation							
**GO:2001252**	GO:2001252	positive regulation of chromosome organization	5/120	109/18870	0.000659798769850494	0.00643900004311927	0.00536792531210008	SMC4/CDK2/NEK2/CDK1/NCAPG2	5
**GO:0061351**	GO:0061351	neural precursor cell proliferation	6/120	166/18870	0.000676724839096178	0.00656463616368747	0.00547266289669333	ASPM/KIF14/POU3F3/SHCBP1/DMRTA2/MELK	6
**GO:0045143**	GO:0045143	homologous chromosome segregation	4/120	65/18870	0.000780700086253827	0.0074836339629065	0.00623879296582784	PSMC3IP/BRIP1/PTTG1/FANCD2	4
**GO:0070192**	GO:0070192	chromosome organization involved in meiotic cell cycle	4/120	65/18870	0.000780700086253827	0.0074836339629065	0.00623879296582784	SMC4/PSMC3IP/BRIP1/FANCD2	4
**GO:0097421**	GO:0097421	liver regeneration	3/120	29/18870	0.000812216703773235	0.0077399474124273	0.00645247077982081	EZH2/TYMS/PCNA	3
**GO:0051445**	GO:0051445	regulation of meiotic cell cycle	4/120	66/18870	0.000827022236132563	0.00783494750020322	0.00653166838386013	ASPM/CDC20/RAD51AP1/CDC25A	4
**GO:0010972**	GO:0010972	negative regulation of G2/M transition of mitotic cell cycle	4/120	67/18870	0.000875250838488051	0.00819599051069736	0.00683265485719604	DTL/BRCA1/BARD1/CDK1	4
**GO:0070301**	GO:0070301	cellular response to hydrogen peroxide	4/120	67/18870	0.000875250838488051	0.00819599051069736	0.00683265485719604	ECT2/EZH2/PCNA/CDK1	4
**GO:0001701**	GO:0001701	in utero embryonic development	9/120	392/18870	0.00090473180223242	0.00842336505526736	0.00702220751520989	E2F7/PLK4/CCNB1/STIL/RRM2/GINS1/NEK2/CCNB2/NCAPG2	9
**GO:2000573**	GO:2000573	positive regulation of DNA biosynthetic process	4/120	69/18870	0.000977598551542001	0.00902823862876048	0.00752646534158525	CCNA2/RFC4/PCNA/NEK2	4
**GO:0034502**	GO:0034502	protein localization to chromosome	5/120	119/18870	0.000980845678186324	0.00902823862876048	0.00752646534158525	KNTC1/EZH2/MCM8/BUB1B/CDK1	5

**GO:0045814**	GO:0045814	negative regulation of gene expression/epigenetic	5/120	120/18870	0.00101835767522016	0.00932056177320142	0.00777016293373452	EZH2/BRCA1/CDK2/HELLS/DNMT1	5
**GO:0071479**	GO:0071479	cellular response to ionizing radiation	4/120	70/18870	0.00103180327148179	0.00933810782011453	0.00778479033996553	ECT2/BRCA1/RAD51AP1/BARD1	4
**GO:1902750**	GO:1902750	negative regulation of cell cycle G2/M phase transition	4/120	70/18870	0.00103180327148179	0.00933810782011453	0.00778479033996553	DTL/BRCA1/BARD1/CDK1	4

Boruta

Boruta	ID	Description	GeneRatio	BgRatio	pvalue	p.adjust	qvalue	geneID	Count
**GO:1903046**	GO:1903046	meiotic cell cycle process	6/25	214/18870	2.93401097343123e-07	0.00013437770258315	8.33876802975193e-05	SMC4/FANCD2/PSMC3IP/MYBL1/NUF2/CDC25A	6
**GO:0007059**	GO:0007059	chromosome segregation	7/25	424/18870	9.32301426132583e-07	0.000185093816780685	0.000114859412849425	SMC4/RCC1/FANCD2/ITGB3BP/PSMC3IP/NUF2/CCNB1	7
**GO:0000280**	GO:0000280	nuclear division	7/25	441/18870	1.2124049134106e-06	0.000185093816780685	0.000114859412849425	SMC4/RCC1/FANCD2/PSMC3IP/MYBL1/NUF2/CCNB1	7
**GO:0051321**	GO:0051321	meiotic cell cycle	6/25	287/18870	1.63140526932673e-06	0.00018679590333791	0.000115915637557425	SMC4/FANCD2/PSMC3IP/MYBL1/NUF2/CDC25A	6
**GO:0048285**	GO:0048285	organelle fission	7/25	488/18870	2.37801421454743e-06	0.000217826102052544	0.000135171334300591	SMC4/RCC1/FANCD2/PSMC3IP/MYBL1/NUF2/CCNB1	7
**GO:0140013**	GO:0140013	meiotic nuclear division	5/25	194/18870	4.90313805846833e-06	0.000374272871796416	0.000232253908032711	SMC4/FANCD2/PSMC3IP/MYBL1/NUF2	5
**GO:0045132**	GO:0045132	meiotic chromosome	4/25	91/18870	5.92416231729734e-06	0.00038760947733174	0.000240529898597035	SMC4/FANCD2/PSMC3IP/NUF2	4

		segregation							
**GO:0090068**	GO:0090068	positive regulation of cell cycle process	5/25	262/18870	2.10206614854129e-05	0.00117530039463683	0.00072932913480107	SMC4/PLK4/E2F7/CCNB1/CDC25A	5
**GO:1901987**	GO:1901987	regulation of cell cycle phase transition	6/25	456/18870	2.30954226020339e-05	0.00117530039463683	0.00072932913480107	CDKN2C/E2F7/FANCD2/NUF2/CCNB1/CDC25A	6
**GO:0044772**	GO:0044772	mitotic cell cycle phase transition	6/25	470/18870	2.73801184983279e-05	0.00120878570054775	0.000750108340951261	CDKN2C/RCC1/E2F7/NUF2/CCNB1/CDC25A	6
**GO:0061982**	GO:0061982	meiosis I cell cycle process	4/25	136/18870	2.90319709738544e-05	0.00120878570054775	0.000750108340951261	FANCD2/PSMC3IP/MYBL1/CDC25A	4
**GO:0098813**	GO:0098813	nuclear chromosome segregation	5/25	312/18870	4.8450790428738e-05	0.00184920516803016	0.00114751872068064	SMC4/FANCD2/PSMC3IP/NUF2/CCNB1	5
	**ID**	**Description**	**GeneRatio**	**BgRatio**	**pvalue**	**p.adjust**	**qvalue**	**geneID**	**Count**
**GO:0000070**	GO:0000070	mitotic sister chromatid segregation	8/33	184/18870	7.90972856839e-10	5.1413235694535e-07	3.92988619397903e-07	SMC4/NDC80/NSL1/CENPK/NUF2/CCNB1/PRC1/KIF23	8

**GO:0000819**	GO:0000819	sister chromatid segregation	8/33	225/18870	3.87527427244681e-09	1.25946413854521e-06	9.62699713997313e-07	SMC4/NDC80/NSL1/CENPK/NUF2/CCNB1/PRC1/KIF23	8
**GO:0140014**	GO:0140014	mitotic nuclear division	8/33	274/18870	1.8089556301499e-08	3.91940386532478e-06	2.99588441203773e-06	SMC4/NDC80/NSL1/CENPK/NUF2/CCNB1/PRC1/KIF23	8
**GO:0051383**	GO:0051383	kinetochore organization	4/33	21/18870	4.54127068939776e-08	6.43380357487454e-06	4.91782232767738e-06	SMC4/NDC80/CENPK/NUF2	4
**GO:0098813**	GO:0098813	nuclear chromosome segregation	8/33	312/18870	4.94907967298041e-08	6.43380357487454e-06	4.91782232767738e-06	SMC4/NDC80/NSL1/CENPK/NUF2/CCNB1/PRC1/KIF23	8
**GO:0007059**	GO:0007059	chromosome segregation	8/33	424/18870	5.15993091422336e-07	5.5899251570753e-05	4.27278489739197e-05	SMC4/NDC80/NSL1/CENPK/NUF2/CCNB1/PRC1/KIF23	8
**GO:0000280**	GO:0000280	nuclear division	8/33	441/18870	6.94258135820006e-07	6.44668268975719e-05	4.92766676852696e-05	SMC4/NDC80/NSL1/CENPK/NUF2/CCNB1/PRC1/KIF23	8
**Cactus**									
**Cactus**	**ID**	**Description**	**GeneRatio**	**BgRatio**	**pvalue**	**p.adjust**	**qvalue**	**geneID**	**Count**
**GO:0007059**	GO:0007059	chromosome segregation	16/29	424/18870	1.65693569378286e-19	1.13831482162883e-16	6.83703991539876e-17	BRCA1/CENPF/CCNB1/NDC80/FANCD2/ITGB3BP/KIF14/BUB1B/NEK2/CENPK/KNTC1/KIF23/ASPM/ECT2/HAUS1/KIF2C	16
**GO:0098813**	GO:0098813	nuclear chromosome segregation	13/29	312/18870	2.87943469755497e-16	9.89085818610134e-14	5.94072842863974e-14	CENPF/CCNB1/NDC80/FANCD2/KIF14/BUB1B/NEK2/CENPK/KNTC1/KIF23/ASPM/ECT2/KIF2C	13

**GO:0000070**	GO:0000070	mitotic sister chromatid segregation	10/29	184/18870	1.03657618811655e-13	2.3737594707869e-11	1.42574689733926e-11	CENPF/CCNB1/NDC80/KIF14/BUB1B/NEK2/CENPK/KNTC1/KIF23/KIF2C	10
**GO:0000819**	GO:0000819	sister chromatid segregation	10/29	225/18870	7.81241038161553e-13	1.13889983098643e-10	6.84055364662038e-11	CENPF/CCNB1/NDC80/KIF14/BUB1B/NEK2/CENPK/KNTC1/KIF23/KIF2C	10
**GO:0000280**	GO:0000280	nuclear division	12/29	441/18870	8.28893617894052e-13	1.13889983098643e-10	6.84055364662038e-11	CENPF/CCNB1/NDC80/FANCD2/KIF14/BUB1B/NEK2/CENPK/KNTC1/KIF23/ASPM/KIF2C	12
**GO:1901987**	GO:1901987	regulation of cell cycle phase transition	12/29	456/18870	1.22873932602908e-12	1.285792091401e-10	7.72282999814894e-11	BRCA1/CENPF/CCNB1/NDC80/FANCD2/KIF14/BUB1B/EZH2/KNTC1/DTL/CDC25A/CDKN2C	12
**GO:0051310**	GO:0051310	metaphase chromosome alignment	8/29	96/18870	1.31012294611455e-12	1.285792091401e-10	7.72282999814894e-11	CENPF/CCNB1/NDC80/KIF14/NEK2/KNTC1/ECT2/KIF2C	8
**GO:1901990**	GO:1901990	regulation of mitotic cell cycle phase transition	11/29	355/18870	2.28895241813492e-12	1.96563788907336e-10	1.18061756303801e-10	BRCA1/CENPF/CCNB1/NDC80/KIF14/BUB1B/EZH2/KNTC1/DTL/CDC25A/CDKN2C	11
**GO:0048285**	GO:0048285	organelle fission	12/29	488/18870	2.7245902842683e-12	2.07977058365814e-10	1.24916887886921e-10	CENPF/CCNB1/NDC80/FANCD2/KIF14/BUB1B/NEK2/CENPK/KNTC1/KIF23/ASPM/KIF2C	12
**GO:0051303**	GO:0051303	establishment of chromosome	8/29	108/18870	3.4368638412853e-12	2.361125458963e-10	1.41815855345667e-10	CENPF/CCNB1/NDC80/KIF14/NEK2/KNTC1/ECT2/KIF2C	8

		localizati on							

Table S4. Results of KEGG analysis of bilogical pathways, results were corrected for multiple testing using Benjamini-Hochberg and an adjusted P − value < 0.05.

DGE											
	category	subcategory	ID	Description	GeneRatio	BgRatio	pvalue	p.adjust	qvalue	geneID	Count
**hsa04110**	Cellular Processes	Cell growth and death	hsa04110	Cell cycle	19/54	158/8842	2.20498369845578e-20	1.32299021907347e-18	9.98045252985248e-19	1031/891/890/7465/23594/10403/9232/5933/991/81620/701/983/4085/9319/699/9133/4173/4171/6502	19
**hsa03030**	Genetic Information Processing	Replication and repair	hsa03030	DNA replication	5/54	36/8842	2.29596613519564e-06	6.88789840558691e-05	5.19613388491644e-05	5984/3978/5422/4173/4171	5
**hsa03460**	Genetic Information Processing	Replication and repair	hsa03460	Fanconi anemia pathway	5/54	55/8842	1.94044474324372e-05	0.000292460485012155	0.000220628085184608	55215/672/2177/83990/29089	5
**hsa04114**	Cellular Processes	Cell growth and death	hsa04114	Oocyte meiosis	7/54	139/8842	1.9497365667477e-05	0.000292460485012155	0.000220628085184608	891/9232/991/983/4085/699/9133	7
**hsa04914**	Organismal Systems	Endocrine system	hsa04914	Progesterone-mediated oocyte maturation	6/54	111/8842	5.40715785124758e-05	0.00064885894214971	0.000489490079165571	891/890/983/4085/699/9133	6
**hsa05166**	Human Diseases	Infectious disease: viral	hsa05166	Human T-cell leukemia virus 1 infection	7/54	223/8842	0.000381598967373611	0.00381598967373611	0.00287872905211672	1031/890/9232/991/701/4085/9133	7
**hsa04115**	Cellular Processes	Cell growth	hsa04115	p53 signaling pathway	4/54	75/8842	0.00109571387833084	0.00939183324283578	0.0070850671831919	891/983/6241/9133	4

		and death									
**hsa05203**	Human Diseases	Cancer: overview	hsa05203	Viral carcinogenesis	6/54	205/8842	0.00147529713977118	0.0110647285482839	0.00834707592238959	890/5933/991/7188/983/6502	6
**hsa04120**	Genetic Information Processing	Folding, sorting and degradation	hsa04120	Ubiquitin mediated proteolysis	5/54	142/8842	0.00167324689108645	0.0111549792739096	0.00841515980312482	991/672/27338/6502/11065	5
**hsa03440**	Genetic Information Processing	Replication and repair	hsa03440	Homologous recombination	3/54	41/8842	0.00194816514475096	0.0116889908685058	0.00881801065518855	25788/672/83990	3
**hsa04218**	Cellular Processes	Cell growth and death	hsa04218	Cellular senescence	5/54	157/8842	0.00259800634311814	0.0141709436897353	0.0106903610290986	891/890/5933/983/9133	5
**hsa03430**	Genetic Information Processing	Replication and repair	hsa03430	Mismatch repair	2/54	23/8842	0.00853198533050782	0.0399553373705312	0.0301417457356639	5984/3978	2
**hsa04068**	Environmental Information Processing	Signal transduction	hsa04068	FoxO signaling pathway	4/54	133/8842	0.00865698976361509	0.0399553373705312	0.0301417457356639	891/10733/9133/6502	4
**hsa05170**	Human Diseases	Infectious disease: viral	hsa05170	Human immunodeficiency virus 1 infection	5/54	213/8842	0.00939420260817489	0.0402608683207495	0.0303722339963549	891/7465/7188/983/9133	5
STIR											
	**category**	**subcategory**	**ID**	**Description**	**GeneRatio**	**BgRatio**	**pvalue**	**p.adjust**	**qvalue**	**geneID**	**Count**
**hsa04110**	Cellular Processes	Cell growth	hsa04110	Cell cycle	19/58	158/8842	1.070641477395e-19	1.1562927955866e-17	1.00302201566479e-17	1031/4171/7465/891/890/10403/991/9232/4173/23594/1017/4172/701/5111/4174/81620/9133/993/983	19

		and death									
**hsa03030**	Genetic Information Processing	Replication and repair	hsa03030	DNA replication	7/58	36/8842	2.5910630049009e-09	1.39917402264648e-07	1.21370846019042e-07	4171/5984/5422/4173/4172/5111/4174	7
**hsa03460**	Genetic Information Processing	Replication and repair	hsa03460	Fanconi anemia pathway	6/58	55/8842	1.38231405896938e-06	4.97633061228978e-05	4.31670004379913e-05	672/83990/55215/146956/2177/29089	6
**hsa03440**	Genetic Information Processing	Replication and repair	hsa03440	Homologous recombination	5/58	41/8842	6.37425214120983e-06	0.000172104807812665	0.00014929169488623	25788/672/83990/146956/580	5
**hsa04218**	Cellular Processes	Cell growth and death	hsa04218	Cellular senescence	7/58	157/8842	6.84571482170143e-05	0.00144049598922686	0.00124955305108373	891/890/1017/2305/9133/993/983	7
**hsa04914**	Organismal Systems	Endocrine system	hsa04914	Progesterone-mediated oocyte maturation	6/58	111/8842	8.13600992757676e-05	0.00144049598922686	0.00124955305108373	891/890/1017/9133/993/983	6
**hsa05166**	Human Diseases	Infectious disease: viral	hsa05166	Human T-cell leukemia virus 1 infection	8/58	223/8842	9.33654807832225e-05	0.00144049598922686	0.00124955305108373	1031/890/991/9232/1017/701/9133/4792	8

STIR

	**category**	**subcategory**	**ID**	**Description**	**GeneRatio**	**BgRatio**	**pvalue**	**p.adjust**	**qvalue**	**geneID**	**Count**
**hsa04110**	Cellular Processes	Cell growth and death	hsa04110	Cell cycle	3/9	158/8842	0.000434538105231534	0.0108634526307884	0.00777594504098535	1031/891/993	3

Cactus

	category	subcategory	ID	Description	GeneRatio	BgRatio	pvalue	p.adjust	qvalue	geneID	Count
**hsa04110**	Cellular Processes	Cell growth and death	hsa04110	Cell cycle	6/14	158/8842	7.90029153481208e-08	2.44909037579175e-06	1.91270216105977e-06	891/10403/701/4171/993/1031	6
**hsa05206**	Human Diseases	Cancer: overview	hsa05206	MicroRNAs in cancer	4/14	312/8842	0.00114971835274707	0.0178206344675796	0.0139176432174646	672/2146/9493/993	4
**hsa03460**	Genetic Information Processing	Replication and repair	hsa03460	Fanconi anemia pathway	2/14	55/8842	0.00329550497439053	0.025631745047339	0.0200180012254261	672/2177	2

Table S5 . Results of univariate and mulitvariet Cox regression.

Univariate Cox regression	
DGE	SMC4, MCM8, E2F7, CCNB1, KIF4A, ECT2, RFC4, ASPM, LIG1, GJC1, RAD54B, SHOX2, CENPK, NDC80, PTTG1, NEMP1, BUB1B, CEP135, SGO2, SHCBP1, KIF23, BUB1, MYBL1, FAM161A, OFD1, MCM2, KIAA0754, NEK2, CRNDE, MEOX2
STIR	SMC4, MCM8, E2F7, CCNB1, KIF4A, ECT2, RFC4, ASPM, GJC1, RAD54B, SHOX2, CENPK, NDC80, PTTG1, NEMP1, BUB1B, CEP135, SHCBP1, MYBL1, FAM161A, MCM2, NEK2, CRNDE, CSMD3, TGIF1, TYMS, DUSP10, PCNA, PDPN, EME1, NCBP1, GAS2L3
Boruta	SMC4, MCM8, E2F7, CCNB1, GJC1, MYBL1, DZANK1, TTC26
RF	SMC4, CCNB1, GJC1, CENPK, NDC80, KIF23, DZANK1
Lasso	FAM161A, MNS1
Cactus	MCM8, CCNB1, ECT2, ASPM, GJC1, CENPK, NDC80, BUB1B, KIF23, MCM2, NEK2
**Mulitvariet Cox regression**	
DGE	SMC4, MCM8, E2F7, CCNB1, KIF4A, RFC4, ASPM, KIF14, NDC80, PTTG1, BUB1B, SGO2, SHCBP1, KIF23, BUB1, OIP5, NEK2, UBE2T
STIR	SMC4, MCM8, E2F7, CCNB1, KIF4A, RFC4, ASPM, KIF14, NDC80, PTTG1, BUB1B, SHCBP1, NEK2, UBE2T
Boruta	SMC4, MCM8, E2F7, CCNB1
RF	SMC4, CCNB1, NDC80, KIF23, SOX7
Cactus	MCM8, CCNB1, ASPM, KIF14, NDC80, BUB1B, KIF23, NEK2
